# Physical Activity Intervention for Leisure-Time Activity Levels Among Older Adults: A Cluster Randomized Trial

**DOI:** 10.1001/jamanetworkopen.2023.33195

**Published:** 2023-09-15

**Authors:** Nanyan Li, Qin Ye, Qian Deng, Yufei Wang, Julinling Hu, Xianlan Li, Qianqian Liu, Meili Jiang, Xing Zhao, Junmin Zhou

**Affiliations:** 1 West China School of Public Health and West China Fourth Hospital, Sichuan University, Chengdu, Sichuan, China

## Abstract

**Importance:**

Current randomized trial evidence of the effects of physical activity interventions in older adults is mainly from developed countries, with little reliable evidence from low- and middle-income countries, such as China, where race, culture, and lifestyles differ substantially from those in Western populations.

**Objective:**

To evaluate the effects of a multilevel intervention for increasing leisure-time activity levels in Chinese older adults.

**Design, Setting, and Participants:**

This cluster randomized trial was conducted from May 2021 to May 2023, including an 8-week intervention period and a follow-up period of 24 months. Eight villages in China were randomly assigned to the intervention (4 villages) or the control (4 villages) group. Potentially eligible participants were 60 years or older. A total of 511 older adults from the selected 8 villages were enrolled.

**Interventions:**

The intervention group received an 8-week socioecological model–based intervention, comprising individual, interpersonal, and community-level components, whereas the control group did not.

**Main Outcome and Measure:**

The primary outcome was the change in leisure-time activity at 8 weeks, measured by the Physical Activity Scale for the Elderly (PASE). Possible PASE scores for leisure-time activity range from 0 to 502, with higher scores indicating higher activity levels.

**Results:**

A total of 511 older adults from 8 villages were recruited and randomly allocated to the intervention (240 participants, 4 villages) or control (271 participants, 4 villages) groups. The mean (SD) age was 70.95 (5.72) years, and 284 (55.6%) were female participants. Seven serious adverse events (unrelated deaths) were reported. Participants in the intervention group increased leisure-time activity more than those in the control group, with a mean difference in PASE scores of 13.74 points (95% CI, 8.58-18.91 points) between the groups at 8 weeks (*P* < .001). Significant differences in leisure-time activity were also found over 24 months (mean difference in scores at 4 weeks, 11.66 points; 95% CI, 6.41-16.90 points; *P* < .001; at 6 months, 12.35 points; 95% CI, 7.19-17.50 points; *P* < .001; at 12 months, 11.55 points; 95% CI, 6.32-16.78 points; *P* < .001; and at 24 months, 14.51 points; 95% CI, 9.28-19.75 points; *P* < .001).

**Conclusions and Relevance:**

In this cluster randomized trial, the multilevel intervention was effective in promoting leisure-time activity over 24 months of follow-up in older adults from China. This finding suggests that implementation of such interventions could be an important step in addressing physical inactivity in older adults in low- and middle-income countries.

**Trial Registration:**

Chinese Clinical Trial Registry Identifier: ChiCTR2100045653

## Key Points

**Question:**

Dose a multilevel physical activity intervention promote physical activity in older adults?

**Findings:**

In this cluster randomized trial of 511 older adults in 8 villages in China, a multilevel physical activity intervention resulted in an increase in leisure-time activity over 24 months, a difference that was statistically significant.

**Meaning:**

Implementation of multilevel physical activity interventions could be an important step in addressing physical inactivity in older adults.

## Teaser

This cluster randomized trial evaluates the effects of a multilevel intervention for increasing leisure-time activity levels among older adults in China.

## Introduction

Nearly 23% of the total global burden of disease is caused by diseases in adults aged 60 years or older.^1^ As worldwide aging continues to intensify,^2^ the world is facing a substantial disease burden and a grave public health challenge. The World Health Organization (WHO) has proposed that older adults should accumulate at least 150 minutes of moderate- to vigorous-intensity physical activity weekly to prevent or delay onset of diseases. However, a large proportion of older adults do not meet the WHO physical activity recommendation.^3^

With older adults exhibiting low levels of physical activity, interventions that focus on improving physical activity levels in the real-life setting have received increasing attention in recent years.^4,5^ Although these interventions have shown promising results, most of them have been conducted in high-income developed countries,^6^ with little reliable evidence from low- and middle-income countries, such as China, where race, culture, and lifestyles (eg, physical activity pattern) differ substantially from those in Western populations. Therefore, the generalizability of the findings from such studies may be limited. Furthermore, these interventions use changes in absolute physical activity level as outcome measures,^7,8^ without considering the proportions meeting the WHO physical activity recommendations. Therefore, the primary aim of this cluster randomized trial was to evaluate the effects of a multilevel intervention, comprising individual, interpersonal, and community-level components, in increasing leisure-time physical activity levels in Chinese older adults.

## Methods

### Study Design

The Stay Active While Aging (SAWA) study was a single-blinded cluster randomized trial conducted in China with follow-up measures at 4 weeks, 8 weeks, 6 months, 12 months, and 24 months from baseline. Clusters (villages) were randomized to 1 of the 2 groups: intervention or control. Recruitment and baseline data collection began in May 2021. All data from baseline to 24 months had been collected by May 2023. Written or thumbprint informed consent was obtained from each participant before study commencement. The study protocol has been published previously^9^ and was approved by the Sichuan University Medical Ethical Review Board. This study followed the Consolidated Standards of Reporting Trials (CONSORT) reporting guideline. The trial protocol can be found in Supplement 1.

### Participants

Adults 60 years or older were invited to participate by the village staff through telephone calls and notifications from the WeChat (a social media software) group. Participants were eligible to participate in the study if they were able to answer telephone calls, walk 400 m in 15 minutes, walk without any assistance, and complete the Timed Up & Go test.^10^ Potential participants were excluded if they were previously diagnosed with a stroke, arthritis, Parkinson disease, severe pneumonia, or severe heart disease; had severe cognitive or hearing impairment, poor control of hypertension or diabetes, or major surgery in the past 3 years; were undergoing cancer treatment; or had fallen in the past year.

### Randomization and Masking

A multistage random sampling method was performed, and random selections were generated by using random.org (Randomness and Integrity Services Ltd). Eight villages (Guilin, Jianzheng, Qianfeng, Yixue, Xinsheng, Yijia, Huanglian, and Tiane), which have a minimum of 4 km of distance between them, were randomly selected from Sichuan, China. The 8 villages were further randomly assigned to the intervention (n = 4) or the control group (n = 4). A single-blind process was pursued. Participants and assessment team were blinded to the group assignment. Participants were asked not to discuss the intervention during the assessment.

### Interventions

The 8-week multilevel intervention was developed based on a socioecological model. Participants in the intervention received the multilevel intervention from the research team, whereas those in the control did not. The intervention was condensed to 3 levels,^11^ with intervention activities occurring at the individual, interpersonal, and community levels to promote physical activity change. The eFigure in Supplement 2 shows the conceptual framework of the intervention.

### Individual Level

Telephone counseling, printed material, and training sessions were used to improve individual factors, such as knowledge, beliefs, perceived barriers and benefits, self-regulation, self-efficacy, and skills. The participants received 8 telephone counseling sessions (1 per week). Through telephone counseling, the trained intervention team encouraged the participants to exercise; tailored the participants’ physical activity goal, which followed the global recommendations^12^; and provided personalized help. Participants were provided with printed material from the intervention team at baseline on the potential risks and benefits of physical activity, physical activity recommendations, local exercise resources, feasible physical activity for older adults, and safety tips for performing physical activity. Sports experts offered training sessions consisting of stretching exercises and Tai Chi 3 times (at baseline, 4 weeks, and 8 weeks). Tai Chi is a moderate-intensity exercise that is beneficial for ameliorating cognitive function and preventing clinical diseases.^13,14^

### Interpersonal Level

Peer groups were organized to operationalize collective efficacy, observational learning, and incentive motivation. Each peer group was formed based on the wishes of the participants, with 3 to 10 members, and was constructed to facilitate the motivation and engagement of participants. Each group leader was elected by all members in the group. The group leaders were responsible for organizing and facilitating the exercise. The peer group was also reminded to engage in group exercises through telephone calls once a week. Rewards (living goods not related to physical activity) were given to group members who achieved their group exercise goals and to the responsible group leaders who actively supervised the group exercise.

### Community Level

Social capital and environmental factors were employed through group sharing and coaching, respectively. Participants attended a group sharing 3 times (at baseline, 4 weeks, and 8 weeks) delivered by the intervention team to share the barriers and benefits of exercise they experienced. Participants were provided with a range of guidance by coaches, including identifying barriers and facilitators to physical activity in local settings and using environmental resources (eg, walking paths and open spaces for physical activity).

### Outcomes

The primary outcome was the changes in leisure-time activity at 8 weeks. Leisure-time activity was defined as exercise, sports, and physically active hobbies that are practiced in one’s leisure time. Secondary outcomes were household activity, work-related activity, total physical activity, the proportions of participants meeting WHO recommendations, and leisure-time sitting. The health outcomes included cognitive function (measured by Telephone Interview for Cognitive Status [TICS-10]; possible scores range from 0 to 20, with higher scores indicating better cognition), nighttime sleep quality (measured by Pittsburgh Sleep Quality Index [PSQI]; possible scores range from 0 to 21, with higher scores indicating poorer sleep quality), blood pressure, and self-rated health (measured by EuroQol-5D visual analog; possible scores range from 0 to 100, with higher scores indicating better self-reported health status). The assessment team who received uniform professional training conducted a face-to-face interview to collect the data. Each interview was conducted in a separate room of the village activity center. Data gathered at baseline, 4 weeks, 8 weeks, 6 months, 12 months, and 24 months after the baseline were used in the study.

Leisure-time activity, household activity, work-related activity, and total physical activity were measured by the Physical Activity Scale for the Elderly (PASE). The PASE is a reliable and valid instrument for the assessment of physical activity of older adults and has been widely used in China.^15^ The PASE scores were calculated by multiplying the activity weight by the activity frequency for each item.^16^ Possible scores for leisure-time activity ranged from 0 to 502, with higher scores indicating higher activity level. The total physical activity score was the sum score of leisure-time activity, household activity, and work-related activity. Furthermore, we converted the frequency (days per week) and duration (hours per day) into hours per day that participants spent on moderate-intensity sport and vigorous-intensity sport based on the hours per day conversion table.^17^ Participants were considered to meet the WHO physical activity recommendation (2.5 hours per week of moderate-intensity sport or 1.25 hours per week of vigorous-intensity sport) if they spent 0.357 hours per day or more time on moderate-intensity sport, 0.179 hours per day or more on vigorous-intensity sport, or an equivalent combination of moderate- and vigorous-intensity activity. Self-reported leisure-time sitting was identified through the question, “In the past week, how much time did you spend in total on sitting during your leisure time?”

### Statistical Analysis

The first step estimated, based on an effect size of approximately 0.42 reported by a prior meta-analysis,^18^ a 2-sided α of 0.05 and a power of 0.8, needing 90 participants per group (180 total participants). The second step adjusted the clustering effect. The sample size of 378 for the total was inflated by the design effect calculated previously.^9^ Assuming a 20% attrition rate, the final estimated sample size was 454 participants for 2 groups.

The primary analysis was based on intention-to-treat analysis, including all participants irrespective of missing data. The repeated measures of continuous outcomes were analyzed in the general linear mixed models, which included random cluster and participant effects. In addition to the trial group, assessment time, and their interaction terms, the models included age, sex, and the respective baseline value of the outcome of interest adjusted. To test the effect of the intervention on the binary outcomes of participants meeting the physical activity recommendations at 4 weeks, 8 weeks, 6 months, 12 months, and 24 months, generalized linear mixed models with an identity link were also conducted. The model assumed that missing values were missing at random.

Three subgroup analyses were conducted to examine the heterogeneity of intervention effects on leisure-time activity across age groups (60-69 vs ≥70 years), sex (male vs female), and body mass index (calculated as weight in kilograms divided by height in meters squared) at baseline (underweight or normal weight vs overweight or obesity). Intention-to-treat analysis using multiple imputation was also performed on primary and secondary outcomes to assess the robustness of our results. Five imputations were conducted using the mice package in R, version 4.2.0 (R Foundation for Statistical Computing). Statistical significance was determined by a 2-sided *P* < .05.

## Results

**Figure 1.**
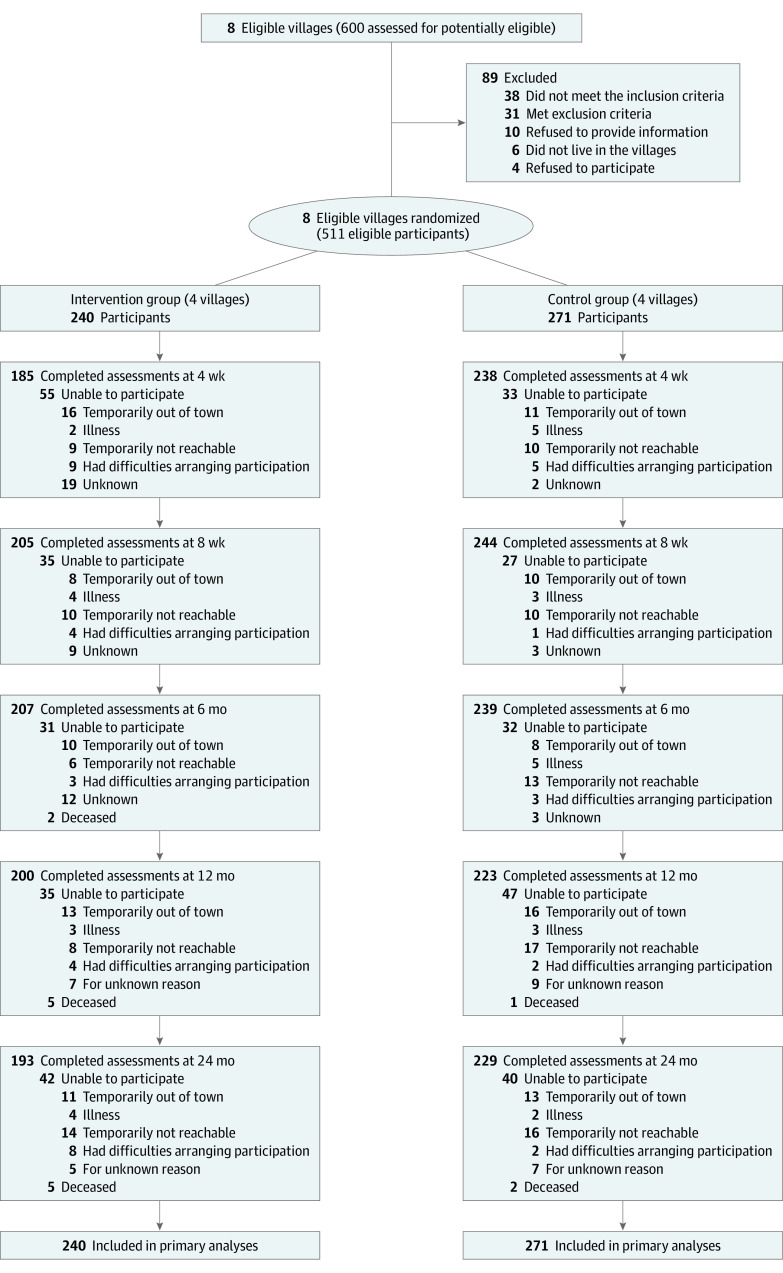
Flow Diagram of Participants in the Study

Figure 1 displays the flow of participants through the trial. Of the 600 older adults from 8 villages invited to participate in the SAWA study, 511 participants were assessed eligible in May 2021. Four villages were randomized to the intervention group (240 participants), and 4 villages were randomized to the control (271 participants). The proportions of participants attending follow-up visits were 85% (n = 205) for the intervention and 90% (n = 243) for the control at 8 weeks. eTable 1 in Supplement 2 shows the adherence to intervention domains. No cluster or participant dropout occurred during the study.

### Baseline Characteristics

Table 1 presents the characteristics of the 511 participants at baseline. The mean (SD) age was 70.95 (5.72) years; 284 were female participants (55.6%) and 227 were male participants (44.4%). Participants in the control group were more likely to participate in leisure-time activity (mean [SD] PASE score, 30.16 [26.49] vs 24.80 [21.28]), have better nighttime sleep quality (mean [SD] PSQI score, 5.41 [3.81] vs 6.75 [4.22]) and self-rated health (mean [SD] EuroQol-5D visual analog score, 78.64 [16.79] vs 74.03 [174.25]), and currently smoke (75 [27.7%] vs 38 [15.8%]) at baseline compared with participants in the intervention group.

**Table 1.** Baseline Characteristics of Participants

Characteristic	Overall (N = 511)	Intervention group (n = 240)	Control group (n = 271)
Demographic characteristic			
Age, mean (SD), y	70.95 (5.72)	70.61 (5.62)	71.25 (5.81)
Sex, No. (%)			
Male	227 (44.4)	95 (39.6)	132 (48.7)
Female	284 (55.6)	145 (60.4)	139 (51.3)
Marital status, No. (%)			
Cohabited	363 (71.0)	164 (68.3)	199 (73.4)
Did not cohabit	148 (29.0)	76 (31.7)	72 (26.6)
Educational level, No. (%)			
Illiteracy	158 (30.9)	85 (35.4)	73 (26.9)
Did not complete primary school	198 (38.7)	88 (36.7)	110 (40.6)
Primary school or above	155 (30.3)	67 (27.9)	88 (32.5)
Employment, No. (%)			
Yes	313 (61.3)	147 (61.3)	166 (61.3)
No	198 (38.7)	93 (38.8)	105 (38.7)
Income, ¥, No. (%)			
<12 000	229 (44.8)	115 (47.9)	114 (42.1)
12 000-19 999	154 (30.1)	69 (28.8)	85 (31.4)
≥20 000	128 (25.0)	56 (23.3)	72 (26.6)
Health and behaviors			
Smoking, No. (%)			
Never	335 (65.6)	172 (71.7)	163 (60.1)
Former	63 (12.3)	30 (12.5)	33 (12.2)
Current	113 (22.1)	38 (15.8)	75 (27.7)
Alcohol consumption, No. (%)			
Never or seldom	339 (66.3)	156 (65.0)	183 (67.5)
Less than once a month	34 (6.7)	18 (7.5)	16 (5.9)
Once a month or more	138 (27.0)	66 (27.5)	72 (26.6)
BMI, mean (SD)^a^	24.17 (3.27)	24.38 (3.48)	23.98 (3.05)
Nighttime sleep quality, mean (SD), PSQI score^b^	6.04 (4.06)	6.75 (4.22)	5.41 (3.81)
Blood pressure, mean (SD), mm Hg^c^			
Systolic	133.82 (19.67)	134.26 (18.78)	133.42 (20.47)
Diastolic	78.70 (11.21)	78.30 (10.81)	79.06 (11.57)
Self-rated health, mean (SD)^d^	76.48 (17.15)	74.03 (17.25)	78.64 (16.79)
Cognitive function, mean (SD), TICS-10 score^e^	8.71 (3.43)	8.47 (3.42)	8.92 (3.43)
Self-reported hypertension, No. (%)^f^	147 (28.8)	72 (30.0)	75 (27.7)
Self-reported diabetes, No. (%)^f^	53 (10.4)	23 (9.6)	30 (11.1)
Sitting and physical activity, mean (SD)			
Leisure-time activity, PASE score^g^	27.65 (24.31)	24.80 (21.28)	30.16 (26.49)
Household activity, PASE score^g^	78.25 (32.60)	80.54 (32.46)	76.22 (32.65)
Work-related activity, PASE score^g^	55.46 (65.47)	53.92 (63.46)	56.86 (67.34)
Total physical activity, PASE score^g^	161.07 (75.27)	158.87 (75.76)	163.08 (74.91)
Leisure-time sitting, min/d	160.74 (103.54)	160.86 (105.73)	160.63 (101.75)

Abbreviations: BMI, body mass index (calculated as weight in kilograms divided by height in meters squared); PASE, Physical Activity Scale for the Elderly; PSQI, Pittsburgh Sleep Quality Index; TICS-10, Telephone Interview for Cognitive Status.

^a^ Weight was determined using an electronic scale, and height was obtained by a portable stadiometer.

^b^ Measured by the PSQI. Scores range from 0 to 21, with higher scores indicating poorer sleep quality.

^c^ Blood pressure was measured by calibrated Omron U30 electronic sphygmomanometers from the right hands of participants while sitting down. Three measurements were taken at least 5 minutes apart, and the mean of the second of the 2 readings was calculated for systolic and diastolic pressure.

^d^ Measured by the EuroQol-5D visual analog scale. Scores range from 0 to 100, with higher scores indicating better self-reported health status.

^e^ Measured by the TICS-10. Scores range from 0 to 20, with higher scores indicating better cognition.

^f^ Self-reported by the participants based on whether they were diagnosed by a physician.

^g^ Possible scores for leisure-time activity, household activity, work-related activity, and total physical activity range from 0 to 502, 0 to 171, 0 to 168 or more, and 0 to 841 or more, respectively, with higher scores indicating higher activity level.

### Primary Outcome

**Figure 2.**
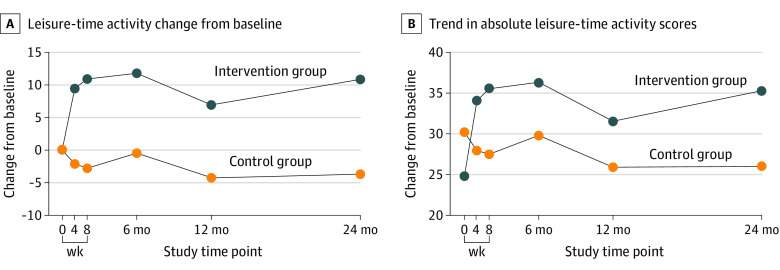
Change in Leisure-Time Activity by Each Time Assessment Point and Study Arm

The primary outcome was the change in leisure-time activity at 8 weeks. Participants in the intervention group increased leisure-time activity more than participants in the control group, with a mean difference in PASE score of 13.74 points (95% CI, 8.58-18.91 points) between the groups (*P* < .001). Significant differences in mean PASE scores for leisure-time activity were also found at 4 weeks (11.66 points; 95% CI, 6.41-16.90 points; *P* < .001), 6 months (12.35 points; 95% CI, 7.19-17.50 points; *P* < .001), 12 months (11.55 points; 95% CI, 6.32-16.78 points; *P* < .001), and 24 months (14.51 points; 95% CI, 9.28-19.75 points; *P* < .001) (Figure 2; eTable 2 in Supplement 2). Results were similar in sensitivity analyses that used multiple imputed data (eTable 3 in Supplement 2).

**Figure 3.**
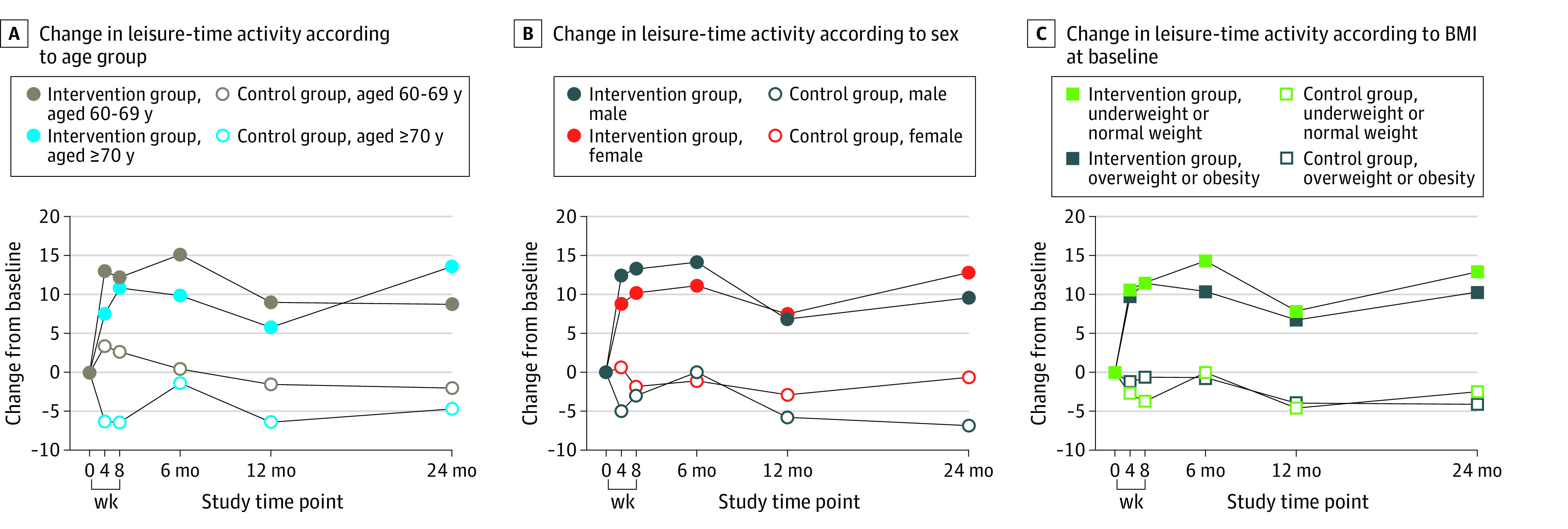
Change in Leisure-Time Activity From Baseline by Subgroup

The results of the subgroup analyses are shown in Figure 3. The leisure-time activity increase in the intervention group tended to be more apparent among participants aged 70 years or older than among younger participants (eTable 4 in Supplement 2). eTable 5 in Supplement 2 indicates that the intervention was more effective in male participants than in female participants. Subgroup analyses (eTable 6 in Supplement 2) showed a better increase in leisure-time activity among participants with underweight or normal weight.

### Secondary Outcomes

Table 2 presents the secondary outcomes. Differences were found between groups in the mean proportions of participants meeting WHO recommendations at 4 weeks (24.43%; 95% CI, 23.87%-25.00%; *P* < .001), 8 weeks (20.44%; 95% CI, 19.90%-20.98%; *P* < .001), 6 months (18.94%; 95% CI, 18.40%-19.49%; *P* < .001), 12 months (8.19%; 95% CI, 7.63%-8.75%; *P* < .001), and 24 months (5.58%; 95% CI, 5.31%-6.40%; *P* < .001]). Differences between groups were found in mean leisure-time sitting at 4 weeks (−41.72 min/d; 95% CI, −67.44 to −16.01 min/d; *P* = .002), 8 weeks (−36.34 min/d; 95% CI, −61.54 to −11.13 min/d; *P* = .005), and 24 months (−29.41 min/d; 95% CI, −55.08 to −3.75 min/d; *P* = .02) in favor of the intervention group compared with the control group. A significant difference in mean household activity PASE scores was only found at 6 months (9.67 points; 95% CI, 2.63-16.70 points; *P* = .007). There were statistically significant differences in mean total physical activity PASE scores at 4 weeks (20.78 points; 95% CI, 7.03-34.54 points; *P* = .003), 8 weeks (15.56 points; 95% CI, 2.00-29.11 points; *P* = .02), and 6 months (21.09 points; 95% CI, 7.59-34.58 points; *P* = .002) and no significant differences at 12 and 24 months. No differences were found for work-related activity at any time point. Similar results for secondary outcomes were seen in the intention-to-treat analysis using multiple imputation (eTable 7 in Supplement 2).

**Table 2.** Effects of the Intervention and Time on Secondary Outcomes^a^

Secondary outcome	Increase from baseline, mean (95% CI)		Intervention effect, mean (95% CI)	*P* value
	Intervention group	Control group		
**Proportions meeting WHO recommendations, %**				
At 4 wk	31.59 (31.05 to 32.13)	7.14 (6.60 to 7.68)	24.43 (23.87 to 25.00)	<.001
At 8 wk	23.56 (23.02 to 24.10)	3.00 (2.46 to 3.54)	20.44 (19.90 to 20.98)	<.001
At 6 mo	23.71 (23.18 to 24.25)	4.70 (4.16 to 5.34)	18.94 (18.40 to 19.49)	<.001
At 12 mo	9.11 (8.57 to 9.64)	0.85 (−0.31 to 1.39)	8.19 (7.63 to 8.75)	<.001
At 24 mo	2.75 (2.20 to 3.29)	−3.17 (−3.73 to −2.62)	5.58 (5.31 to 6.40)	<.001
**Leisure-time sitting, min/d**				
At 4 wk	4.59 (−14.47 to 23.65)	46.31 (29.05 to 63.58)	−41.72 (−67.44 to −16.01)	.002
At 8 wk	22.72 (4.24 to 41.20)	59.06 (41.92 to 76.19)	−36.34 (−61.54 to −11.13)	.005
At 6 mo	35.69 (17.26 to 54.11)	38.17 (20.91 to 55.43)	−2.48 (−27.73 to 22.77)	.85
At 12 mo	21.82 (3.20 to 40.44)	29.90 (12.30 to 47.49)	−8.08 (−33.70 to 17.54)	.54
At 24 mo	34.70 (15.88 to 53.51)	64.11 (46.65 to 81.57)	−29.41 (−55.08 to −3.75)	.02
**Household activity, PASE score**				
At 4 wk	3.02 (−2.28 to 8.32)	−0.47 (−5.29 to 4.34)	3.49 (−3.67 to 10.65)	.34
At 8 wk	1.39 (−3.77 to 6.55)	−3.42 (−8.21 to 1.36)	4.81 (−2.22 to 11.85)	.18
At 6 mo	3.85 (−1.27 to 8.98)	−5.82 (−10.63 to −1.00)	9.67 (2.63 to 16.70)	.007
At 12 mo	−2.25 (−7.43 to 2.93)	−4.62 (−9.53 to 0.29)	2.37 (−4.76 to 9.51)	.51
At 24 mo	0.006 (−5.23 to 5.24)	−5.71 (−10.57 to −0.84)	5.71 (−1.44 to 12.86)	.12
**Work-related activity, PASE score**				
At 4 wk	−8.75 (−16.92 to −0.58)	−14.27 (−21.78 to 6.76)	5.52 (−5.57 to 16.62)	.33
At 8 wk	−23.15 (−31.05 to −15.24)	−19.16 (−26.62 to −11.70)	−3.99 (−14.86 to 6.88)	.47
At 6 mo	−2.42 (−10.30 to 5.46)	−1.90 (−9.39 to 5.59)	−0.52 (−11.39 to 10.35)	.93
At 12 mo	7.64 (−0.31 to 15.59)	8.90 (1.45 to 16.35)	−1.26 (−12.28 to 9.76)	.82
At 24 mo	−7.87 (−15.92 to 0.19)	1.02 (−6.55 to 8.58)	−8.88 (−19.93 to 2.17)	.12
**Total physical activity, PASE score**				
At 4 wk	4.65 (−5.50 to 14.79)	−16.12 (−25.44 to −6.80)	20.78 (7.03 to 34.54)	.003
At 8 wk	−9.68 (−19.56 to 0.21)	−25.23 (−34.53 to −15.94)	15.56 (2.00 to 29.11)	.02
At 6 mo	13.80 (4.01 to 23.58)	−7.29 (−16.60 to 2.03)	21.09 (7.59 to 34.58)	.002
At 12 mo	13.04 (3.15 to 22.92)	0.42 (−9.06 to 9.90)	12.62 (−1.06 to 26.30)	.07
At 24 mo	3.15 (−6.84 to 13.15)	−8.87 (−18.17 to 0.61)	11.93 (−1.77 to 25.63)	.09

^a^ Model-based results from general and generalized linear mixed models. Calculated using time × group interaction term, indicating a differential increase from baseline to 4 weeks, baseline to 8 weeks, baseline to 6 months, baseline to 12 months, or baseline to 24 months. Covariates include age, sex, and the respective baseline value of the outcome of interest adjusted. All the values presented in the table are model-based estimates.

### Health Outcomes

The differential changes in health outcomes appear in eTable 8 in Supplement 2. Significant differences were found in PSQI scores for nighttime sleep quality at 8 weeks (−1.05 points; 95% CI, −1.71 to −0.40 points; *P* = .002), 6 months (−0.68 points; 95% CI, −1.33 to −0.02 points; *P* = .04), and 12 months (−0.88 points; 95% CI, −1.54 to −0.21 points; *P* = .01) between the intervention and control groups but not at 4 weeks or 24 months. There was a significant difference in diastolic blood pressure at 12 months (−2.77 mm Hg; 95% CI, −4.56 to −0.98 mm Hg; *P* = .002). There were significant differences in EuroQol-5D scores for self-rated health change at 4 weeks (3.60 points; 95% CI, 0.43-6.76 points; *P* = .03), 8 weeks (4.07 points; 95% CI, 0.97-7.18 points; *P* = .01), 6 months (4.31 points; 95% CI, 1.19-7.42 points; *P* = .007), 12 months (3.33 points; 95% CI, 0.17-6.50 points; *P* = .04), and 24 months (5.06 points; 95% CI, 1.90-8.23 points; *P* = .002). No significant differences were found in cognitive function and systolic blood pressure at any time point. Seven serious adverse events (unrelated deaths) were reported, including 5 in the intervention group and 2 in the control group (eTable 9 in Supplement 2).

## Discussion

In this cluster randomized trial of adults aged 60 years or older in China, an 8-week multilevel intervention based on a socioecological model resulted in a statistically significant increase in leisure-time activity compared with the control during 24 months of follow-up. Results were also suggestive of statistically significant changes in secondary outcomes, including proportions of participants meeting WHO recommendations and increased total physical activity, although changes in the latter were not sustained for a longer term (12 and 24 months). Time spent in leisure-time sitting was lower in the intervention group at 4 weeks, 8 weeks, and 24 months. For household activity and work-related activity, no significant differences between groups were found.

Several randomized trials conducted in Western countries have examined the effects of physical activity intervention in older adults.^6^ Most of them demonstrated the effects of multilevel physical activity intervention for older adults, but the magnitude of the effect was weaker in those interventions without theoretical basis.^19^ This finding may reflect the fact that theory-based interventions were more effective.^19^ Furthermore, the improvement in health outcomes, including nighttime sleep quality and self-rated health (eTable 8 in Supplement 2), and global cognition and orientation in individuals with illiteracy^20^ indicate that the significant increase in leisure-time activity could be clinically relevant.

In line with previous studies,^21,22^ our findings revealed that male participants or those with underweight or normal weight could benefit more from the physical activity intervention. Future intervention studies may pay more attention to female adults and those with overweight or obesity, especially the latter, because overweight or obesity poses an elevated risk of developing chronic diseases. The subgroup analyses according to age indicated that participants aged 70 years or older had a greater increase in leisure-time activity than those aged 60 to 69 years in 24 months of follow-up. These positive effects are encouraging in light of previous findings that physical activity levels decline with age.^23^

Furthermore, the effects on proportions of participants meeting WHO recommendations diminished at 24 months, although absolute leisure-time activity levels maintained an increase during 24 months. A possible explanation could be that older adults tended to do more light-intensity sports (eg, walking) and less moderate- or vigorous-intensity sports over time. However, a previous study suggested that older adults who performed moderate- or vigorous-intensity sports below current WHO recommendations had a 22% reduction in mortality compared with those reporting 0 minutes per week of moderate- or vigorous-intensity sports.^24^ Future studies are therefore needed to focus more on how older adults can be encouraged to do more moderate- or vigorous-intensity sports.

### Strengths and Limitations

This study has several strengths. To our knowledge, it is one of the first cluster randomized trials to test the effect of a multilevel physical activity intervention in older adults from developing countries, so our study narrows this knowledge gap. Furthermore, the intervention was based on a socioecological model, which enabled the intervention to be more effective and scalable. In addition, previous studies have revealed that individuals with low income are rarely involved in physical activity intervention programs.^25^ Our findings based on participants with generally low income levels add to the body of literature. Finally, the interventions are attractive to rural older adults because of the lack of resources in these areas. Most of those invited were included in our study, thus increasing the generalizability of the findings.

This study also has several limitations. First, physical activity level was assessed by self-report and may have been subject to recall bias. However, the PASE scores obtained from the reliable and valid PASE questionnaire were highly consistent with the objective assessment of physical activity.^26^ Second, there were statistically significant differences in sex, leisure-time activity, smoking, nighttime sleep quality, and self-rated health between the 2 groups at baseline. However, such imbalances are common in cluster randomized trials and extremely difficult to control.^27^ Third, because the intervention involved multilevel strategies, the study design did not allow us to identify the most effective level of changing physical activity. Fourth, although our study recruited participants from the community setting and had a fully powered sample size, it is possible that generalizability to other diverse older populations could be limited.

## Conclusions

This cluster randomized trial demonstrates that the SAWA intervention, developed based on a socioecological model, was effective in promoting leisure-time activity during 24 months of follow-up in Chinese older adults. This finding suggests that implementation of such interventions could be an important step to address physical inactivity in older adults in low- and middle-income countries.

## Notes

Supplement 1.
Trial Protocol

Supplement 2. **eFigure.** Intervention Based on the Social-ecologic Model **eTable 1.** Attendance at Each Contact Point **eTable 2.** Effects of the Intervention and Time on Leisure-time Activity **eTable 3.** Effects of the Intervention and Time on Leisure-time Activity (Intention-to-Treat Methods Using Multiple Imputed Data) **eTable 4.** Differences in Leisure-time Activity According to Age Group **eTable 5.** Differences in Leisure-time Activity According to Sex **eTable 6.** Differences in Leisure-time Activity According to BMI **eTable 7.** Effects of the Intervention and Time on Secondary Outcomes of Participants (Intention-to-Treat Method Using Multiple Imputed Data) **eTable 8.** Effects of the Intervention and Time on Health Outcomes of Participants **eTable 9.** Details of Unexpected Serious Adverse Events

Supplement 3.
Data Sharing Statement

## References

[1] Prince MJ, Wu F, Guo Y, . The burden of disease in older people and implications for health policy and practice. Lancet. 2015;385(9967):549-562. doi:10.1016/S0140-6736(14)61347-7 25468153

[2] World Health Organization. Ageing and health. Accessed August 9, 2023. https://www.who.int/news-room/fact-sheets/detail/ageing-and-health

[3] Koyanagi A, Stubbs B, Smith L, Gardner B, Vancampfort D. Correlates of physical activity among community-dwelling adults aged 50 or over in six low- and middle-income countries. PLoS One. 2017;12(10):e0186992. doi:10.1371/journal.pone.0186992 29077744 PMC5659773

[4] Kerr J, Rosenberg D, Millstein RA, . Cluster randomized controlled trial of a multilevel physical activity intervention for older adults. Int J Behav Nutr Phys Act. 2018;15(1):32. doi:10.1186/s12966-018-0658-4 29609594 PMC5879834

[5] Crist K, Full KM, Linke S, . Health effects and cost-effectiveness of a multilevel physical activity intervention in low-income older adults; results from the PEP4PA cluster randomized controlled trial. Int J Behav Nutr Phys Act. 2022;19(1):75. doi:10.1186/s12966-022-01309-w 35761363 PMC9235144

[6] Zubala A, MacGillivray S, Frost H, . Promotion of physical activity interventions for community dwelling older adults: a systematic review of reviews. PLoS One. 2017;12(7):e0180902. doi:10.1371/journal.pone.0180902 28700754 PMC5507305

[7] Rowley TW, Lenz EK, Swartz AM, Miller NE, Maeda H, Strath SJ. Efficacy of an individually tailored, internet-mediated physical activity intervention in older adults: a randomized controlled trial. J Appl Gerontol. 2019;38(7):1011-1022. doi:10.1177/0733464817735396 29165018

[8] Barrett S, Begg S, O’Halloran P, Kingsley M. A physical activity coaching intervention can improve and maintain physical activity and health-related outcomes in adult ambulatory hospital patients: the Healthy4U-2 randomised controlled trial. Int J Behav Nutr Phys Act. 2020;17(1):156. doi:10.1186/s12966-020-01063-x 33256753 PMC7708221

[9] Li N, Wang Y, Deng Q, Hu J, Zhou J. A multilevel physical activity intervention among Chinese rural older adults (Stay Active While Aging): a study protocol for a clustered randomized controlled trial. Front Public Health. 2022;10:760457. doi:10.3389/fpubh.2022.760457 35592074 PMC9110770

[10] Shumway-Cook A, Brauer S, Woollacott M. Predicting the probability for falls in community-dwelling older adults using the Timed Up & Go Test. Phys Ther. 2000;80(9):896-903. doi:10.1093/ptj/80.9.896 10960937

[11] Rich P, Aarons GA, Takemoto M, . Implementation-effectiveness trial of an ecological intervention for physical activity in ethnically diverse low income senior centers. BMC Public Health. 2017;18(1):29. doi:10.1186/s12889-017-4584-1 28720079 PMC5516364

[12] Bull FC, Al-Ansari SS, Biddle S, . World Health Organization 2020 guidelines on physical activity and sedentary behaviour. Br J Sports Med. 2020;54(24):1451-1462. doi:10.1136/bjsports-2020-102955 33239350 PMC7719906

[13] Lan C, Chen SY, Lai JS. The exercise intensity of Tai Chi Chuan. Med Sport Sci. 2008;52:12-19. doi:10.1159/000134225 18487882

[14] Huston P, McFarlane B. Health benefits Of Tai Chi: what is the evidence? Can Fam Physician. 2016;62(11):881-890. 28661865 PMC9844554

[15] Ngai SP, Cheung RT, Lam PL, Chiu JK, Fung EY. Validation and reliability of the Physical Activity Scale for the Elderly in Chinese population. J Rehabil Med. 2012;44(5):462-465. doi:10.2340/16501977-0953 22549657

[16] Washburn RA, McAuley E, Katula J, Mihalko SL, Boileau RA. The physical activity scale for the elderly (PASE): evidence for validity. J Clin Epidemiol. 1999;52(7):643-651. doi:10.1016/S0895-4356(99)00049-9 10391658

[17] Physical Activity Scale for the Elderly. Administration and Scoring Instruction Manual. Accessed August 9, 2023. https://meetinstrumentenzorg.nl/wp-content/uploads/instrumenten/PASE-handl.pdf

[18] Conn VS, Valentine JC, Cooper HM. Interventions to increase physical activity among aging adults: a meta-analysis. Ann Behav Med. 2002;24(3):190-200. doi:10.1207/S15324796ABM2403_04 12173676

[19] Chase JA. Interventions to increase physical activity among older adults: a meta-analysis. Gerontologist. 2015;55(4):706-718. doi:10.1093/geront/gnu090 25298530 PMC4542588

[20] Li N, Deng Q, Yang Q, . Effect of physical activity intervention on cognitive function in China: a cluster randomized trial. Alzheimers Dement. Published online March 1, 2023. doi:10.1002/alz.13005 36856072

[21] Harris T, Kerry SM, Victor CR, . A primary care nurse-delivered walking intervention in older adults: PACE (pedometer accelerometer consultation evaluation)-Lift cluster randomised controlled trial. PLoS Med. 2015;12(2):e1001783. doi:10.1371/journal.pmed.1001783 25689364 PMC4331517

[22] Schröder H, Cárdenas-Fuentes G, Martínez-González MA, ; PREDIMED-Plus investigators. Effectiveness of the physical activity intervention program in the PREDIMED-Plus study: a randomized controlled trial. Int J Behav Nutr Phys Act. 2018;15(1):110. doi:10.1186/s12966-018-0741-x 30424822 PMC6234632

[23] Cunningham C, O’Sullivan R, Caserotti P, Tully MA. Consequences of physical inactivity in older adults: a systematic review of reviews and meta-analyses. Scand J Med Sci Sports. 2020;30(5):816-827. doi:10.1111/sms.13616 32020713

[24] Hupin D, Roche F, Gremeaux V, . Even a low-dose of moderate-to-vigorous physical activity reduces mortality by 22% in adults aged ≥60 years: a systematic review and meta-analysis. Br J Sports Med. 2015;49(19):1262-1267. doi:10.1136/bjsports-2014-094306 26238869

[25] Franco MR, Tong A, Howard K, . Older people’s perspectives on participation in physical activity: a systematic review and thematic synthesis of qualitative literature. Br J Sports Med. 2015;49(19):1268-1276. doi:10.1136/bjsports-2014-094015 25586911

[26] Washburn RA, Ficker JL. Physical Activity Scale for the Elderly (PASE): the relationship with activity measured by a portable accelerometer. J Sports Med Phys Fitness. 1999;39(4):336-340. 10726435

[27] Ivers NM, Halperin IJ, Barnsley J, . Allocation techniques for balance at baseline in cluster randomized trials: a methodological review. Trials. 2012;13:120. doi:10.1186/1745-6215-13-120 22853820 PMC3503622

