# Supplementary material for: Physical Activity Intervention for Leisure-Time Activity Levels Among Older Adults: A Cluster Randomized Trial
Source: JAMA Netw Open. 2023 Sep 15;6(9):e2333195. doi: 10.1001/jamanetworkopen.2023.33195 (PMC10504609; doi:10.1001/jamanetworkopen.2023.33195)
Supplement: Supplement 1. — Trial Protocol [file jamanetwopen-e2333195-s001.pdf]

Stay Active While Aging

(SAWA)Trial

PROTOCOL

Table of Contents Page

Original Protocol……………………………………………………………………2

Final Protocol……………………………………………………………………...60

Summary of Changes to the Original Protocol……………………………………...120

Stay Active While Aging

(SAWA)Trial

PROTOCOL

Original Version

TABLE OF CONTENTS

Specific Aims 5

Background and Significance 6

Background 6

Significance 7

Overview of Trial Design 9

Design 9

Randomization 10

Eligible Criteria 11

Inclusion Criteria 11

Exclusion Criteria 11

Interventions 12

Data Collection 16

Quantitative surveys 16

Qualitative Interviews 21

Outcomes 21

Quality Assurance and Control 22

Data Management and monitoring 23

Sample Size Computation 24

Data Analysis Plan 25

1. Primary Analyses 25

2. Secondary Analyses 25

3. Subgroup Analyses...26

Trial Organization 27

Timeline 28

Literature Cited 29

Appendices...33

Appendix A: SAWA Forms and Questionnaires 34

Appendix B: Intervention Materials 52

Appendix C: SAWA Recruitment Materials 59

Specific Aims

The study aims to explore the factors affecting the physical activity of rural older adults, and to develop a suitable PA intervention model for rural older adults based on a socio-ecological model to provide valuable information for future study designs. The specific aims are as follows:

(1) Creating and implementing a PA intervention model suitable for rural older adults.

(2) Evaluate the effect and sustainability of the PA intervention model to provide valuable information for future study designs and yield evidence to inform the decision-making of health policy.

Background and Significance

Background

(1) Improving the PA Level of Residents has Far-reaching Implications for National Chronic Disease Prevention and Control

In recent decades, chronic diseases have shown a high prevalence in most of the world's population, and their incidence is increasing every year.[1] Taking hypertension as an example, the prevalence of hypertension in China was 25.2% in 2012, 5 times (5.0%) that of 1959 and 1.33 times (19.0%) that of 2002, and the number of deaths due to hypertension accounted for 24.60% of all deaths in 2010,[2,3] with related health costs accounting for 6.61% of the direct economic burden.[4,5] Therefore, the burden of chronic diseases in China is increasingly serious, and the prevention and control work is very serious.

Numerous studies have shown that regular PA can significantly reduce the incidence of many chronic diseases.[6,7] For example, PA can reduce the risk of developing up to 26 types of cancer.[6] However, the PA of the Chinese population is not promising. In 2013, the regular PA rate of the adult population in China was only 18.7% (22.2% in urban areas and 14.3% in rural areas), compared to about 50% in the United States during the same period.[8] In addition, according to the China Health& Nutrition Survey, PA (including work-related PA and leisure-time PA) decreased by nearly one-third for adult males and 42% for females from 1991 to 2011.[9]

Therefore, increasing the PA level of the Chinese population has profound implications for chronic disease prevention and control.

(2) Conducting PA Interventions in Rural Older Adults is Critical

The Chinese rural older adults have a huge burden of chronic diseases. In the case of hypertension, previous studies indicated that the prevalence of hypertension in China's rural older population is as high as 76.4%[10] and is extremely poorly controlled.[11] In

addition, the prevalence of comorbidity with two or more chronic diseases exceeds 90%.[10] The high prevalence of chronic diseases also brings with it a huge health care burden.

In order to reduce the burden of chronic diseases, the WHO recommends that older adults should remain physically active.[12] However, the level of PA among the Chinese rural older population is worrying. As a result of changes in productive lifestyle, their PA has declined significantly. Firstly, as mechanization increases and older people age, the work-related PA level of older rural residents is rapidly diminishing.[13] Furthermore, the level of PA in leisure time of rural older adults has not increased despite the increase in leisure time,[9,13]and the popularity of entertainment activities such as television and mahjong has prevented many rural older adults from participating in PA.[13,14] Previous studies have shown that overall levels of leisure-time PA preferences and behaviors among older people in rural China are low,[15,16] and there is no evidence that this situation is being alleviated.

However, current studies on PA interventions focused on other populations,[17,18] with few studies concerning rural older adults in China. This suggests that research in this area is lagging.

Therefore, it is necessary to carry out studies on PA interventions for rural older adults, to prevent, control, or delay the onset of many chronic diseases, reduce the health economic burden, and inform future similar studies.

Significance

The SAWA study is a sustainable, pragmatic PA intervention in Chinese rural older adults. As described above, the Chinese rural older adults have a huge burden of chronic diseases, however, the PA level among the Chinese rural older population is worrying. Thus, there is an urgent need to develop and test the PA intervention strategies in rural older adults in China.

The SAWA study seeks to use the socio-ecological model to guide an intervention

to increase PA in rural older populations. It proposes to conceptualize an intervention model by integrating the socio-ecological model with constructs from Health Belief Model, Social Cognitive Theory, and Community Organization and Community Building theories, in the light of impact factors of PA behavior. It aims to change participants' PA at the individual level, interpersonal level, and community level. Telephone counseling, printed materials, training sessions, peer group, group sharing, and coaching will be provided and established as intervention measures. A comprehensive evaluation of the effectiveness of the intervention program will be carried out in order to create an effective PA intervention model for rural older adults in China.

If the intervention model is applied to other populations, it will help to increase the PA level of the populations, thereby reducing or delaying the onset of various chronic diseases, reducing the burden of diseases on society and families, and improving the quality of life. The study will also yield valuable information on the efficacy and effectiveness of PA across a broad spectrum of important health outcomes. The study will influence both clinical practice and public health policy, and will, therefore, benefit individuals and society.

Overview of Trial Design

Design

This study is a single-blinded cluster randomized controlled trial to test the effect and sustainability of the multilevel PA intervention to improve PA levels in rural older adults. There were 8 villages participating in total: 4 intervention sites and 4 control sites. RE-AIM (Research, Efficacy, Adoption, Implementation, and Maintenance) will be applied to evaluate the pre-post and intervention-control differences as well as external validity.

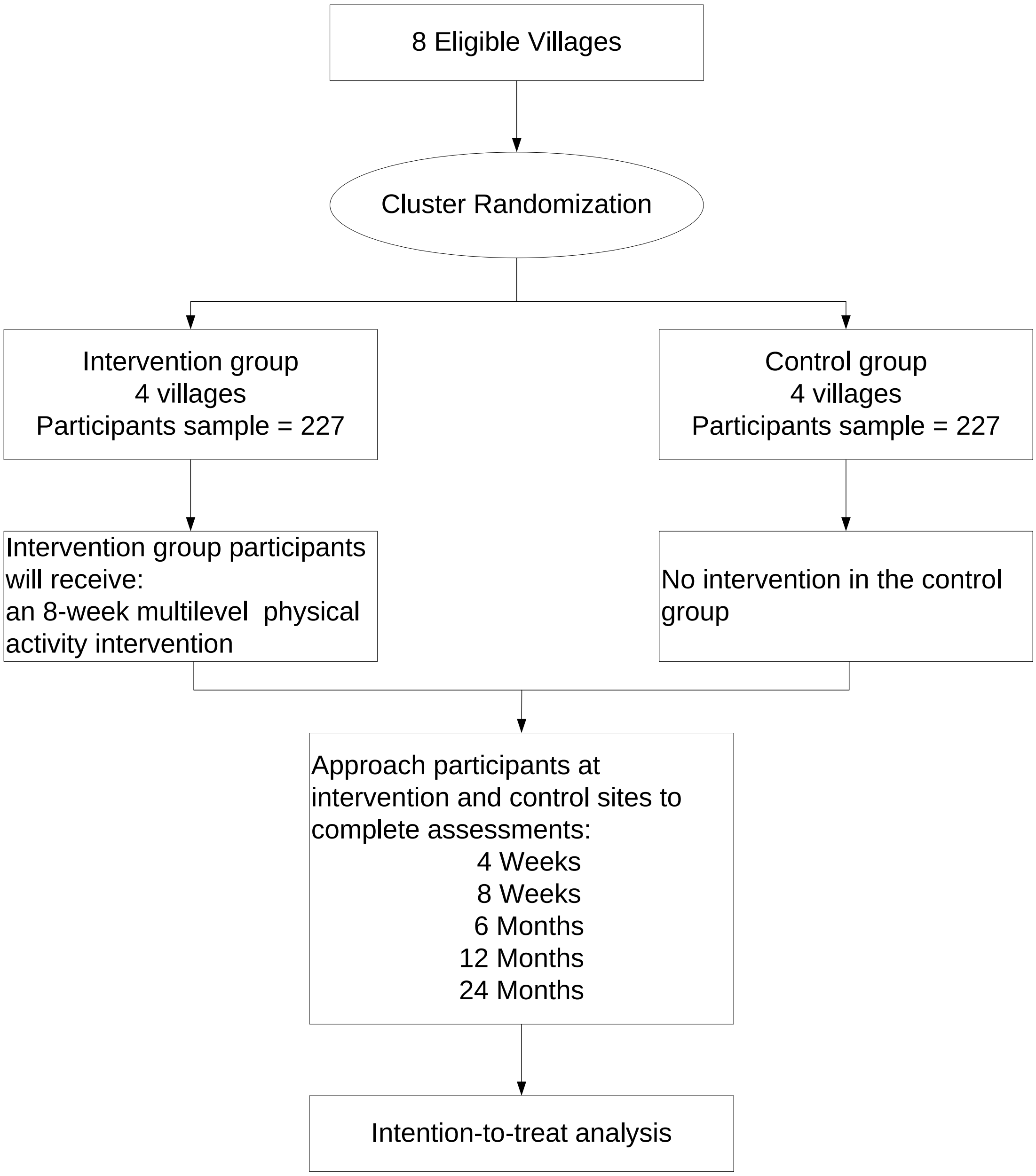

Figure 1. Overview of SAWA Intervention

Randomization

Multistage random sampling was used, and the random number was generated by the computer program (www.random.org). First, Chengdu was randomly selected from 18 cities in Sichuan Province. Second, Jianyang was selected out of 20 regions in Chengdu. Third, eight villages were randomly selected (Guilin, Jianzheng, Qianfeng, Yixue, Xinsheng, Yijia, Huanglian, Tiane), and these villages were further randomly assigned to either the intervention group or the control group (four villages for each group). There is a separation distance (minimum of 4 km) between each of these villages to minimize the risk of contamination.

Eligible Criteria

Inclusion Criteria

Participants are eligible to be included in the trial only if all of the following criteria apply:

(1) Be 60 years of age or older and

(2) Be able to answer phone calls and

(3) Be able to walk 400 m in 15 min and

(4) Be able to walk without the help of others or crutches and

(5) Be able to complete the Timed Up & Go test.[19]

Exclusion Criteria

Participants are excluded from the trial if any of the following criteria apply:

(1) Have a history of stroke, arthritis, Parkinson's disease, severe pneumonia or severe heart disease or

(2) Have severe cognitive or hearing impairment or

(3) Had major surgery in the past 3 years or

(4) Poor control of hypertension or diabetes or

(5) Be receiving cancer treatment or

(6) Have fallen in the past year.

Interventions

In light of current evidence on PA intervention and follow-up periods,[20-22] our project will consist of an 8-week intervention and a 24-month follow-up. The intervention group will receive the intervention from May to July 2021. Figure 2 shows the timeline of the 8-week intervention strategies.

Level	Intervention strategies	1 wk	2 wk	3 wk	4 wk	5 wk	6 wk	7 wk	8 wk
**Individual level**	**Telephone counseling**	×	×	×	×	×	×	×	×
	**Printed materials**	×	×	×	×	×	×	×	×
	**Training sessions**	×			×				×
**Interpersonal level**	**Peer group**	×	×	×	×	×	×	×	×
**Community level**	**Group sharing**	×			×				×
	**Coaching**	×			×				×

Figure 2. Timeline of the 8-week Intervention Strategies

The underlying theoretical model used to motivate the participants is derived from the socio-ecological model (SEM).[23] The SEM consists of five dimensions: individual, interpersonal, community, organizational, and public policy levels, which are thought to be the determinants of health-related behavior.[24,25] It emphasize the dynamic interaction among biological, psychological, behavioral, social, and environmental factors. Systematic reviews from intervention studies based on the SEM have revealed the effectiveness of such interventions, indicating that not only PA level was increased, but sedentary time was reduced.[26,27] For the current study, a condensed version of the SEM consisting of 3 levels of influence were utilized: individual, interpersonal, and community levels.[28,29] Our detailed interventions based on the three levels (individual, interpersonal, and community levels) are as follows:

1. Individual Level

On the individual level, telephone counseling, printed material, training sessions, and

self-monitoring will be employed to improve individual factors, such as knowledge, beliefs, perceived barriers and benefits, self-regulation, self-efficacy, and skills. Specifically, changes in perceived barriers, self-regulation, and self-efficacy will be achieved by telephone counseling; all the information printed in the materials will lead to improvements in knowledge, perceived benefits, and beliefs towards PA. The skills of PA will be improved through training sessions. Self-regulation and self-efficacy will also be achieved by self-monitoring.

Telephone Counseling

The participants will receive the feedback on their PA and health behaviors in relation to the recommendations through the telephone counseling. This feedback will also highlight the discrepancy between their heath and PA goals. The telephone call will be used to encourage participants to exercise and provide individualized assistance as follows:1) set the exercise goals based on their preference and the intensity of their daily exercise, 2) participants will be asked “did you achieve the exercise goals last week?”, 3) investigators will praise the respondents who have achieved the goals, and adjust them according to the global recommendations[30] on PA for older adults, 4) participants who did not achieve the goals will be encouraged, and investigators will assist them to solve the difficulties.

Printed Materials

Each participant in the intervention group will receive a booklet, which allows individuals to study information on their own and as need. Information provided in the printed materials include the potential risks of PA, the benefits of PA, the PA recommendation, local exercise resources for PA, the feasible PA for older adults, and safety tips for performing PA.

Training Sessions

The training sessions will be taught by sports experts who are certified to engage in physical education. Stretching exercises and Tai Chi will be mainly included in the

courses. Stretching exercise was a complement to other exercises. Tai Chi is a moderate-intensity exercise that has been supported in the literature for improving cognitive function and preventing clinical diseases, such as Parkinson’s disease, osteoarthritis, and so on[31,32]

2. Interpersonal Level

On the interpersonal level, peer groups will be organized to improve collective efficacy, observational learning, and incentive motivation. Collective efficacy will be enhanced by sharing group goals and communicating with each other. Each participant will learn new information and behaviors from other teammates by observing the behaviors, which will receive positive reinforcement. Further, the well-performing teammates and responsible leaders (encouraging teammates to participate in the group activities) will be rewarded to increase their motivation to participate in PA.

Peer Group

Peer groups will be established based on the wishes of participants. Each group will involve 3-10 members. Investigators will assist groups to set the group goals based on their common preferences. The group leader nominated by the group is responsible for reminding their members to exercise and organizing group activities. In order to enhance collective efficacy, observational learning, and incentive motivation, the members can communicate and learn from each other through the interactions. The peer groups will receive telephone calls once a week in the intervention period as well, and will be reminded to achieve the group goals by telephone. The well-performing teammates and responsible leaders will be rewarded.

3. Community Level

On the community level, group sharing and coaching will be formed to improve social capital and operationalize environmental factors. Participants will be encouraged to join the group sharing led by investigators, through which they can share their experiences and help each other in order to solve problems. Environmental factors will be

operationalized through the identification of harmful and beneficial factors in the environment and utilization of environmental resources with the help of coaches.

Group Sharing

Both the participants and investigators will join the group sharing. The group sharing will be led by the education team to discuss weekly topics, share stories with others in the group, and to engage in problem-solving together. The purpose of group sharing is to provide participants with opportunities to share their own exercise experiences with others, promote mutual trust, and increase social capital. For example, the participant who achieves the goals weekly will be asked “How did you keep exercising when the weather was terrible or the farming was busy?” Furthermore, participants will be encouraged to use a variety of supports including family and friends, as well as neighborhood and community supports.

Coaching

The participants will be guided by coaches to identify barriers to PA in rural settings and factors in the environment that are conducive to exercise. Coaches will also provide guidance on how to comprehensively utilize the environmental resources (e.g., walking paths, open spaces for PA) based on identified facilitating and constraining factors.

Data Collection

All the outcomes will be collected at baseline, 4 weeks, 8 weeks, 6, 12, and 24 months after the baseline. We will obtain written or thumbprint informed consent from each participant prior to the beginning of each interview. Both quantitative and qualitive data will be involved in the survey. The quantitative data will be obtained by the questionnaires or the anthropometry of each participant measured by trained technicians. The qualitive data will be obtained by the one-to-one interviews.

Quantitative surveys

The following data will be collected based on the questionnaires by trained investigators.

Physical Activity

The PA level of participants is the primary research outcome which will be measured by the Physical Activity Scale for the Elderly (PASE), a widely used instrument for older adults. Previous studies demonstrated the validity and reliability of the scale for assessing the PA level of older adults in the Chinese population.[33,34] PA is categorized into three domains in the scale: leisure time PA, household PA, and work-related PA. The score of each domain of the PA is calculated by multiplying the weight and frequency.[35,36]

Sedentary Behavior

Self-reported sedentary behavior will be assessed through the question, "In the past week, how much time did you spend in total on sitting during your leisure time?" Participants will be asked to further indicate the sedentary time for each behavior (playing cards, playing chess, reading, writing, socializing with friends or family, doing hobbies, driving, riding, time on public transport, and any other activities). When two or more activities are carried out at the same time, only the time for the main activity should be counted. For example, if you are watching TV and doing crafts, then count it

as TV time or craft time, but not both.

Self-efficacy

Self-efficacy will be obtained through the Self-Efficacy for Exercise Scale (SEE), which is suitable for older adults and has been tested the validity and reliability by a previous study.[37] Participants were asked to answer the questions in 9 different conditions, for example, "How confident are you that you could exercise three times per week and 20 minutes per time if the weather is not good?" The SEE score, ranging from 0-90, is scored by adding the score on each item (0-10), with higher scores indicating higher confidence levels on regular exercise.

Self-regulation

The 12-item Physical Activity Self-Regulation Scale (PASR-12) which is concise and validated for older adults will be used to assess self-regulation.[38] It comprises 12 items addressing the self-regulatory strategies, involving the following dimensions: self-monitoring, goal setting, eliciting social support, reinforcements, time management, and relapse prevention, using a 5-point response scale ("never = 1", "rarely = 2", "sometimes = 3", "often = 4", "very often = 5"). Each item has a score ranging from 1 to 5 and the PASR-12 can have a score ranging from 12 to 60.

Cognitive Function

Consistent with prior China Health and Retirement Longitudinal Study publications,[39,40] we will use the Telephone Interview for Cognitive Status (TICS-10), a questionnaire that assesses the individual's orientation, attention, and episodic memory.[41,42] Participants will be asked to reply the date (year, month, day), the day of the week, and season, serial subtractions of 7 from 100 five times, and immediate and delayed recall a list of Chinese nouns as many as they can. The orientation will be assessed by replying to the date, the day of the week, and the season, while the attention will be assessed by computing serial subtractions of 7 from 100 five times. Episodic memory will be assessed by immediate and delayed Chinese nouns recall. Immediate

recall refers to asking the participants to immediately recall as many nouns as they could after investigators read a list of 10 Chinese nouns. Delayed recall refers to asking the participants to recall as many of the original nouns as possible after a few minutes. TICS-10 scores will be calculated by summing the three measures above, and the possible total scores range from 0 to 5 for orientation, 0 to 5 for attention, and 0 to 10 for episodic memory with higher scores indicating better cognitive function.

Night-time Sleep Quality

The night-time sleep quality will be measured by the Pittsburgh Sleep Quality Index (PSQI).[43] The PSQI has been translated and adapted to Chinese populations, and has shown the validity and reliability in a previous study.[44] The PSQI is comprised of 7 dimensions (subjective sleep quality, sleep latency, sleep duration, habitual sleep efficiency, sleep disturbances, use of sleeping drugs, and daytime dysfunction). Participants will provide subjective sleep estimated based on the previous month. The answers will be used to generate scores for each of the 7 subcomponents, which ranged from 0 to 3. Overall sleep quality scores will be calculated as the sum of these factors (0-21), with a higher score indicating poorer sleep quality. A PSQI global score more than 5 is indicative a poor sleep quality.

The following outcomes will be based on the anthropometry of each participant measured by trained technicians.

Weight

Weight will be measured to the nearest 0.1 kg with the participants in light clothing and bare feet using Tanita BC-601 electronic scale.

Percentage Weight Change

Percentage weight change will be calculated as the percent of differences between each participant's measured weight at follow-up stages and at baseline.

Body Mass Index

Body mass index (BMI) is an individual's weight in kilograms divided by the square of height in meters. Height will be measured to the nearest 0.1cm with the participants in bare feet, back against the wall, heels together, and eyes looking straight ahead using portable stadiometers. According to BMI classification of the Chinese reference, participants can be categorized into four groups as following: underweight (BMI < 18.5 kg/m^2), normal weight (18.5 $kg/m^2 \leq$ BMI < 24.0 kg/m^2), overweight (24.0 $kg/m^2 \leq$ BMI < 28.0 kg/m^2), and obesity (BMI ≥ 28.0 kg/m^2).

Percentage Body Fat and Visceral Fat

Percentage body fat and visceral fat will be measured to the nearest 0.1% and 1, respectively, using Tanita BC-601 analyzer scales, with the participants wearing no shoes and socks. Participants will stand on a platform scale including electrodes, enabling the electric current to pass from one foot to the other. Through this process impedance is measured and the body fat percentage is calculated using a pre-established body fat predictive algorithm in the device, taking into account the age, gender, weight, height, and level of PA.

Waist Circumference

Waist circumference will be measured at the midpoint of the lowest rib margin and the upper margin of the iliac crest in the standing position[45]. The tape should be in contact with the skin but not compress soft tissue and twists in the tape should be avoided. The participants should be required to breathe normally. The same measuring tape should be used throughout the trial. Waist circumference will be recorded to the nearest 0.1 cm. According to the Chinese reference, abdominal obesity will be defined by waist circumference of at least 90 cm for men and 85 cm for women.

Hip Circumference

Hip circumference will be measured at the largest level of the symphysis pubis and gluteus maximus[45]. The tape should be in contact with the skin but not compress soft tissue, and twists in the tape should be avoided. The participants should be required to

breathe normally. The same measuring tape should be used throughout the trial. Hip circumference will be recorded to the nearest 0.1 cm.

Waist-to-hip Ratio

We will obtain the waist-to-hip ratio through waist circumference divided by hip circumference.

Waist-to-height Ratio

We will obtain the waist-to-height ratio through waist circumference divided by height.

Systolic and Diastolic Blood Pressure

Blood pressure measurements were performed with a calibrated Omron U30 electronic sphygmomanometers in the sitting position. Strictly following the America Heart Association's Standardized protocol,[46] measurements should be taken with an interval of minimum 5 minutes of the rest for the participants in a quiet setting without distractions. The three readings will be averaged to be recorded as the systolic or diastolic blood pressure.

Demographic Information and Health Related Questions

For descriptive purposes, the following participant socio-demographic characteristics, including sex (male/female), age, education level (illiterate, elementary, middle school, high school or above), marital status (married, never married, widowed, divorced), household income (<12000 RMB, 12000-19999 RMB, 20000-59999 RMB, ⩾ 60000 RMB), and employment (yes/no) will be collected at baseline.

Health behaviors including smoking status (never, former, current), alcohol consumption (never/seldom, < once a month, ⩾ once a month, daytime napping (0, 1-60 minutes/day, over 60 minutes/day) will also be obtained at baseline.

Self-reported medical history, including hypertension, diabetes mellitus, asthma, coronary heart disease, chronic bronchitis/emphysema, psychiatric disorders, and

malignant tumors will be collected at baseline.

Adverse Events

A surveillance form will be used to screen for adverse events (AEs) and serious adverse events at each assessment visit (see Appendix A). Serious, unexpected AEs that are related or are possibly related to the study will be reported to the Institutional Review Board (IRB) immediately.

Qualitative Interviews

We will obtain oral consent from each participant prior to the beginning of each interview. All interview will be recorded and transcribed. We will use RE-AIM to identify the domains, including: (1)Reach, participant perception of the program recruitment process; (2)Efficacy, is the intervention benefits for improving health benefits, the impact of intervention on participants; (3)Adoption, participants' evaluation of the adoption of each intervention; (4)Implementation, participants' satisfaction with the intervention program as a whole; (5)Maintenance, participants' willingness to continue performing PA after the end of the intervention and their overall attitude toward PA before and after the intervention.

Outcomes

Our primary outcome is the PA level of the participants. Our secondary outcomes include sedentary behavior, self-efficacy, self-regulation, cognitive function, night-time sleep quality, weight, percentage weight change, body mass index, percentage body fat, visceral fat, waist circumference, hip circumference, waist-to-hip ratio, waist-to-height ratio, and systolic and diastolic blood pressure.

Quality Assurance and Control

Quality assurance and quality control are of paramount importance in a randomized controlled trial. Standardized protocols for all measurements have been developed, and adherence to the written protocols is of utmost importance. All data collection personnel will be certified as competent to make the required measurements by trained experts. The following is a summary of the quality assurance and control program:

1. Personnel

Quality control of personnel is carried out in three main areas: a) Staff in surveys, interviews, and telephone counseling will be trained by professionals, with unified technical standards. b) Clear division of jobs, e.g. logistics during the investigation and coordination of personnel on site will be handled by different people. c) Responsibilities will be clearly defined, e.g. a person will be responsible for the safekeeping and storage of questionnaires.

2. Research Instruments

a) The study proposes to use internationally recognized research methods and tools (such as the PASE) to ensure the quality of the data. b) Intervention materials (e.g. dissemination materials) are developed by a team member specializing in physical education and sport, in consultation with relevant experts and with reference to national and international standards, in order to ensure the correctness and traceability of knowledge.

3. Quality Management System

a) Develop and implement a quality control program within the project team to ensure quality control at each key stage. b) Establish good external communication and feedback mechanisms with the team leaders of the peer groups and key figures of the villages to implement quality control.

Data Management and monitoring

Face-to-face interviews will be conducted to collect data by eligible assessment team at baseline, 4 weeks, 8 weeks, 6 months, 12 months, and 24 months after the baseline. The collected data will be transferred from paper form to electronic record through data entry by study personnel. Standardized approaches will be used to ensure the high-quality of data. Selected project managers will build the electronic database. Confidentiality of the database will be protected by using password-protected computers. Only those designated by the project manager can access the data using the encrypted computers under the supervision of management. The staff are trained to comply with and use protected health information.

The study team will conduct monthly quality monitoring checks. The database will only be shared among the study team. Emails will be encrypted if data will be sent between the project staff, however, emails will be minimized.

Sample Size Computation

Based on the prior PA intervention meta-analysis, the sample size required for each group was estimated to be about 90 with a power of 80%, an α of 0.05,and assumed an effect size of 0.42.[47] Because the randomization occurs at the village level, we need to consider the clustering effect.[48]

The sample size (SS) for a clustered RCT is defined by equation (1):

$$SS_{\text{cluster RCT}}=SS_{\text{standard RCT}}\times DE \quad (1)$$

The design effect (DE) is obtained from equation (2):

$$DE=1+(n-1)\times ICC \quad (2)$$

$$\text{Where: } ICC=\frac{\sigma_{between}^2}{\sigma_{between}^2+\sigma_{within}^2} \quad (3)$$

and n=cluster size (number of participants per cluster)

In equation (3), $\sigma_{between}^2$ is the between-cluster variance for the outcome measure and σ_{within}^2 is the within-cluster variance for the outcome measure.

We conducted a pilot study prior to the SAWA to calculate the DE. With a DE of 2.1, we will need a sample size of 189 (90×2.1) for each group after factoring in the cluster effects. Taking participation attrition into account, we will screen 20% more participants. Each group will have 227 participants, so the total sample size will be 454 participants for the two groups (intervention group and control group).

Data Analysis Plan

1. Primary Analyses

The primary aim will be to assess the PA level at 4 weeks, 8 weeks, 6 months, 12 months, and 24 months in the context of linear mixed model effects multilevel models. In this analysis, the intention-to-treat approach will be used. In addition to the intervention group, assessment time, and their interaction terms, the model may include individual-level (age, sex) variables as explanatory covariates. A random village intercept term is included to account for clustering by villages. The primary study hypothesis of SAWA will be tested based on a two-tailed significance level of 0.05.

Although substantial effort will be employed to minimize missing data, it is inevitable that some missing data will occur. Missing data are expected due to drop outs and missed visits. The PA level is a subject-level variable and missing assessments would influence tests relative to treatment-by-time interactions. The primary analysis described above will be conducted within each dataset using linear mixed effect models and employ restricted maximum likelihood using all available data. Results of datasets will be combined to produce final estimates for between-group comparisons. This approach assumes data are missing at random: the mechanism that gives rise to missing data relies on observed data only.

An intention-to-treat analysis that includes all randomized participants, regardless of the number of assessments obtained, will be conducted. The analytical plans are flexible and will be adapted as scientific perspectives are advanced.

2. Secondary Analyses

a. Compliance and As-Treated Analyses

Two additional analyses will be conducted to compare intervention groups accounting for compliance with intervention protocol: i) restricted to only those participants treated

as specified by the random assignment, and ii) with assigned intervention replaced with actual treatment administered (provided accurate data on actual intervention modality can be obtained). The results of these analyses will be compared with the intent-to-treat analyses.

b. Secondary Outcomes

The secondary outcome data including sedentary behavior, self-efficacy, self-regulation, cognitive function, night-time sleep quality, weight, percentage weight change, body mass index, percentage body fat, visceral fat, waist circumference, hip circumference, waist-to-hip ratio, waist-to-height ratio, and systolic and diastolic blood pressure will be collected at the baseline, 4-week, 8-week, 6-month, 12-month, and 24-month follow-up. Generalized linear mixed models were used to test the effect of the intervention on the binary outcomes. For continuous outcomes, analyses will be performed using a similar strategy as the primary analyses.

3. Subgroup Analyses

Modification of the effect of intervention upon primary and secondary outcomes will be performed separately in subgroups including age groups, sex, education level, household income, and BMI at baseline. Tests for key interactions (different intervention effects between sub-groups) will also be performed. However, it is recognized that the study may not be powered adequately to detect interactions; all subgroup analyses will be considered exploratory.

Trial Organization

The Principal Investigator will oversee and monitor progress to reach the milestones with decisions concerning short-term goals and the evaluation of longer-term progress being discussed with the research staff via weekly meetings. The research team will be organized into sub-teams of investigators and staff. The overall organizational structure will be reviewed and amended as required.

Intervention Team

The Intervention Director will lead the intervention team, which will consist of health psychology, health literacy, and physical activity experts who will develop the intervention materials.

Assessment Team

The Assessment Director will lead the assessment team, which will consist of data collection technicians trained by the assessment director and experts in anthropometry, questionnaire administration and health literacy. The assessment team will be responsible for collecting data on the outcome measures at baseline, 4-week, 8-week, 6-month, 12-month, and 24-month visits.

Education Team

The Director of Education and his/her team will be responsible for developing an educational program on PA for the intervention group in the trial.

Timeline

The SAWA trial is designed as an 8-week intervention for rural older adults. The overall duration of the funding period of this study is three years. The following figure shows the timeline of activities (by quarter) for the SAWA trial.

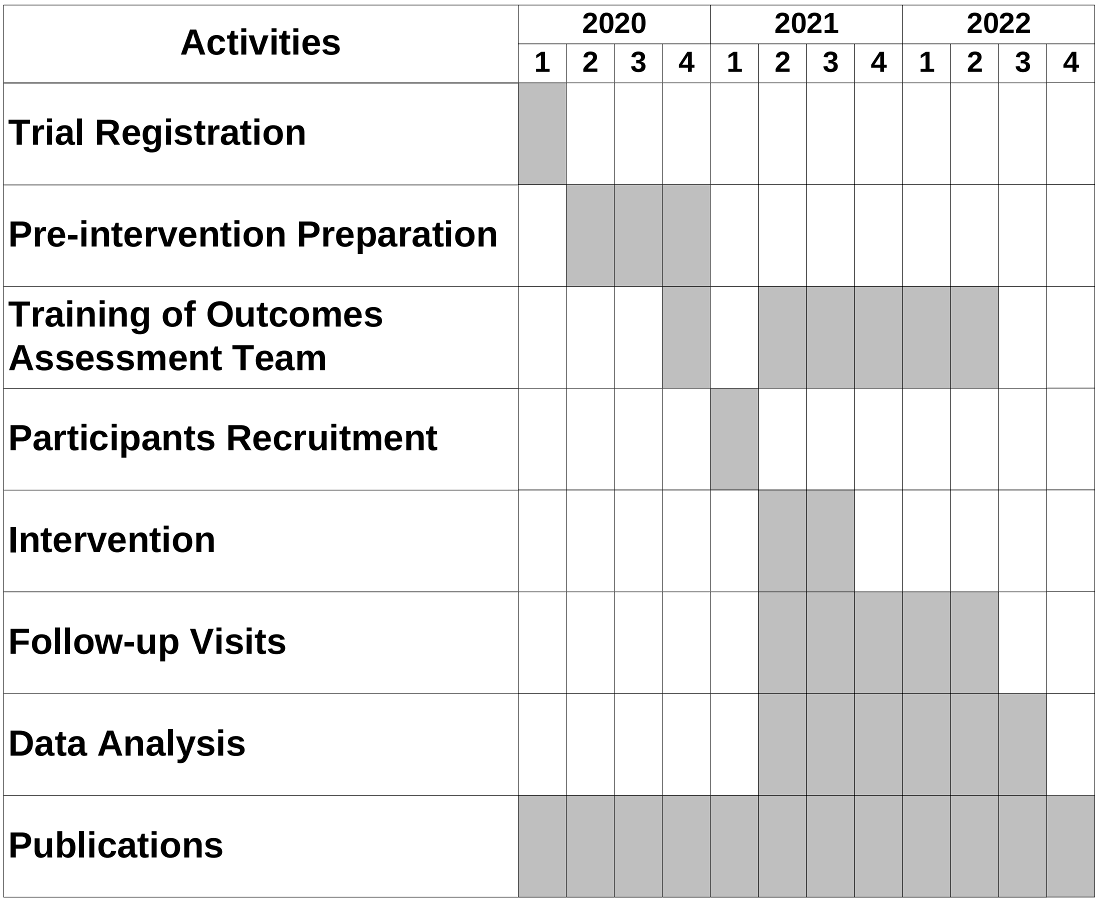

Figure 3. Timeline of Activities (by Quarter) for the SAWA Trial.

Literature Cited

1. Organization WH. Assessing national capacity for the prevention and control of noncommunicable diseases: report of the 2017 global survey. 2018.

2. Ma LW, Yazhe. Wang, Wen. Chen, Weiwei. Interpretation of the report on cardiovascular diseases in China (2017)(in Chinease). *Chin J Cardiovasc Med* 2018; **23**(01): 3-6.

3. Gu J. Interpretation of the report on nutrition and chronic diseases in China (2015)(in Chinease). *Acta Nutrimenta Sinica* 2016; **38**(06): 525-9.

4. Chen W. Report on cardiovascular diseases in China 2015 (in Chinese). *China Medical News* 2016; **0**(12).

5. Liu ML, Yichong. Liu,Shiwei,et al. Burden on blood-pressure-related diseases among the Chinese population,in 2010 (in Chinese). *Chinese Journal of Epidemiology* 2014; (6): 680-3.

6. Moore SC, Lee IM, Weiderpass E, et al. Association of Leisure-Time Physical Activity With Risk of 26 Types of Cancer in 1.44 Million Adults. *JAMA Intern Med* 2016; **176**(6): 816-25.

7. Kyu HH, Bachman VF, Alexander LT, et al. Physical activity and risk of breast cancer, colon cancer, diabetes, ischemic heart disease, and ischemic stroke events: systematic review and dose-response meta-analysis for the Global Burden of Disease Study 2013. *BMJ* 2016; **354**: i3857.

8. Brian W. Ward TCC, Colleen N. Nugent, Jeannine S. Schiller. Early release of selected estimates based on data from the 2015 National Health Interview Survey; 2016. 2016.

9. Zhu W, Chi A, Sun Y. Physical activity among older Chinese adults living in urban and rural areas: A review. *J Sport Health Sci* 2016; **5**(3): 281-6.

10. Wang R, Yan Z, Liang Y, et al. Prevalence and Patterns of Chronic Disease Pairs and Multimorbidity among Older Chinese Adults Living in a Rural Area. *PLoS One* 2015; **10**(9): e0138521.

11. Prince MJ, Wu F, Guo Y, et al. The burden of disease in older people and implications for health policy and practice. *Lancet* 2015; **385**(9967): 549-62.

12. Organization WH. Global recommendations on physical activity for health. 2010. 2015.

13. Wang DN, Morrow-Howell. Wu, Jilei. et al. The Physical Activity Status of Rural Elders and Environmental Factors Analysis in China (in Chinese). *Population and Development* 2017; **23**(03): 76-83.

14. Li S. Status Analysis of Physical Exercise of Agricultural Population in Rural-urban Fringe Zones and It's the Corresponding Strategy (in Chinese). *Journal of Chengdu Sport University* 2014; **40**(10): 52-4.

15. Zhou J, Britigan DH, Rajaram SS, Wang H, Su D. Association between leisure time physical activity preference and behavior: evidence from the China Health & Nutrition Survey, 2004-2011. *BMC Public Health* 2017; **17**(1): 451.

16. Zhou J, Wang H, Rajaram SS, Britigan DH, Su D. Changes in Leisure Time Physical Activity Preferences and Hypertension Risk. *Am J Health Behav* 2019; **43**(1): 145-57.

17. Wang Z, Xu F, Ye Q, et al. Childhood obesity prevention through a community-based cluster randomized controlled physical activity intervention among schools in china: the health legacy project of the 2nd world summer youth olympic Games (YOG-Obesity study). *Int J Obes (Lond)* 2018; **42**(4): 625-33.

18. Chen Y, Ma L, Ma Y, et al. A national school-based health lifestyles interventions among Chinese children and adolescents against obesity: rationale, design and methodology of a randomized controlled trial in China. *BMC Public Health* 2015; **15**: 210.

19. Shumway-Cook A, Brauer S, Woollacott M. Predicting the probability for falls in community-dwelling older adults using the Timed Up & Go Test. *Phys Ther* 2000; **80**(9): 896-903.

20. Greaney ML, Riebe D, Ewing Garber C, et al. Long-term effects of a stage-based intervention for changing exercise intentions and behavior in older adults. *Gerontologist* 2008; **48**(3): 358-67.

21. Harden SM, Johnson SB, Almeida FA, Estabrooks PA. Improving physical activity program adoption using integrated research-practice partnerships: an effectiveness-implementation trial. *Transl Behav Med* 2017; **7**(1): 28-38.

22. Martinson BC, Sherwood NE, Crain AL, et al. Maintaining physical activity among older adults: 24-month outcomes of the Keep Active Minnesota randomized controlled trial. *Prev Med* 2010; **51**(1): 37-44.

23. Glanz K, Rimer BK, Viswanath K. Health behavior: Theory, research, and practice: John Wiley & Sons; 2015.

24. Uchendu C, Windle R, Blake H. Perceived Facilitators and Barriers to Nigerian Nurses' Engagement in Health Promoting Behaviors: A Socio-Ecological Model Approach. *Int J Environ Res Public Health* 2020; **17**(4).

25. Sallis JF, Owen N, Fisher E. Ecological models of health behavior. *Health behavior: Theory, research, and practice* 2015; **5**(43-64).

26. Mehtala MA, Saakslahti AK, Inkinen ME, Poskiparta ME. A socio-ecological

approach to physical activity interventions in childcare: a systematic review. *Int J Behav Nutr Phys Act* 2014; **11**: 22.

27. Soderlund PD. The Social Ecological Model and Physical Activity Interventions for Hispanic Women With Type 2 Diabetes: A Review. *J Transcult Nurs* 2017; **28**(3): 306-14.

28. Rich P, Aarons GA, Takemoto M, et al. Implementation-effectiveness trial of an ecological intervention for physical activity in ethnically diverse low income senior centers. *BMC Public Health* 2017; **18**(1): 29.

29. Rosenberg DE. Outcomes of a multilevel walking intervention for older adults living in retirement communities: University of California, San Diego and San Diego State University; 2010.

30. Bull FC, Al-Ansari SS, Biddle S, et al. World Health Organization 2020 guidelines on physical activity and sedentary behaviour. *Br J Sports Med* 2020; **54**(24): 1451-62.

31. Huston P, McFarlane B. Health benefits of tai chi: What is the evidence? *Can Fam Physician* 2016; **62**(11): 881-90.

32. Lan C, Chen SY, Lai JS. The exercise intensity of Tai Chi Chuan. *Med Sport Sci* 2008; **52**: 12-9.

33. Ngai SP, Cheung RT, Lam PL, Chiu JK, Fung EY. Validation and reliability of the Physical Activity Scale for the Elderly in Chinese population. *J Rehabil Med* 2012; **44**(5): 462-5.

34. Vaughan K, Miller WC. Validity and reliability of the Chinese translation of the Physical Activity Scale for the Elderly (PASE). *Disabil Rehabil* 2013; **35**(3): 191-7.

35. Washburn RA, McAuley E, Katula J, Mihalko SL, Boileau RA. The physical activity scale for the elderly (PASE): evidence for validity. *J Clin Epidemiol* 1999; **52**(7): 643-51.

36. Washburn RA, Smith KW, Jette AM, Janney CA. The Physical Activity Scale for the Elderly (PASE): development and evaluation. *J Clin Epidemiol* 1993; **46**(2): 153-62.

37. Resnick B, Jenkins LS. Testing the reliability and validity of the Self-Efficacy for Exercise scale. *Nurs Res* 2000; **49**(3): 154-9.

38. Umstattd MR, Motl R, Wilcox S, Saunders R, Watford M. Measuring physical activity self-regulation strategies in older adults. *J Phys Act Health* 2009; **6 Suppl 1**: S105-12.

39. Rong H, Lai X, Mahmoudi E, Fang H. Early-Life Exposure to the Chinese Famine and Risk of Cognitive Decline. *J Clin Med* 2019; **8**(4).

40. Xu H, Zhang Z, Li L, Liu J. Early life exposure to China's 1959-61 famine and

midlife cognition. *Int J Epidemiol* 2018; **47**(1): 109-20.

41. Fu C, Li Z, Mao Z. Association between Social Activities and Cognitive Function among the Elderly in China: A Cross-Sectional Study. *Int J Environ Res Public Health* 2018; **15**(2).

42. Li J, Cacchione PZ, Hodgson N, et al. Afternoon Napping and Cognition in Chinese Older Adults: Findings from the China Health and Retirement Longitudinal Study Baseline Assessment. *J Am Geriatr Soc* 2017; **65**(2): 373-80.

43. Buysse DJ, Reynolds CF, 3rd, Monk TH, Berman SR, Kupfer DJ. The Pittsburgh Sleep Quality Index: a new instrument for psychiatric practice and research. *Psychiatry Res* 1989; **28**(2): 193-213.

44. Tsai PS, Wang SY, Wang MY, et al. Psychometric evaluation of the Chinese version of the Pittsburgh Sleep Quality Index (CPSQI) in primary insomnia and control subjects. *Qual Life Res* 2005; **14**(8): 1943-52.

45. Blackford K, Jancey J, Lee AH, et al. A randomised controlled trial of a physical activity and nutrition program targeting middle-aged adults at risk of metabolic syndrome in a disadvantaged rural community. *BMC Public Health* 2015; **15**: 284.

46. Perloff D, Grim C, Flack J, et al. Human blood pressure determination by sphygmomanometry. *Circulation* 1993; **88**(5 Pt 1): 2460-70.

47. Conn VS, Valentine JC, Cooper HM. Interventions to increase physical activity among aging adults: a meta-analysis. *Ann Behav Med* 2002; **24**(3): 190-200.

48. Ribeiro DC, Milosavljevic S, Abbott JH. Sample size estimation for cluster randomized controlled trials. *Musculoskelet Sci Pract* 2018; **34**: 108-11.

Appendices

Appendix A: SAWA Forms and Questionnaires

Appendix B: Intervention Materials

Appendix C: SAWA Recruitment Materials

Appendix A: SAWA Forms and Questionnaires

- **Baseline Demographics, Health Behavior, and Health History Questionnaire**
- **Physical Activity Scale for the Elderly**
- **Sedentary Behavior Measurement Form**
- **Self-Efficacy for Exercise Scale**
- **Physical Activity Self-Regulation Scale**
- **Telephone Interview for Cognitive Status**
- **Pittsburgh Sleep Quality Index**
- **Adverse Event Log**
- **Visit Checklist**

Baseline Demographics, Health Behaviors, and Health History Questionnaire

1. Basic Information

1.1 Participant’s name: _______________

1.2 Village: (1) Guilin; (2) Jianzheng; (3) Qianfeng; (4) Yixue; (5) Xinsheng; (6) Yijia; (7) Huanglian; (8) Tiane

1.3 ID number: _______________

1.4 Investigator 's Signature: _______________

1.5 Date: _______________

2. Socio-demographic Characteristics

2.1 Sex: (1) male; (2) female

2.2 Marital status: (1) married/cohabited; (2) widowed; (3) separated/divorced; (4) never married

2.3 Education level: (1) not formally attended school; (2) primary school drop-outs; (3) primary school; (4) middle school; (5) high school and above

2.4 Employment: (1) yes; (2) no

2.5 Family size: _______________

2.6 Annual household income (RMB): (1) <12000; (2) 12000-19999; (3) 20000-59999; (4) ≥60000

3. Physical Health and Function

3.1 The scale below has numbers from 0 to 100, with 100 representing the best health you can imagine and 0 representing the worst health you can imagine. Your health status today is on the scale of: ______

|-----|-----|-----|------|-----|-----|------|------|-----|-------|

0 100

3.2 Have you ever been diagnosed with any of the following diseases by a doctor at a township/district hospital or above? Please answer in each item and if yes, please provide further explanation.

	Do you have this disease?	Medication usage	Is blood pressure/blood sugar normal?
Hypertension	(1) yes<br>(2) no	(1) take regularly<br>(2) take intermittently<br>(3) never take	(1) yes<br>(2) no<br>(3) I don't know
Diabetes	(1) yes<br>(2) no	(1) take regularly<br>(2) take intermittently<br>(3) never take	(1) yes<br>(2) no<br>(3) I don't know

3.3 Have you been diagnosed with any of the following diseases?

3.3.1 coronary heart disease (1) yes; (2) no

3.3.2 chronic bronchitis/emphysema (1) yes; (2) no

3.3.3 asthma (1) yes; (2) no

3.3.4 psychiatric disorders (1) yes; (2) no

3.3.5 malignant tumors* (1) yes; (2) no

*If you have malignant tumor, please indicate the specific site: ______ (If more than one site, please fill in the first tumor site)

(1) lung; (2) esophagus; (3) stomach; (4) liver; (5) intestines; (6) breast; (7) prostate; (8) cervix; (9) other

4. Daily Behavior

4.1 Smoking status. Please select the option that fits your situation.

(1) never

(2) former (quit smoking for more than six months or more)

(3) current (total smoking to date is over 100 cigarettes)

4.2 Alcohol consumption. How did you drink in the past year, including beer, wine or liquor?

(1) never / seldom; (2) $<$ once a month; (3) $\geq$ once a month

4.3 Daytime napping

4.3.1 I have the habit of taking daytime napping. (1) yes; (2) no (end)

4.3.2 Usually, I take daytime napping ______ day(s) a week.

4.3.3 Usually, I take an average daytime napping of ______ minutes a day.

Physical Activity Scale for the Elderly

1. Leisure-time Physical Activity

1.1 Have you walked outside home in the past week? For example, walking the dog, exercising, and walking to and from work.

(1) never (skip to question 1.1.c); (2) seldom (1-2 days); (3) sometimes (3-4 days); (4) often (5-7 days)

1.1.a How much time do you usually spend doing these things each day?

(1) < 1 hour; (2) 1-2 hours; (3) 2-4 hours; (4) > 4 hours

1.1.b How many kilometers do you usually walk per day?

(1) < 0.5 km; (2) 0.5-1 km; (3) 1-2 km; (4) > 2 km

1.1.c Do you like walking outside the home?

(1) very much like; (2) a little like; (3) average; (4) a little dislike; (5) very much dislike

1.2 In the past week, how did you participate in light sports such as fishing?

(1) never (skip to question 1.2.c); (2) seldom (1-2 days); (3) sometimes (3-4 days); (4) often (5-7 days)

1.2.a What are the specific activities: ______

1.2.b How much time do you usually spend doing these things each day?

(1) < 1 hour; (2) 1-2 hours; (3) 2-4 hours; (4) > 4 hours

1.2.c Do you like light sports?

(1) very much like; (2) a little like; (3) average; (4) a little dislike; (5) very much dislike

1.3 In the past week, how did you participate in moderate sports such as table tennis, dancing, or tai chi?

(1) never (skip to question 1.3.c); (2) seldom (1-2 days); (3) sometimes (3-4 days); (4) often (5-7 days)

1.3.a What are the specific activities: ______

1.3.b How much time do you usually spend doing these things each day?

(1) < 1 hour; (2) 1-2 hours; (3) 2-4 hours; (4) > 4 hours

1.3.c Do you like moderate sport?

(1) very much like; (2) a little like; (3) average; (4) a little dislike; (5) very much dislike

1.4 In the past week, how did you participate in strenuous sports such as jogging, cycling, or swimming?

(1) never (skip to question 1.4.c); (2) seldom (1-2 days); (3) sometimes (3-4 days); (4) often (5-7 days)

1.4.a What are the specific activities: ______

1.4.b How much time do you usually spend doing these things each day?

(1) < 1 hour; (2) 1-2 hours; (3) 2-4 hours; (4) > 4 hours

1.4.c Do you like strenuous sport?

(1) very much like; (2) a little like; (3) average; (4) a little dislike; (5) very much dislike

1.5 In the past week, how did you participate in muscle strength / endurance exercises such as weight lifting, bench press, or push-ups?

(1) never (skip to question 1.5.c); (2) seldom (1-2 days); (3) sometimes (3-4 days); (4) often (5-7 days)

1.5.a What are the specific activities: ______

1.5.b How much time do you usually spend doing these things each day?

(1) < 1 hour; (2) 1-2 hours; (3) 2-4 hours; (4) > 4 hours

1.5.c Do you like muscle strength / endurance exercises?

(1) very much like; (2) a little like; (3) average; (4) a little dislike; (5) very much dislike

2. Household Physical Activity

2.1 In the past week, did you participate in light housework such as washing dishes or sweeping the floor?

(1) no (skip to question 2.2); (2) yes

2.1.a How much time do you spend on light housework for a week: ______ hours

2.2 In the past week, did you participate in heavy housework or chores such as wiping floors, cleaning windows, or moving things?

(1) no (skip to question 2.3); (2) yes

2.2.a How much time do you spend on heavy housework or chores for a week: ______ hours

2.3 In the past week, did you participate in the following activities?

Items	Yes	No
a. Home repairs such as repairing electrical appliances, or doing carpentry	1	2
b. Lawn work or yard care	1	2
c. Outdoor gardening	1	2
d. Caring for another person	1	2

3. Work-related Physical Activity

3.1 In the past week, did you work for pay or as a volunteer?

(1) no (end); (2) yes

3.1.a How much time do you work for pay or as a volunteer for a week: ______ hours

3.1.b Which of the following descriptions best presents your work for pay or as a volunteer

(1) frequent sitting, light upper extremity activity (e.g., office work, ticket takers, etc.)

(2) sitting or standing often, with only a small amount of walking (e.g., cashiers, etc.)

(3) often running, need to carry general heavy objects (such as waiters, letter carriers, etc.)

(4) frequent running, need to carry quite heavy objects (such as construction workers)

Sedentary Behavior Measurement Form

1.1 In the past week, how much time in total did you spend sitting and doing the following intellectual activities during your free time?

1.1.a Play chess (including Chinese chess, go, checkers, etc.) ______ hours ______ minutes

1.1.b Play tiles (mahjong, poker, and solitaire) ______ hours ______ minutes

1.1.c Calligraphy, painting, reading, and writing ______ hours ______ minutes

1.2 In the past week, how much time in total did you spend sitting in your free time (including watching TV, riding in the car, talking, needlework, etc.) ______ hours ______ minutes

Self-Efficacy for Exercise Scale

How confident are you right now that you could exercise three times per week for 20 minutes if:

Item	Not Confident										Very Confident
5.1 the weather was bothering you	0	1	2	3	4	5	6	7	8	9	10
5.2 you were bored by the program or activity	0	1	2	3	4	5	6	7	8	9	10
5.3 you felt pain when exercising	0	1	2	3	4	5	6	7	8	9	10
5.4 you had to exercise alone	0	1	2	3	4	5	6	7	8	9	10
5.5 you did not enjoy it	0	1	2	3	4	5	6	7	8	9	10
5.6 you were too busy with other activities	0	1	2	3	4	5	6	7	8	9	10
5.7 you felt tired	0	1	2	3	4	5	6	7	8	9	10
5.8 you felt stressed	0	1	2	3	4	5	6	7	8	9	10
5.9 you felt depressed	0	1	2	3	4	5	6	7	8	9	10

Physical Activity Self-Regulation Scale

Please comment on the following questions according to your actual situation.

Items	Never	Rarely	Sometimes	Often	Very often
1.1 I mentally kept track of my PA	1	2	3	4	5
1.2 I mentally noted specific things that helped me be active	1	2	3	4	5
1.3 I set short term goals for PA	1	2	3	4	5
1.4 I asked someone for PA advice or demo	1	2	3	4	5
1.5 I asked a PA expert or health professional for PA advice or demo	1	2	3	4	5
1.6 I reminded myself of PA health benefits	1	2	3	4	5
1.7 I mentally scheduled specific times for PA	1	2	3	4	5
1.8 I rearranged my schedule to ensure I had time for PA	1	2	3	4	5
1.9 I purposely planned ways to do PA when on trips away from home	1	2	3	4	5
1.10 I purposely planned ways to do PA in bad weather	1	2	3	4	5

Abbreviations: PA, physical activity; demo, demonstration.

Telephone Interview for Cognitive Status

Now I will ask you a few questions, some of which may be easy for you and some of which may be difficult.

1.1 What is today's year/month/date?

year (1) correct; (2) incorrect

month (1) correct; (2) incorrect

date (1) correct; (2) incorrect

1.2 What is the day of the week? (1) correct; (2) incorrect

1.3 What season are we in? (1) correct; (2) incorrect

1.4 How do you feel about your memory now?

(1) excellent; (2) very good; (3) good; (4) average; (5) bad

1.5 I'm going to read you a list of ten words. Please listen carefully and try to remember them. When I am done, tell me as many words as you can, in any order. Ready?

The words are: hat, car, tree, elephant, cinema, watch, pillow, hospital, pencil, table tennis.

Now tell me all the words you can remember. Answered correctly ______ words.

1.6 We will ask you some subtractions.

(1) One hundred minus 7 equals what? ______

(2) And 7 from that (the answer to (1))? ______

(3) And 7 from that (the answer to (2))? ______

(4) And 7 from that (the answer to (3))? ______

(5) And 7 from that (the answer to (4))? ______

The number of answers correctly _____

1.7 The ten words I read to you earlier, please tell me the words you remember now.

Answered correctly ______ words.

Pittsburgh Sleep Quality Index

	Items	Options			
		(1)	(2)	(3)	(4)
1.1	During the past month, what time have you usually gone to bed at night? Bedtime ______				
1.2	During the past month, how long (in minutes) has it usually taken you to fall asleep each night? Number of minutes ______	≤ 15 min	16~30 min	31~60 min	> 60 min
1.3	During the past month, what time have you usually gotten up in the morning? Getting up time ______				
1.4	During the past month, how many hours of actual sleep did you get at night? (This may be different than the number of hours you spent in bed.) Hours of sleep per night ______				
1.5	During the past month, how often you had trouble sleeping because you…				
	a. Cannot get to sleep within 30 minutes	not during the past month	less than once a week	once or twice a week	three or more times a week
	b. Wake up in the middle of the night or early morning	not during the past month	less than once a week	once or twice a week	three or more times a week
	c. Have to get up to use the bathroom	not during the past month	less than once a week	once or twice a week	three or more times a week

	d. Cannot breathe comfortably	not during the past month	less than once a week	once or twice a week	three or more times a week
	e. Cough or smore loudly	not during the past month	less than once a week	once or twice a week	three or more times a week
	f. Feel too cold	not during the past month	less than once a week	once or twice a week	three or more times a week
	g. Feel too hot	not during the past month	less than once a week	once or twice a week	three or more times a week
	h. Had bad dreams	not during the past month	less than once a week	once or twice a week	three or more times a week
	i. Have pain	not during the past month	less than once a week	once or twice a week	three or more times a week
	j. Other reason(s), please describe ______. How often during the past month have you had trouble sleeping because of this?	not during the past month	less than once a week	once or twice a week	three or more times a week
1.6	During the past month, how would you rate your sleep quality overall?	very good	fairly good	fairly bad	very bad
1.7	During the past month, how often have you taken medicine to	not during the past month	less than once a week	once or twice a week	three or more times a week

	help you sleep (prescribed or "over the counter")?				
1.8	During the past month, how often have you had trouble staying awake while driving, eating meals, or engaging in social activity?	not during the past month	less than once a week	once or twice a week	three or more times a week
1.9	During the past month, how much of a problem has it been for you to keep up enough enthusiasm to get things done?	no problem at all	only a very slight problem	somewhat of a problem	a very big problem

Adverse Event Log

**Participants' ID number**	**Date Reported** (mm/dd/yyyy)	**Adverse Event**	**Start Date/Time** (mm/dd/yyyy 2400 clock)	**Outcome** 1=Resolved 2= Resolved w/sequelae 3=Recovering 4=Unknown 5=Fatal	**End Date/Time** (mm/dd/yyyy 2400 clock)	**Severity** 1= mild 2= mod 3= severe	**Action Taken as a result of Adverse Event** 0= None 1= Refer to Primary Care Physician 2= Other; please explain	**Withdrawal** Did subject withdraw from the study as a result of this AE? Yes/No	**Is Adverse Event serious?** (Determined by the Physician) Yes/No	**Related to Intervention** (Completed by the Physician) 1=Definitely 2=Probably 3=Possibly 4=Unrelated 5=Unknown	**Serious of Adverse Event** (Determined by the Physician) Yes/No	**Unexpected Adverse Event** (Completed by the Physician) Was the AE unexpected in nature, severity, or frequency? Yes/No

Visit Checklist

Village: (1) Guilin; (2) Jianzheng; (3) Qianfeng; (4) Yixue; (5) Xinsheng; (6) Yijia; (7) Huanglian; (8) Tiane

Participant's name: ____________ Sex: Male ☐ Female ☐

Tel: ☐☐☐☐☐☐☐☐☐☐☐

ID number: ☐☐☐☐☐☐☐☐☐☐☐☐☐☐☐☐☐☐

No.	SAWA Assessments	Notes	Investigator's Signature
1	Weight	kg	
2	Percentage Body Fat	%	
3	Visceral Fat		
4	Height	cm	
5	Waist Circumference	cm	
6	Hip Circumference	cm	
7	Blood Pressure (Systolic/Diastolic Pressure)	mmHg	
		mmHg	
		mmHg	
8	Questionnaires	Yes☐ No☐	

Quality Control Staff' Signature____________ Date____________

Appendix B: Intervention Materials

- **Telephone Counseling Techniques**
- **Telephone Counseling Record**

Telephone Counseling Techniques

(1) Telephone Counseling Template for the First Time

a. Telephone counseling process (group leader)

1. First of all, please ask: Hello, is this xxx?

2. Descript the intention of this call: We are the investigators performed physical activity (PA) training and questionnaire survey for you several days ago. Now we need to ask you some questions, can you please make it convenient for us now? (if it's not convenient now, ask the nearest available time)

3. Primary contents

1) Recent exercise situation: What types of exercise were you performing recently, how many times a day, and how many minutes each time for performing PA?

2) Set exercise goals: Your current exercise intensity is (great/okay/not enough), next week you can perform (moderate-intensity PA such as Tai Chi: 2 times a day and 20 minutes for each time or 40 minutes a day; vigorous-intensity PA such as running: 2 times a day and 15 minutes for each time or 20 minutes a day). We will call you every week to see if you have reached your goals.

3) Peer group: We assisted you form a peer group to exercise together after the PA training, do you remember that? You are the leader of group x (e.g., 1) and the group has xx members, including xx, xx, and xx. What kind of exercise do you think is better for your group to perform together? How many times a week can you get together to exercise, and how many minutes for each time? Okay, then your group exercise goal is to xx (e.g., dance five times a week and 40 minutes for each time). Do you think it is okay? Please don't forget to organize your members to exercise together.

b. Telephone counseling process (all intervention subjects)

1. First of all, please ask: Hello, is this xxx?

2. Descript the intention of this call: We are the investigators performed physical activity training and questionnaire survey for you several days ago. Now we need to ask you some questions, can you please make it convenient for us now? (if it’s not convenient now, ask the nearest available time)

3. Primary contents

1) Recent exercise situation: What types of exercise were you performing recently, how many times a day, and how many minutes each time for performing PA?

2) Set exercise goals: Your current exercise intensity is (great/okay/not enough), next week you can perform (moderate-intensity PA such as Tai Chi: 2 times a day and 20 minutes for each time or 40 minutes a day; vigorous-intensity PA such as running: 2 times a day and 15 minutes for each time or 20 minutes a day). We will call you every week to see if you have reached your goals.

3) Peer group: We assisted you to form a peer group to exercise together after the PA training, do you remember that? You are one of the members of group x (e.g., 1), and xx is the leader of your group. The exercise goal of your group is to xx (e.g., dance once a week and 20 minutes for each time). Please don’t forget to exercise with your group.

(2) Weekly Telephone Counseling Template After the First Time

a. Telephone counseling process (group leaders)

1. First of all, please ask: Hello, is this xxx?

2. Descript the intention of this call: We are the investigators performed physical activity training and questionnaire survey for you. Now we need to ask about your exercise situation for the previous week, which is estimated to take 3 minutes, would that be convenient for you? (if it’s not convenient now, ask the nearest available time)

3. Primary contents

1) Personal exercise goal: The exercise goal we set in counseling was xx (e.g., play Tai

Chi twice a day and 20 minutes for each time). Did you reach that goal last week? Do you think the current intensity and amounts of PA are appropriate?

2) Current difficulties: What were the reasons for your failure to reach the goal? Did you encounter any difficulties?

3) Peer group: You are the leader of your peer group, right? Did you organize your members to exercise together regularly? Have you reached the goal of xx (e.g., dancing once a week)?

a. If the goal was not achieved, ask why and record it.

b. If the goal was achieved, know about the positivity of group members, and if they were active, adjust the goal by increasing the amount and intensity of PA appropriately.

b. Telephone counseling process (all intervention subjects)

1. First of all, please ask: Hello, is this xxx?

2. Descript the intention of this call: We are the investigators performed physical activity training and questionnaire survey for you about x (e.g., 2) weeks ago. Now we need to ask about your exercise situation for the previous week, which is estimated to take 3 minutes, would that be convenient for you? (if it’s not convenient now, ask the nearest available time)

3. Primary contents

1) Personal exercise goal: The exercise goal we set in the last counseling was xx (e.g., play Tai Chi twice a day and 20 minutes for each time). Did you reach that goal last week? Do you think the current intensity and amounts of PA are appropriate?

2) Current difficulties: What were the reasons for your failure to reach the goal? Did you encounter any difficulties?

3) Self-efficacy: Which aspects do you think you are doing well in your exercise, could you be more specific? (deliver encouragement). Are there any areas where you are not

doing well? What adjustments can be made in these areas?

4) Peer group: Have you participated in regular peer group activities (e.g., dance) during last week? Did the group leader (xxx) lead you to exercise regularly?

(3) Communication Skills

1. Praising them for doing well in some aspects in exercise. For example, “You exercised x days last week and x hours for each day. You are very active! Keep it up and it will promote your cardiovascular and sleep health (better be tailored, e.g., if you know the participant has sleep problems, you could tell him/her physical activity is good for sleep health)!”, or “You have improved much than before! You spent more time and exercised more often than before!”

2. When participants mention that they exercised less last week for various reasons. We should express understanding and try to attribute the reason to external factors which are relatively easy to change. For example:

1) “It did rain almost every day last week and it was not convenient to exercise outside. But you can perform exercise at home on rainy days in the future so that keep your body moving and healthy.”

2) “It is really important to spend more time to company your granddaughter. How old is your granddaughter? You can also take her walk around these days, and exercise together.”

3) Encourage them to readjust their goals according to their situation and ask about whether they reach that goal at the next counseling.

Telephone Counseling Record

Basic Information			Telephone Counseling Record at 1 Week							
			Personal Goals		Group Goals					
Group Number	Participants' Name	Telephone Number	Types of Exercise	Exercise Times/Week	Types of Exercise	Group Exercise (Times/Week)	Time for Per Session (min)	Special Cases at 1 Week	Counseling Date	Investigator's Name

Basic Information			Telephone Counseling Record at X Weeks (After 1 Week)														
			Personal Goals							Group Goals							
						Adjust Personal Goals		Group Exercise		Group Exercise		Adjust Group Goals					
Group Number	Participants' Name	Telephone Number	Whether the Personal Goals were Reached Last Week	Reasons for Did Not Reach the Personal Goals	Measures to Assistant	Types of Exercise	Exercise Times/Week	Whether Absent from Group Exercise	Reasons for Absence	Whether the Group Goals were Reached Last Week	Reasons for Did Not Reach the Group Goals	Types of Exercise	Group Exercise (Times/Week)	Time for Per Session (min)	Special Cases at X Weeks	Counseling Date	Investigator's Name

Appendix C: SAWA Recruitment Materials

Individuals with 60 years of age or older were invited to participate.

Exclusion criteria: people screened for eligibility who meet any one of the following criteria cannot be our participants.

1) have a history of stroke, arthritis, Parkinson's disease, severe pneumonia or severe heart disease;

2) have severe cognitive or hearing disorders;

3) had a major operation in the last 3 years;

4) poor control of hypertension or diabetes;

5) being treated for cancer;

6) have fallen during the last 12 months.

Inclusion criteria: people screened for eligibility who meet the following 4 items can be our participants.

1) be able to answer the phone;

2) be able to cover 400 meters within 15 minutes;

3) be able to walk without assistance or crutches;

4) be able to complete the Timed Up & Go test (the process is shown below).

When I send out the command "Go", please

a. Get up from your chair;
b. Walk 3 meters in a straight line at your usual speed, and you can use a cane;
c. Turn around after walking 3 meters, then continue to walk 3 meters in a straight line at your usual speed and return to the chair;

Note: The timing method starts from [giving instructions] and ends at [sitting on the chair again], and compares with 12s.

Stay Active While Aging

(SAWA)Trial

PROTOCOL

Final Version

TABLE OF CONTENTS

Specific Aims 63

Background and Significance 64

Background 64

Significance 65

Overview of Trial Design 67

Design 67

Randomization 68

Eligible Criteria 69

Inclusion Criteria 69

Exclusion Criteria 69

Interventions 70

Data Collection 74

Quantitative surveys 74

Qualitative Interviews 80

Outcomes 80

Quality Assurance and Control 81

Data Management and monitoring 82

Sample Size Computation 83

Data Analysis Plan 84

1. Primary Analyses 84

2. Secondary Analyses 84

3. Subgroup Analyses 85

Trial Organization 87

Timeline 87

Literature Cited 88

Appendices 92

Appendix A: SAWA Forms and Questionnaires 93

Appendix B: Intervention Materials 112

Appendix C: SAWA Recruitment Materials 119

Specific Aims

The study aims to explore the factors affecting the physical activity of rural older adults, and to develop a suitable PA intervention model for rural older adults based on a socio-ecological model to provide valuable information for future study designs. The specific aims are as follows:

(1) Creating and implementing a PA intervention model suitable for rural older adults.

(2) Evaluate the effect and sustainability of the PA intervention model to provide valuable information for future study designs and yield evidence to inform the decision-making of health policy.

Background and Significance

Background

(1) Improving the PA Level of Residents has Far-reaching Implications for National Chronic Disease Prevention and Control

In recent decades, chronic diseases have shown a high prevalence in most of the world's population, and their incidence is increasing every year.[1] Taking hypertension as an example, the prevalence of hypertension in China was 25.2% in 2012, 5 times (5.0%) that of 1959 and 1.33 times (19.0%) that of 2002, and the number of deaths due to hypertension accounted for 24.60% of all deaths in 2010,[2,3] with related health costs accounting for 6.61% of the direct economic burden.[4,5] Therefore, the burden of chronic diseases in China is increasingly serious, and the prevention and control work is very serious.

Numerous studies have shown that regular PA can significantly reduce the incidence of many chronic diseases.[6,7] For example, PA can reduce the risk of developing up to 26 types of cancer.[6] However, the PA of the Chinese population is not promising. In 2013, the regular PA rate of the adult population in China was only 18.7% (22.2% in urban areas and 14.3% in rural areas), compared to about 50% in the United States during the same period.[8] In addition, according to the China Health& Nutrition Survey, PA (including work-related PA and leisure-time PA) decreased by nearly one-third for adult males and 42% for females from 1991 to 2011.[9]

Therefore, increasing the PA level of the Chinese population has profound implications for chronic disease prevention and control.

(2) Conducting PA Interventions in Rural Older Adults is Critical

The Chinese rural older adults have a huge burden of chronic diseases. In the case of hypertension, previous studies indicated that the prevalence of hypertension in China's rural older population is as high as 76.4%[10] and is extremely poorly controlled.[11] In

addition, the prevalence of comorbidity with two or more chronic diseases exceeds 90%.[10] The high prevalence of chronic diseases also brings with it a huge health care burden.

In order to reduce the burden of chronic diseases, the Word health Organization (WHO) recommends that older adults should remain physically active.[12] However, the level of PA among the Chinese rural older population is worrying. As a result of changes in productive lifestyle, their PA has declined significantly. Firstly, as mechanization increases and older people age, the work-related PA level of older rural residents is rapidly diminishing.[13] Furthermore, the level of PA in leisure time of rural older adults has not increased despite the increase in leisure time,[9,13]and the popularity of entertainment activities such as television and mahjong has prevented many rural older adults from participating in PA.[13,14] Previous studies have shown that overall levels of leisure-time PA preferences and behaviors among older people in rural China are low,[15,16] and there is no evidence that this situation is being alleviated.

However, current studies on PA interventions focused on other populations,[17,18] with few studies concerning rural older adults in China. This suggests that research in this area is lagging.

Therefore, it is necessary to carry out studies on PA interventions for rural older adults, to prevent, control, or delay the onset of many chronic diseases, reduce the health economic burden, and inform future similar studies.

Significance

The SAWA study is a sustainable, pragmatic PA intervention in Chinese rural older adults. As described above, the Chinese rural older adults have a huge burden of chronic diseases, however, the PA level among the Chinese rural older population is worrying. Thus, there is an urgent need to develop and test the PA intervention strategies in rural older adults in China.

The SAWA study seeks to use the socio-ecological model to guide an intervention

to increase PA in rural older populations. It proposes to conceptualize an intervention model by integrating the socio-ecological model with constructs from Health Belief Model, Social Cognitive Theory, and Community Organization and Community Building theories, in the light of impact factors of PA behavior. It aims to change participants' PA at the individual level, interpersonal level, and community level. Telephone counseling, printed materials, training sessions, peer group, group sharing, and coaching will be provided and established as intervention measures. A comprehensive evaluation of the effectiveness of the intervention program will be carried out in order to create an effective PA intervention model for rural older adults in China.

If the intervention model is applied to other populations, it will help to increase the PA level of the populations, thereby reducing or delaying the onset of various chronic diseases, reducing the burden of diseases on society and families, and improving the quality of life. The study will also yield valuable information on the efficacy and effectiveness of PA across a broad spectrum of important health outcomes. The study will influence both clinical practice and public health policy, and will, therefore, benefit individuals and society.

Overview of Trial Design

Design

This study is a single-blinded cluster randomized controlled trial to test the effect and sustainability of the multilevel PA intervention to improve PA levels in rural older adults. There were 8 villages participating in total: 4 intervention sites and 4 control sites. RE-AIM (Research, Efficacy, Adoption, Implementation, and Maintenance) will be applied to evaluate the pre-post and intervention-control differences as well as external validity.

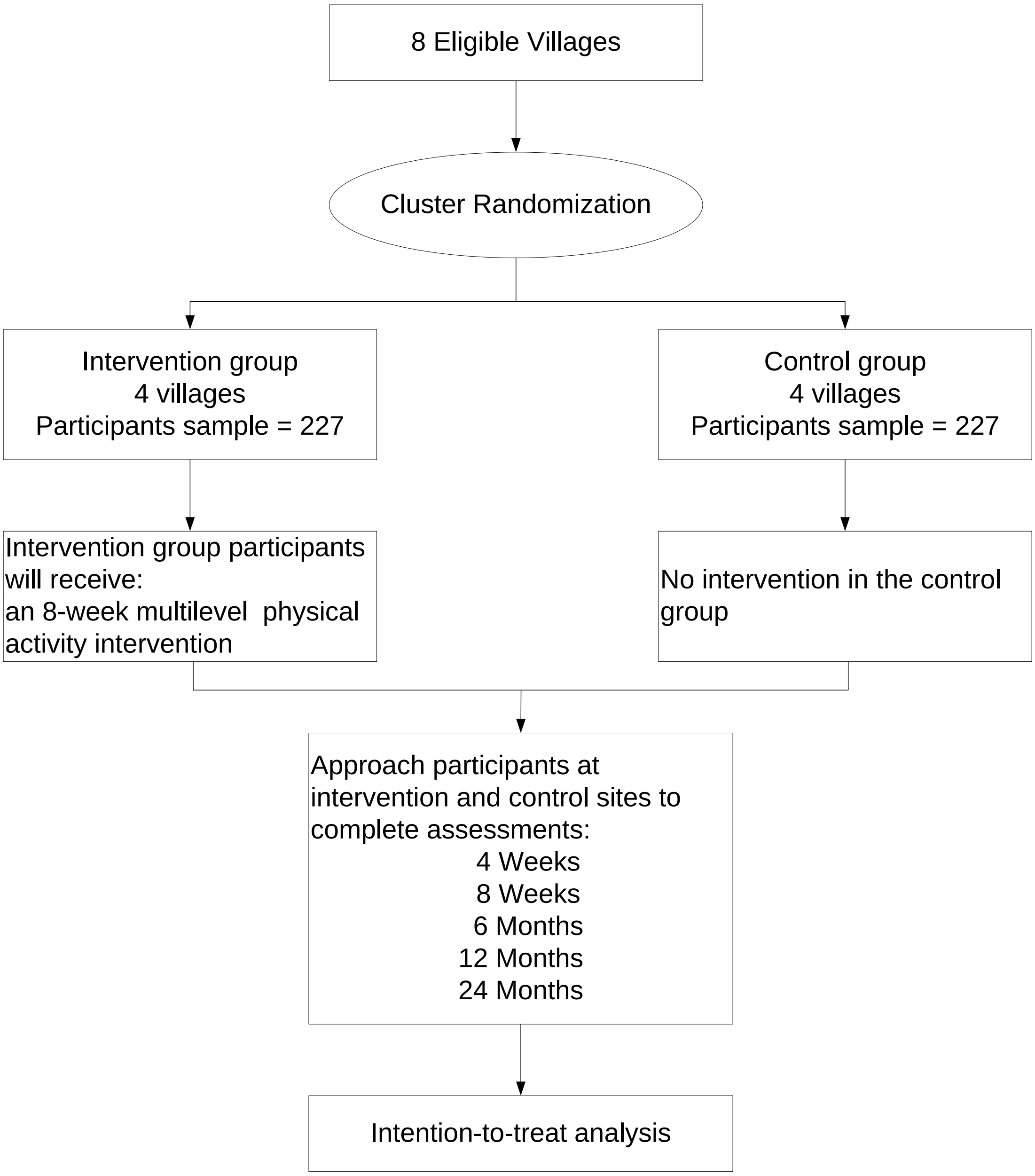

Figure 1. Overview of SAWA Intervention

Randomization

Multistage random sampling was used, and the random number was generated by the computer program (www.random.org). First, Chengdu was randomly selected from 18 cities in Sichuan Province. Second, Jianyang was selected out of 20 regions in Chengdu. Third, eight villages were randomly selected (Guilin, Jianzheng, Qianfeng, Yixue, Xinsheng, Yijia, Huanglian, Tiane), and these villages were further randomly assigned to either the intervention group or the control group (four villages for each group). There is a separation distance (minimum of 4 km) between each of these villages to minimize the risk of contamination.

Eligible Criteria

Inclusion Criteria

Participants are eligible to be included in the trial only if all of the following criteria apply:

(1) Be 60 years of age or older and

(2) Be able to answer phone calls and

(3) Be able to walk 400 m in 15 min and

(4) Be able to walk without the help of others or crutches and

(5) Be able to complete the Timed Up & Go test.[19]

Exclusion Criteria

Participants are excluded from the trial if any of the following criteria apply:

(1) Have a history of stroke, arthritis, Parkinson's disease, severe pneumonia or severe heart disease or

(2) Have severe cognitive or hearing impairment or

(3) Had major surgery in the past 3 years or

(4) Poor control of hypertension or diabetes or

(5) Be receiving cancer treatment or

(6) Have fallen in the past year.

Interventions

In light of current evidence on PA intervention and follow-up periods,[20-22] our project will consist of an 8-week intervention and a 24-month follow-up. The intervention group will receive the intervention from May to July 2021. Figure 2 shows the timeline of the 8-week intervention strategies.

Level	Intervention strategies	1 wk	2 wk	3 wk	4 wk	5 wk	6 wk	7 wk	8 wk
Individual level	Telephone counseling	×	×	×	×	×	×	×	×
	Printed materials	×	×	×	×	×	×	×	×
	Training sessions	×			×				×
Interpersonal level	Peer group	×	×	×	×	×	×	×	×
Community level	Group sharing	×			×				×
	Coaching	×			×				×

Figure 2. Timeline of the 8-week Intervention Strategies

The underlying theoretical model used to motivate the participants is derived from the socio-ecological model (SEM).[23] The SEM consists of five dimensions: individual, interpersonal, community, organizational, and public policy levels, which are thought to be the determinants of health-related behavior.[24,25] It emphasize the dynamic interaction among biological, psychological, behavioral, social, and environmental factors. Systematic reviews from intervention studies based on the SEM have revealed the effectiveness of such interventions, indicating that not only PA level was increased, but sedentary time was reduced.[26,27] For the current study, a condensed version of the SEM consisting of 3 levels of influence were utilized: individual, interpersonal, and community levels.[28,29] Our detailed interventions based on the three levels (individual, interpersonal, and community levels) are as follows:

1. Individual Level

On the individual level, telephone counseling, printed material, training sessions, and

self-monitoring will be employed to improve individual factors, such as knowledge, beliefs, perceived barriers and benefits, self-regulation, self-efficacy, and skills. Specifically, changes in perceived barriers, self-regulation, and self-efficacy will be achieved by telephone counseling; all the information printed in the materials will lead to improvements in knowledge, perceived benefits, and beliefs towards PA. The skills of PA will be improved through training sessions. Self-regulation and self-efficacy will also be achieved by self-monitoring.

Telephone Counseling

The participants will receive the feedback on their PA and health behaviors in relation to the recommendations through the telephone counseling. This feedback will also highlight the discrepancy between their heath and PA goals. The telephone call will be used to encourage participants to exercise and provide individualized assistance as follows:1) set the exercise goals based on their preference and the intensity of their daily exercise, 2) participants will be asked "did you achieve the exercise goals last week?", 3) investigators will praise the respondents who have achieved the goals, and adjust them according to the global recommendations[30] on PA for older adults, 4) participants who did not achieve the goals will be encouraged, and investigators will assist them to solve the difficulties.

Printed Materials

Each participant in the intervention group will receive a booklet, which allows individuals to study information on their own and as need. Information provided in the printed materials include the potential risks of PA, the benefits of PA, the PA recommendation, local exercise resources for PA, the feasible PA for older adults, and safety tips for performing PA.

Training Sessions

The training sessions will be taught by sports experts who are certified to engage in physical education. Stretching exercises and Tai Chi will be mainly included in the

courses. Stretching exercise was a complement to other exercises. Tai Chi is a moderate-intensity exercise that has been supported in the literature for improving cognitive function and preventing clinical diseases, such as Parkinson’s disease, osteoarthritis, and so on[31,32]

2. Interpersonal Level

On the interpersonal level, peer groups will be organized to improve collective efficacy, observational learning, and incentive motivation. Collective efficacy will be enhanced by sharing group goals and communicating with each other. Each participant will learn new information and behaviors from other teammates by observing the behaviors, which will receive positive reinforcement. Further, the well-performing teammates and responsible leaders (encouraging teammates to participate in the group activities) will be rewarded to increase their motivation to participate in PA.

Peer Group

Peer groups will be established based on the wishes of participants. Each group will involve 3-10 members. Investigators will assist groups to set the group goals based on their common preferences. The group leader nominated by the group is responsible for reminding their members to exercise and organizing group activities. In order to enhance collective efficacy, observational learning, and incentive motivation, the members can communicate and learn from each other through the interactions. The peer groups will receive telephone calls once a week in the intervention period as well, and will be reminded to achieve the group goals by telephone. The well-performing teammates and responsible leaders will be rewarded.

3. Community Level

On the community level, group sharing and coaching will be formed to improve social capital and operationalize environmental factors. Participants will be encouraged to join the group sharing led by investigators, through which they can share their experiences and help each other in order to solve problems. Environmental factors will be

operationalized through the identification of harmful and beneficial factors in the environment and utilization of environmental resources with the help of coaches.

Group Sharing

Both the participants and investigators will join the group sharing. The group sharing will be led by the education team to discuss weekly topics, share stories with others in the group, and to engage in problem-solving together. The purpose of group sharing is to provide participants with opportunities to share their own exercise experiences with others, promote mutual trust, and increase social capital. For example, the participant who achieves the goals weekly will be asked "How did you keep exercising when the weather was terrible or the farming was busy?" Furthermore, participants will be encouraged to use a variety of supports including family and friends, as well as neighborhood and community supports.

Coaching

The participants will be guided by coaches to identify barriers to PA in rural settings and factors in the environment that are conducive to exercise. Coaches will also provide guidance on how to comprehensively utilize the environmental resources (e.g., walking paths, open spaces for PA) based on identified facilitating and constraining factors.

Data Collection

All the outcomes will be collected at baseline, 4 weeks, 8 weeks, 6 months, 12 months, and 24 months after the baseline. We will obtain written or thumbprint informed consent from each participant prior to the beginning of each interview. Both quantitative and qualitive data will be involved in the survey. The quantitative data will be obtained by the questionnaires or the anthropometry of each participant measured by trained technicians. The qualitive data will be obtained by the one-to-one interviews.

Quantitative surveys

The following data will be collected based on the questionnaires by trained investigators.

Physical Activity

The PA level of participants including leisure-time activity, household activity, work-related activity, and total PA will be measured by the Physical Activity Scale for the Elderly (PASE), a widely used instrument for older adults. Previous studies demonstrated the validity and reliability of the scale for assessing the PA level of older adults in the Chinese population.[33,34] The score of each item of the PASE is calculated by multiplying the weight and frequency.[35,36] The total PA score is the sum score of the leisure-time activity, household activity, and work-related activity. The primary outcome is the changes in leisure-time activity at 8 weeks, and the other two domains of PA and total PA are the secondary outcomes.

Proportions of meeting WHO recommendations

The WHO recommends that adults should do at least 150-300 minutes of moderate-intensity sport, or at least 75-150 minutes of vigorous-intensity sport, or an equivalent combination of moderate- and vigorous-intensity activity throughout the week. Participants were defined to “meet the WHO physical activity recommendation” if they spend 0.357h/day or more time on moderate-intensity sport, or 0.179h/day or more.

on the vigorous-intensity sport, or an equivalent combination of moderate- and vigorous-intensity activity. The hours per day participants spent on moderate- and vigorous-intensity sport will be obtained by converting the frequency (days/week) and duration (hours/day) based on the Hours Per Day Conversion table.[37]

Leisure-time Activity Preferences

Participants will be asked how much they like each of the following three intensity activities: light-intensity sport, moderate-intensity sport, vigorous-intensity sport, and muscle strength sport. The preferences for each type of activity was related on the scale from 1 to 5 (1 = strongly dislike, 2 = dislike somewhat, 3 = neutral, 4 = like somewhat, and 5 = strongly like).

Sedentary Behavior

Self-reported sedentary behavior will be assessed through the question, "In the past week, how much time did you spend in total on sitting during your leisure time?" Participants will be asked to further indicate the sedentary time for each behavior (playing cards, playing chess, reading, writing, socializing with friends or family, doing hobbies, driving, riding, time on public transport, and any other activities). When two or more activities are carried out at the same time, only the time for the main activity should be counted. For example, if you are watching TV and doing crafts, then count it as TV time or craft time, but not both.

Self-efficacy

Self-efficacy will be obtained through the Self-Efficacy for Exercise Scale (SEE), which is suitable for older adults and has been tested the validity and reliability by a previous study.[38] Participants were asked to answer the questions in 9 different conditions, for example, "How confident are you that you could exercise three times per week and 20 minutes per time if the weather is not good?" The SEE score, ranging from 0-90, is scored by adding the score on each item (0-10), with higher scores indicating higher confidence levels on regular exercise.

Self-regulation

The 12-item Physical Activity Self-Regulation Scale (PASR-12) which is concise and validated for older adults will be used to assess self-regulation.[39] It comprises 12 items addressing the self-regulatory strategies, involving the following dimensions: self-monitoring, goal setting, eliciting social support, reinforcements, time management, and relapse prevention, using a 5-point response scale ("never = 1", "rarely = 2", "sometimes = 3", "often = 4", "very often = 5"). Each item has a score ranging from 1 to 5 and the PASR-12 can have a score ranging from 12 to 60.

Self-rated health

Self-rated health will be obtained by the EQ-5D visual analog scale, which is the part of the EQ-5D-5L. The participants will be asked "how good or bad your health is today"? The scale records the participants' self-rated health status on a graduated (0-100) scale. The zero and 100 means the worst health and the best health, respectively. The self-rated health can be used as a quantitative measure of health outcome that reflect the participants own judgement.

Cognitive Function

Consistent with prior China Health and Retirement Longitudinal Study publications,[40,41] we will use the Telephone Interview for Cognitive Status (TICS-10), a questionnaire that assesses the individual's orientation, attention, and episodic memory.[42,43] Participants will be asked to reply the date (year, month, day), the day of the week, and season, serial subtractions of 7 from 100 five times, and immediate and delayed recall a list of Chinese nouns as many as they can. The orientation will be assessed by replying to the date, the day of the week, and the season, while the attention will be assessed by computing serial subtractions of 7 from 100 five times. Episodic memory will be assessed by immediate and delayed Chinese nouns recall. Immediate recall refers to asking the participants to immediately recall as many nouns as they could after investigators read a list of 10 Chinese nouns. Delayed recall refers to asking

the participants to recall as many of the original nouns as possible after a few minutes. TICS-10 scores will be calculated by summing the three measures above, and the possible total scores range from 0 to 5 for orientation, 0 to 5 for attention, and 0 to 10 for episodic memory with higher scores indicating better cognitive function.

Night-time Sleep Quality

The night-time sleep quality will be measured by the Pittsburgh Sleep Quality Index (PSQI).[44] The PSQI has been translated and adapted to Chinese populations, and has shown the validity and reliability in a previous study.[45] The PSQI is comprised of 7 dimensions (subjective sleep quality, sleep latency, sleep duration, habitual sleep efficiency, sleep disturbances, use of sleeping drugs, and daytime dysfunction). Participants will provide subjective sleep estimated based on the previous month. The answers will be used to generate scores for each of the 7 subcomponents, which ranged from 0 to 3. Overall sleep quality scores will be calculated as the sum of these factors (0-21), with a higher score indicating poorer sleep quality. A PSQI global score more than 5 is indicative a poor sleep quality.

The following outcomes will be based on the anthropometry of each participant measured by trained technicians.

Weight

Weight will be measured to the nearest 0.1 kg with the participants in light clothing and bare feet using Tanita BC-601 electronic scale.

Percentage Weight Change

Percentage weight change will be calculated as the percent of differences between each participant's measured weight at follow-up stages and at baseline.

Body Mass Index

Body mass index (BMI) is an individual's weight in kilograms divided by the square of height in meters. Height will be measured to the nearest 0.1cm with the participants in

bare feet, back against the wall, heels together, and eyes looking straight ahead using portable stadiometers. According to BMI classification of the Chinese reference, participants can be categorized into four groups as following: underweight (BMI < 18.5 kg/m^2), normal weight (18.5 $kg/m^2 \leq$ BMI < 24.0 kg/m^2), overweight (24.0 $kg/m^2 \leq$ BMI < 28.0 kg/m^2), and obesity (BMI $\geq$ 28.0 kg/m^2).

Percentage Body Fat and Visceral Fat

Percentage body fat and visceral fat will be measured to the nearest 0.1% and 1, respectively, using Tanita BC-601 analyzer scales, with the participants wearing no shoes and socks. Participants will stand on a platform scale including electrodes, enabling the electric current to pass from one foot to the other. Through this process impedance is measured and the body fat percentage is calculated using a pre-established body fat predictive algorithm in the device, taking into account the age, gender, weight, height, and level of PA.

Waist Circumference

Waist circumference will be measured at the midpoint of the lowest rib margin and the upper margin of the iliac crest in the standing position[46]. The tape should be in contact with the skin but not compress soft tissue and twists in the tape should be avoided. The participants should be required to breathe normally. The same measuring tape should be used throughout the trial. Waist circumference will be recorded to the nearest 0.1 cm. According to the Chinese reference, abdominal obesity will be defined by waist circumference of at least 90 cm for men and 85 cm for women.

Hip Circumference

Hip circumference will be measured at the largest level of the symphysis pubis and gluteus maximus[46]. The tape should be in contact with the skin but not compress soft tissue, and twists in the tape should be avoided. The participants should be required to breathe normally. The same measuring tape should be used throughout the trial. Hip circumference will be recorded to the nearest 0.1 cm.

Waist-to-hip Ratio

We will obtain the waist-to-hip ratio through waist circumference divided by hip circumference.

Waist-to-height Ratio

We will obtain the waist-to-height ratio through waist circumference divided by height.

Systolic and Diastolic Blood Pressure

Blood pressure measurements were performed with a calibrated Omron U30 electronic sphygmomanometers in the sitting position. Strictly following the America Heart Association's Standardized protocol,[47] measurements should be taken with an interval of minimum 5 minutes of the rest for the participants in a quiet setting without distractions. The second two readings will be averaged to be recorded as the systolic or diastolic blood pressure[48].

Demographic Information and Health Related Questions

For descriptive purposes, the following participant socio-demographic characteristics, including sex (male/female), age, education level (illiterate, elementary, middle school, high school or above), marital status (married, never married, widowed, divorced), household income (<12000 RMB, 12000-19999 RMB, 20000-59999 RMB, ≥ 60000 RMB), and employment (yes/no) will be collected at baseline.

Health behaviors including smoking status (never, former, current), alcohol consumption (never/seldom, < once a month, ≥ once a month, daytime napping (0, 1-60 minutes/day, over 60 minutes/day) will also be obtained at baseline.

Self-reported medical history, including hypertension, diabetes mellitus, asthma, coronary heart disease, chronic bronchitis/emphysema, psychiatric disorders, and malignant tumors will be collected at baseline.

Adverse Events

A surveillance form will be used to screen for adverse events (AEs) and serious adverse events at each assessment visit (see Appendix A). Serious, unexpected AEs that are related or are possibly related to the study will be reported to the Institutional Review Board (IRB) immediately.

Qualitative Interviews

We will obtain oral consent from each participant prior to the beginning of each interview. All interview will be recorded and transcribed. We will use RE-AIM to identify the domains, including: (1)Reach, participant perception of the program recruitment process; (2)Efficacy, is the intervention benefits for improving health benefits, the impact of intervention on participants; (3)Adoption, participants' evaluation of the adoption of each intervention; (4)Implementation, participants' satisfaction with the intervention program as a whole; (5)Maintenance, participants' willingness to continue performing PA after the end of the intervention and their overall attitude toward PA before and after the intervention.

Outcomes

Our primary outcome is the changes in leisure-time activity at 8 weeks. Our secondary outcomes include household activity, work-related activity, total PA, proportions of meeting WHO recommendations, leisure-time preferences, sedentary behavior, self-efficacy, self-regulation, self-rated health, cognitive function, night-time sleep quality, weight, percentage weight change, body mass index, percentage body fat, visceral fat, waist circumference, hip circumference, waist-to-hip ratio, waist-to-height ratio, and systolic and diastolic blood pressure at all assessment time, as well as the leisure-time activity at 4 weeks, 6 months, 12 months, and 24 months.

Quality Assurance and Control

Quality assurance and quality control are of paramount importance in a randomized controlled trial. Standardized protocols for all measurements have been developed, and adherence to the written protocols is of utmost importance. All data collection personnel will be certified as competent to make the required measurements by trained experts. The following is a summary of the quality assurance and control program:

1. Personnel

Quality control of personnel is carried out in three main areas: a) Staff in surveys, interviews, and telephone counseling will be trained by professionals, with unified technical standards. b) Clear division of jobs, e.g. logistics during the investigation and coordination of personnel on site will be handled by different people. c) Responsibilities will be clearly defined, e.g. a person will be responsible for the safekeeping and storage of questionnaires.

2. Research Instruments

a) The study proposes to use internationally recognized research methods and tools (such as the PASE) to ensure the quality of the data. b) Intervention materials (e.g. dissemination materials) are developed by a team member specializing in physical education and sport, in consultation with relevant experts and with reference to national and international standards, in order to ensure the correctness and traceability of knowledge.

3. Quality Management System

a) Develop and implement a quality control program within the project team to ensure quality control at each key stage. b) Establish good external communication and feedback mechanisms with the team leaders of the peer groups and key figures of the villages to implement quality control.

Data Management and monitoring

Face-to-face interviews will be conducted to collect data by eligible assessment team at baseline, 4 weeks, 8 weeks, 6 months, 12 months, and 24 months after the baseline. The collected data will be transferred from paper form to electronic record through data entry by study personnel. Standardized approaches will be used to ensure the high-quality of data. Selected project managers will build the electronic database. Confidentiality of the database will be protected by using password-protected computers. Only those designated by the project manager can access the data using the encrypted computers under the supervision of management. The staff are trained to comply with and use protected health information.

The study team will conduct monthly quality monitoring checks. The database will only be shared among the study team. Emails will be encrypted if data will be sent between the project staff, however, emails will be minimized.

Sample Size Computation

Based on the prior PA intervention meta-analysis, the sample size required for each group was estimated to be about 90 with a power of 80%, an α of 0.05,and assumed an effect size of 0.42.[49] Because the randomization occurs at the village level, we need to consider the clustering effect.[50]

The sample size (SS) for a clustered RCT is defined by equation (1):

$$SS_{\text{cluster RCT}}=SS_{\text{standard RCT}}\times DE \quad (1)$$

The design effect (DE) is obtained from equation (2):

$$DE=1+(n-1)\times ICC \quad (2)$$

$$\text{Where: } ICC=\frac{\sigma^2_{between}}{\sigma^2_{between}+\sigma^2_{within}} \quad (3)$$

and n=cluster size (number of participants per cluster)

In equation (3), $\sigma^2_{between}$ is the between-cluster variance for the outcome measure and σ^2_{within} is the within-cluster variance for the outcome measure.

We conducted a pilot study prior to the SAWA to calculate the DE. With a DE of 2.1, we will need a sample size of 189 (90×2.1) for each group after factoring in the cluster effects. Taking participation attrition into account, we will screen 20% more participants. Each group will have 227 participants, so the total sample size will be 454 participants for the two groups (intervention group and control group).

Data Analysis Plan

1. Primary Analyses

The primary aim will be to assess the leisure-time activity level at 8 weeks in the context of linear mixed model effects multilevel models. In this analysis, the intention-to-treat approach will be used. In addition to the intervention group, assessment time, and their interaction terms, the model may include individual-level (age, sex) variables as explanatory covariates. A random village intercept term is included to account for clustering by villages. The primary study hypothesis of SAWA will be tested based on a two-tailed significance level of 0.05.

An intention-to-treat analysis that includes all randomized participants, regardless of the number of assessments obtained, will be conducted. The analytical plans are flexible and will be adapted as scientific perspectives are advanced.

2. Secondary Analyses

a. Handling of missing data

Although substantial effort will be employed to minimize missing data, it is inevitable that some missing data will occur. Missing data are expected due to drop outs and missed visits. The leisure-time activity level is a subject-level variable and missing assessments would influence tests relative to treatment-by-time interactions. The primary analysis described above will be conducted within each dataset using linear mixed effect models and employ restricted maximum likelihood using all available data. Results of datasets will be combined to produce final estimates for between-group comparisons. This approach assumes data are missing at random: the mechanism that gives rise to missing data relies on observed data only. To assess the sensitivity and robustness of the results, we will compare results of two sets of analyses: i) all participants, with or without missing data, were subjected to the repeated measures linear mixed model analysis; ii) we will perform the linear mixed model with missing

data imputed using multiple imputation.

b. Secondary Outcomes

All the data will be collected at the baseline, 4 weeks, 8 weeks, 6 months, 12 months, and 24 months. The secondary outcomes include household activity, work-related activity, total PA, proportions of meeting WHO recommendations, leisure-time preferences, sedentary behavior, self-efficacy, self-regulation, self-rated health, cognitive function, night-time sleep quality, weight, percentage weight change, body mass index, percentage body fat, visceral fat, waist circumference, hip circumference, waist-to-hip ratio, waist-to-height ratio, and systolic and diastolic blood pressure at all assessment time, as well as the leisure-time activity at 4 weeks, 6 months, 12 months, and 24 months. Generalized linear mixed models were used to test the effect of the intervention on the binary outcomes. For continuous outcomes, analyses will be performed using a similar strategy as the primary analyses.

3. Subgroup Analyses

Modification of the effect of intervention upon primary and secondary outcomes will be performed separately in subgroups including age groups, sex, education level, household income, and BMI at baseline. Tests for key interactions (different intervention effects between sub-groups) will also be performed. However, it is recognized that the study may not be powered adequately to detect interactions; all subgroup analyses will be considered exploratory.

Trial Organization

The Principal Investigator will oversee and monitor progress to reach the milestones with decisions concerning short-term goals and the evaluation of longer-term progress being discussed with the research staff via weekly meetings. The research team will be organized into sub-teams of investigators and staff. The overall organizational structure will be reviewed and amended as required.

Intervention Team

The Intervention Director will lead the intervention team, which will consist of health psychology, health literacy, and physical activity experts who will develop the intervention materials.

Assessment Team

The Assessment Director will lead the assessment team, which will consist of data collection technicians trained by the assessment director and experts in anthropometry, questionnaire administration and health literacy. The assessment team will be responsible for collecting data on the outcome measures at baseline, 4-week, 8-week, 6-month, 12-month, and 24-month visits.

Education Team

The Director of Education and his/her team will be responsible for developing an educational program on PA for the intervention group in the trial.

Timeline

The SAWA trial is designed as an 8-week intervention for rural older adults. The overall duration of this study is four years. The following figure shows the timeline of activities (by quarter) for the SAWA trial.

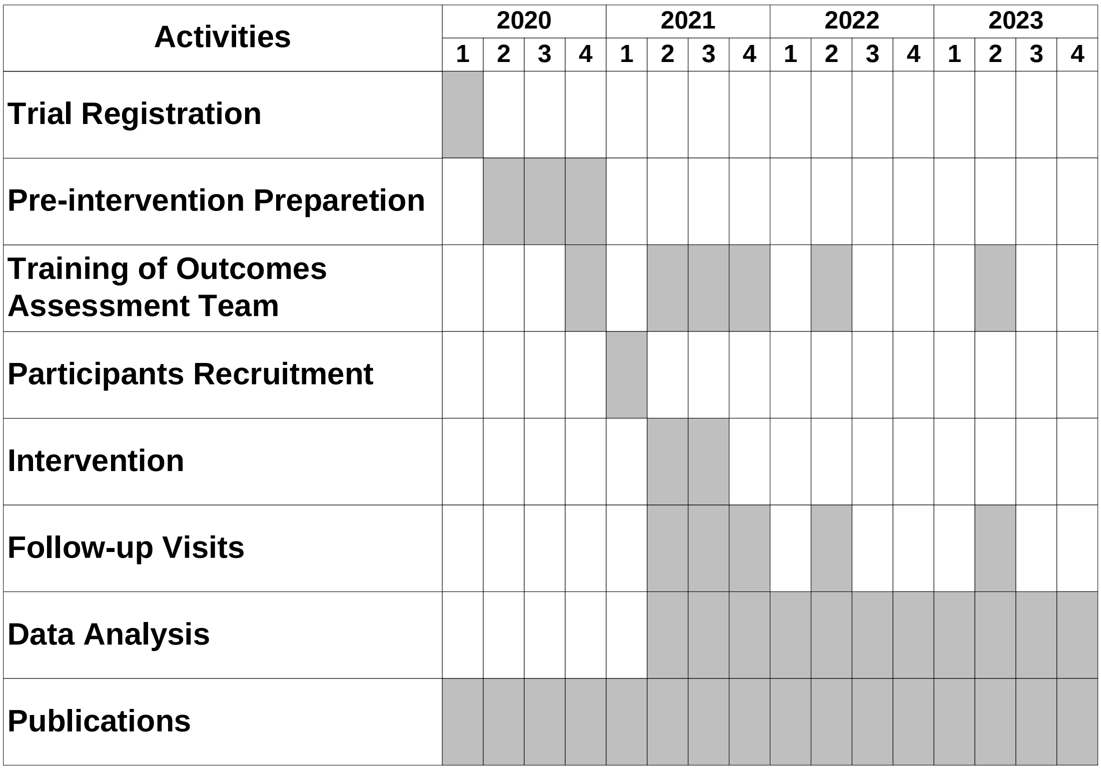

Figure 3. Timeline of Activities (by Quarter) for the SAWA Trial.

Literature Cited

1. Organization WH. Assessing national capacity for the prevention and control of noncommunicable diseases: report of the 2017 global survey. 2018.

2. Ma LW, Yazhe. Wang, Wen. Chen, Weiwei. Interpretation of the report on cardiovascular diseases in China (2017)(in Chinease). *Chin J Cardiovasc Med* 2018; **23**(01): 3-6.

3. Gu J. Interpretation of the report on nutrition and chronic diseases in China (2015)(in Chinease). *Acta Nutrimenta Sinica* 2016; **38**(06): 525-9.

4. Chen W. Report on cardiovascular diseases in China 2015 (in Chinese). *China Medical News* 2016; **0**(12).

5. Liu ML, Yichong. Liu,Shiwei,et al. Burden on blood-pressure-related diseases among the Chinese population,in 2010 (in Chinese). *Chinese Journal of Epidemiology* 2014; (6): 680-3.

6. Moore SC, Lee IM, Weiderpass E, et al. Association of Leisure-Time Physical Activity With Risk of 26 Types of Cancer in 1.44 Million Adults. *JAMA Intern Med* 2016; **176**(6): 816-25.

7. Kyu HH, Bachman VF, Alexander LT, et al. Physical activity and risk of breast cancer, colon cancer, diabetes, ischemic heart disease, and ischemic stroke events: systematic review and dose-response meta-analysis for the Global Burden of Disease Study 2013. *BMJ* 2016; **354**: i3857.

8. Brian W. Ward TCC, Colleen N. Nugent, Jeannine S. Schiller. Early release of selected estimates based on data from the 2015 National Health Interview Survey; 2016. 2016.

9. Zhu W, Chi A, Sun Y. Physical activity among older Chinese adults living in urban and rural areas: A review. *J Sport Health Sci* 2016; **5**(3): 281-6.

10. Wang R, Yan Z, Liang Y, et al. Prevalence and Patterns of Chronic Disease Pairs and Multimorbidity among Older Chinese Adults Living in a Rural Area. *PLoS One* 2015; **10**(9): e0138521.

11. Prince MJ, Wu F, Guo Y, et al. The burden of disease in older people and implications for health policy and practice. *Lancet* 2015; **385**(9967): 549-62.

12. Organization WH. Global recommendations on physical activity for health. 2010. 2015.

13. Wang DN, Morrow-Howell. Wu, Jilei. et al. The Physical Activity Status of Rural Elders and Environmental Factors Analysis in China (in Chinese). *Population and Development* 2017; **23**(03): 76-83.

14. Li S. Status Analysis of Physical Exercise of Agricultural Population in Rural-urban Fringe Zones and It's the Corresponding Strategy (in Chinese). *Journal of Chengdu Sport University* 2014; **40**(10): 52-4.

15. Zhou J, Britigan DH, Rajaram SS, Wang H, Su D. Association between leisure time physical activity preference and behavior: evidence from the China Health & Nutrition Survey, 2004-2011. *BMC Public Health* 2017; **17**(1): 451.

16. Zhou J, Wang H, Rajaram SS, Britigan DH, Su D. Changes in Leisure Time Physical Activity Preferences and Hypertension Risk. *Am J Health Behav* 2019; **43**(1): 145-57.

17. Wang Z, Xu F, Ye Q, et al. Childhood obesity prevention through a community-based cluster randomized controlled physical activity intervention among schools in china: the health legacy project of the 2nd world summer youth olympic Games (YOG-Obesity study). *Int J Obes (Lond)* 2018; **42**(4): 625-33.

18. Chen Y, Ma L, Ma Y, et al. A national school-based health lifestyles interventions among Chinese children and adolescents against obesity: rationale, design and methodology of a randomized controlled trial in China. *BMC Public Health* 2015; **15**: 210.

19. Shumway-Cook A, Brauer S, Woollacott M. Predicting the probability for falls in community-dwelling older adults using the Timed Up & Go Test. *Phys Ther* 2000; **80**(9): 896-903.

20. Greaney ML, Riebe D, Ewing Garber C, et al. Long-term effects of a stage-based intervention for changing exercise intentions and behavior in older adults. *Gerontologist* 2008; **48**(3): 358-67.

21. Harden SM, Johnson SB, Almeida FA, Estabrooks PA. Improving physical activity program adoption using integrated research-practice partnerships: an effectiveness-implementation trial. *Transl Behav Med* 2017; **7**(1): 28-38.

22. Martinson BC, Sherwood NE, Crain AL, et al. Maintaining physical activity among older adults: 24-month outcomes of the Keep Active Minnesota randomized controlled trial. *Prev Med* 2010; **51**(1): 37-44.

23. Glanz K, Rimer BK, Viswanath K. Health behavior: Theory, research, and practice: John Wiley & Sons; 2015.

24. Uchendu C, Windle R, Blake H. Perceived Facilitators and Barriers to Nigerian Nurses' Engagement in Health Promoting Behaviors: A Socio-Ecological Model Approach. *Int J Environ Res Public Health* 2020; **17**(4).

25. Sallis JF, Owen N, Fisher E. Ecological models of health behavior. *Health behavior: Theory, research, and practice* 2015; **5**(43-64).

26. Mehtala MA, Saakslahti AK, Inkinen ME, Poskiparta ME. A socio-ecological

approach to physical activity interventions in childcare: a systematic review. *Int J Behav Nutr Phys Act* 2014; **11**: 22.

27. Soderlund PD. The Social Ecological Model and Physical Activity Interventions for Hispanic Women With Type 2 Diabetes: A Review. *J Transcult Nurs* 2017; **28**(3): 306-14.

28. Rich P, Aarons GA, Takemoto M, et al. Implementation-effectiveness trial of an ecological intervention for physical activity in ethnically diverse low income senior centers. *BMC Public Health* 2017; **18**(1): 29.

29. Rosenberg DE. Outcomes of a multilevel walking intervention for older adults living in retirement communities: University of California, San Diego and San Diego State University; 2010.

30. Bull FC, Al-Ansari SS, Biddle S, et al. World Health Organization 2020 guidelines on physical activity and sedentary behaviour. *Br J Sports Med* 2020; **54**(24): 1451-62.

31. Huston P, McFarlane B. Health benefits of tai chi: What is the evidence? *Can Fam Physician* 2016; **62**(11): 881-90.

32. Lan C, Chen SY, Lai JS. The exercise intensity of Tai Chi Chuan. *Med Sport Sci* 2008; **52**: 12-9.

33. Ngai SP, Cheung RT, Lam PL, Chiu JK, Fung EY. Validation and reliability of the Physical Activity Scale for the Elderly in Chinese population. *J Rehabil Med* 2012; **44**(5): 462-5.

34. Vaughan K, Miller WC. Validity and reliability of the Chinese translation of the Physical Activity Scale for the Elderly (PASE). *Disabil Rehabil* 2013; **35**(3): 191-7.

35. Washburn RA, McAuley E, Katula J, Mihalko SL, Boileau RA. The physical activity scale for the elderly (PASE): evidence for validity. *J Clin Epidemiol* 1999; **52**(7): 643-51.

36. Washburn RA, Smith KW, Jette AM, Janney CA. The Physical Activity Scale for the Elderly (PASE): development and evaluation. *J Clin Epidemiol* 1993; **46**(2): 153-62.

37. Physical Activity Scale for the Elderly: Administration and Scoring Instruction Manual. 1991. https://meetinstrumentenzorg.nl/wp-content/uploads/instrumenten/PASE-handl.pdf.

38. Resnick B, Jenkins LS. Testing the reliability and validity of the Self-Efficacy for Exercise scale. *Nurs Res* 2000; **49**(3): 154-9.

39. Umstattd MR, Motl R, Wilcox S, Saunders R, Watford M. Measuring physical activity self-regulation strategies in older adults. *J Phys Act Health* 2009; **6 Suppl 1**: S105-12.

40. Rong H, Lai X, Mahmoudi E, Fang H. Early-Life Exposure to the Chinese Famine and Risk of Cognitive Decline. *J Clin Med* 2019; **8**(4).

41. Xu H, Zhang Z, Li L, Liu J. Early life exposure to China's 1959-61 famine and midlife cognition. *Int J Epidemiol* 2018; **47**(1): 109-20.

42. Fu C, Li Z, Mao Z. Association between Social Activities and Cognitive Function among the Elderly in China: A Cross-Sectional Study. *Int J Environ Res Public Health* 2018; **15**(2).

43. Li J, Cacchione PZ, Hodgson N, et al. Afternoon Napping and Cognition in Chinese Older Adults: Findings from the China Health and Retirement Longitudinal Study Baseline Assessment. *J Am Geriatr Soc* 2017; **65**(2): 373-80.

44. Buysse DJ, Reynolds CF, 3rd, Monk TH, Berman SR, Kupfer DJ. The Pittsburgh Sleep Quality Index: a new instrument for psychiatric practice and research. *Psychiatry Res* 1989; **28**(2): 193-213.

45. Tsai PS, Wang SY, Wang MY, et al. Psychometric evaluation of the Chinese version of the Pittsburgh Sleep Quality Index (CPSQI) in primary insomnia and control subjects. *Qual Life Res* 2005; **14**(8): 1943-52.

46. Blackford K, Jancey J, Lee AH, et al. A randomised controlled trial of a physical activity and nutrition program targeting middle-aged adults at risk of metabolic syndrome in a disadvantaged rural community. *BMC Public Health* 2015; **15**: 284.

47. Perloff D, Grim C, Flack J, et al. Human blood pressure determination by sphygmomanometry. *Circulation* 1993; **88**(5 Pt 1): 2460-70.

48. James GD, Gerber LM. Measuring arterial blood pressure in humans: Auscultatory and automatic measurement techniques for human biological field studies. *American journal of human biology*: the official journal of the Human Biology Council 2018; **30**(1).

49. Conn VS, Valentine JC, Cooper HM. Interventions to increase physical activity among aging adults: a meta-analysis. *Ann Behav Med* 2002; **24**(3): 190-200.

50. Ribeiro DC, Milosavljevic S, Abbott JH. Sample size estimation for cluster randomized controlled trials. *Musculoskelet Sci Pract* 2018; **34**: 108-11.

Appendices

Appendix A: SAWA Forms and Questionnaires

Appendix B: Intervention Materials

Appendix C: SAWA Recruitment Materials

Appendix A: SAWA Forms and Questionnaires

- **Baseline Demographics, Health Behavior, and Health History Questionnaire**
- **Physical Activity Scale for the Elderly**
- **Leisure-time Activity Preferences Measurement Form**
- **Sedentary Behavior Measurement Form**
- **Self-Efficacy for Exercise Scale**
- **Physical Activity Self-Regulation Scale Telephone Interview for Cognitive Status**
- **Pittsburgh Sleep Quality Index**
- **Adverse Event Log**
- **Visit Checklist**

Baseline Demographics, Health Behaviors, and Health History Questionnaire

1. Basic Information

1.1 Participant's name: _______________

1.2 Village: (1) Guilin; (2) Jianzheng; (3) Qianfeng; (4) Yixue; (5) Xinsheng; (6) Yijia; (7) Huanglian; (8) Tiane

1.3 ID number: _______________

1.4 Investigator 's Signature: _______________

1.5 Date: _______________

2. Socio-demographic Characteristics

2.1 Sex: (1) male; (2) female

2.2 Marital status: (1) married/cohabited; (2) widowed; (3) separated/divorced; (4) never married

2.3 Education level: (1) not formally attended school; (2) primary school drop-outs; (3) primary school; (4) middle school; (5) high school and above

2.4 Employment: (1) yes; (2) no

2.5 Family size: _______________

2.6 Annual household income (RMB): (1) <12000; (2) 12000-19999; (3) 20000-59999; (4) ≥60000

3. Physical Health and Function

3.1 The scale below has numbers from 0 to 100, with 100 representing the best health you can imagine and 0 representing the worst health you can imagine. Your health status today is on the scale of: ______

|-----|-----|-----|------|-----|-----|------|------|-----|-------|

0 100

3.2 Have you ever been diagnosed with any of the following diseases by a doctor at a township/district hospital or above? Please answer in each item and if yes, please provide further explanation.

	Do you have this disease?	Medication usage	Is blood pressure/blood sugar normal?
Hypertension	(1) yes<br>(2) no	(1) take regularly<br>(2) take intermittently<br>(3) never take	(1) yes<br>(2) no<br>(3) I don’t know
Diabetes	(1) yes<br>(2) no	(1) take regularly<br>(2) take intermittently<br>(3) never take	(1) yes<br>(2) no<br>(3) I don’t know

3.3 Have you been diagnosed with any of the following diseases?

3.3.1 coronary heart disease (1) yes; (2) no

3.3.2 chronic bronchitis/emphysema (1) yes; (2) no

3.3.3 asthma (1) yes; (2) no

3.3.4 psychiatric disorders (1) yes; (2) no

3.3.5 malignant tumors* (1) yes; (2) no

*If you have malignant tumor, please indicate the specific site: ______ (If more than one site, please fill in the first tumor site)

(1) lung; (2) esophagus; (3) stomach; (4) liver; (5) intestines; (6) breast; (7) prostate; (8) cervix; (9) other

4. Daily Behavior

4.1 Smoking status. Please select the option that fits your situation.

(1) never

(2) former (quit smoking for more than six months or more)

(3) current (total smoking to date is over 100 cigarettes)

4.2 Alcohol consumption. How did you drink in the past year, including beer, wine or liquor?

(1) never / seldom; (2) $<$ once a month; (3) $\geq$ once a month

4.3 Daytime napping

4.3.1 I have the habit of taking daytime napping.   (1) yes; (2) no (end)

4.3.2 Usually, I take daytime napping ______ day(s) a week.

4.3.3 Usually, I take an average daytime napping of ______ minutes a day.

Physical Activity Scale for the Elderly

1. Leisure-time Physical Activity

1.1 Have you walked outside home in the past week? For example, walking the dog, exercising, and walking to and from work.

(1) never (skip to question 1.1.c); (2) seldom (1-2 days); (3) sometimes (3-4 days); (4) often (5-7 days)

1.1.a How much time do you usually spend doing these things each day?

(1) < 1 hour; (2) 1-2 hours; (3) 2-4 hours; (4) > 4 hours

1.1.b How many kilometers do you usually walk per day?

(1) < 0.5 km; (2) 0.5-1 km; (3) 1-2 km; (4) > 2 km

1.2 In the past week, how did you participate in light sports such as fishing?

(1) never (skip to question 1.2.c); (2) seldom (1-2 days); (3) sometimes (3-4 days); (4) often (5-7 days)

1.2.a What are the specific activities: ______

1.2.b How much time do you usually spend doing these things each day?

(1) < 1 hour; (2) 1-2 hours; (3) 2-4 hours; (4) > 4 hours

1.3 In the past week, how did you participate in moderate sports such as table tennis, dancing, or tai chi?

(1) never (skip to question 1.3.c); (2) seldom (1-2 days); (3) sometimes (3-4 days); (4) often (5-7 days)

1.3.a What are the specific activities: ______

1.3.b How much time do you usually spend doing these things each day?

(1) < 1 hour; (2) 1-2 hours; (3) 2-4 hours; (4) > 4 hours

1.4 In the past week, how did you participate in strenuous sports such as jogging, cycling, or swimming?

(1) never (skip to question 1.4.c); (2) seldom (1-2 days); (3) sometimes (3-4 days); (4) often (5-7 days)

1.4.a What are the specific activities: ______

1.4.b How much time do you usually spend doing these things each day?

(1) < 1 hour; (2) 1-2 hours; (3) 2-4 hours; (4) > 4 hours

1.5 In the past week, how did you participate in muscle strength / endurance exercises such as weight lifting, bench press, or push-ups?

(1) never (skip to question 1.5.c); (2) seldom (1-2 days); (3) sometimes (3-4 days); (4) often (5-7 days)

1.5.a What are the specific activities: ______

1.5.b How much time do you usually spend doing these things each day?

(1) < 1 hour; (2) 1-2 hours; (3) 2-4 hours; (4) > 4 hours

2. Household Physical Activity

2.1 In the past week, did you participate in light housework such as washing dishes or sweeping the floor?

(1) no (skip to question 2.2); (2) yes

2.1.a How much time do you spend on light housework for a week: ______ hours

2.2 In the past week, did you participate in heavy housework or chores such as wiping floors, cleaning windows, or moving things?

(1) no (skip to question 2.3); (2) yes

2.2.a How much time do you spend on heavy housework or chores for a week: ______ hours

2.3 In the past week, did you participate in the following activities?

Items	Yes	No
a. Home repairs such as repairing electrical appliances, or doing carpentry	1	2
b. Lawn work or yard care	1	2
c. Outdoor gardening	1	2
d. Caring for another person	1	2

3. Work-related Physical Activity

3.1 In the past week, did you work for pay or as a volunteer?

(1) no (end); (2) yes

3.1.a How much time do you work for pay or as a volunteer for a week: ______ hours

3.1.b Which of the following descriptions best presents your work for pay or as a volunteer

(1) frequent sitting, light upper extremity activity (e.g., office work, ticket takers, etc.)

(2) sitting or standing often, with only a small amount of walking (e.g., cashiers, etc.)

(3) often running, need to carry general heavy objects (such as waiters, letter carriers, etc.)

(4) frequent running, need to carry quite heavy objects (such as construction workers)

Leisure-time Activity Preferences

1.1 Do you like walking outside the home?

(1) strongly dislike; (2) dislike somewhat; (3) neutral; (4) like somewhat; (5) strongly like

1.2 Do you like light-intensity sports?

(1) strongly dislike; (2) dislike somewhat; (3) neutral; (4) like somewhat; (5) strongly like

1.3 Do you like moderate-intensity sport?

(1) strongly dislike; (2) dislike somewhat; (3) neutral; (4) like somewhat; (5) strongly like

1.4 Do you like vigorous-intensity sport?

(1) strongly dislike; (2) dislike somewhat; (3) neutral; (4) like somewhat; (5) strongly like

1.5 Do you like muscle strength exercises?

(1) strongly dislike; (2) dislike somewhat; (3) neutral; (4) like somewhat; (5) strongly like

Sedentary Behavior Measurement Form

1.1 In the past week, how much time in total did you spend sitting and doing the following intellectual activities during your free time?

1.1.a Play chess (including Chinese chess, go, checkers, etc.) ______ hours ______ minutes

1.1.b Play tiles (mahjong, poker, and solitaire) ______ hours ______ minutes

1.1.c Calligraphy, painting, reading, and writing ______ hours ______ minutes

1.2 In the past week, how much time in total did you spend sitting in your free time (including watching TV, riding in the car, talking, needlework, etc.) ______ hours ______ minutes

Self-Efficacy for Exercise Scale

How confident are you right now that you could exercise three times per week for 20 minutes if:

Item	Not Confident										Very Confident
1.1 the weather was bothering you	0	1	2	3	4	5	6	7	8	9	10
1.2 you were bored by the program or activity	0	1	2	3	4	5	6	7	8	9	10
1.3 you felt pain when exercising	0	1	2	3	4	5	6	7	8	9	10
1.4 you had to exercise alone	0	1	2	3	4	5	6	7	8	9	10
1.5 you did not enjoy it	0	1	2	3	4	5	6	7	8	9	10
1.6 you were too busy with other activities	0	1	2	3	4	5	6	7	8	9	10
1.7 you felt tired	0	1	2	3	4	5	6	7	8	9	10
1.8 you felt stressed	0	1	2	3	4	5	6	7	8	9	10
1.9 you felt depressed	0	1	2	3	4	5	6	7	8	9	10

Physical Activity Self-Regulation Scale

Please comment on the following questions according to your actual situation.

Items	Never	Rarely	Sometimes	Often	Very often
1.1 I mentally kept track of my PA	1	2	3	4	5
1.2 I mentally noted specific things that helped me be active	1	2	3	4	5
1.3 I set short term goals for PA	1	2	3	4	5
1.4 I asked someone for PA advice or demo	1	2	3	4	5
1.5 I asked a PA expert or health professional for PA advice or demo	1	2	3	4	5
1.6 I reminded myself of PA health benefits	1	2	3	4	5
1.7 I mentally scheduled specific times for PA	1	2	3	4	5
1.8 I rearranged my schedule to ensure I had time for PA	1	2	3	4	5
1.9 I purposely planned ways to do PA when on trips away from home	1	2	3	4	5
1.10 I purposely planned ways to do PA in bad weather	1	2	3	4	5

Abbreviations: PA, physical activity; demo, demonstration.

Telephone Interview for Cognitive Status

Now I will ask you a few questions, some of which may be easy for you and some of which may be difficult.

1.1 What is today's year/month/date?

year (1) correct; (2) incorrect

month (1) correct; (2) incorrect

date (1) correct; (2) incorrect

1.2 What is the day of the week? (1) correct; (2) incorrect

1.3 What season are we in? (1) correct; (2) incorrect

1.4 How do you feel about your memory now?

(1) excellent; (2) very good; (3) good; (4) average; (5) bad

1.5 I'm going to read you a list of ten words. Please listen carefully and try to remember them. When I am done, tell me as many words as you can, in any order. Ready?

The words are: hat, car, tree, elephant, cinema, watch, pillow, hospital, pencil, table tennis.

Now tell me all the words you can remember. Answered correctly ______ words.

1.6 We will ask you some subtractions.

(1) One hundred minus 7 equals what? ______

(2) And 7 from that (the answer to (1))? ______

(3) And 7 from that (the answer to (2))? ______

(4) And 7 from that (the answer to (3))? ______

(5) And 7 from that (the answer to (4))? ______

The number of answers correctly _____

1.7 The ten words I read to you earlier, please tell me the words you remember now.

Answered correctly ______ words.

Pittsburgh Sleep Quality Index

	Items	Options			
		(1)	(2)	(3)	(4)
1.1	During the past month, what time have you usually gone to bed at night? Bedtime ______				
1.2	During the past month, how long (in minutes) has it usually taken you to fall asleep each night? Number of minutes ______	≤ 15 min	16~30 min	31~60 min	> 60 min
1.3	During the past month, what time have you usually gotten up in the morning? Getting up time ______				
1.4	During the past month, how many hours of actual sleep did you get at night? (This may be different than the number of hours you spent in bed.) Hours of sleep per night ______				
1.5	During the past month, how often you had trouble sleeping because you…				
	a. Cannot get to sleep within 30 minutes	not during the past month	less than once a week	once or twice a week	three or more times a week
	b. Wake up in the middle of the night or early morning	not during the past month	less than once a week	once or twice a week	three or more times a week
	c. Have to get up to use the bathroom	not during the past month	less than once a week	once or twice a week	three or more times a week

	d. Cannot breathe comfortably	not during the past month	less than once a week	once or twice a week	three or more times a week
	e. Cough or smore loudly	not during the past month	less than once a week	once or twice a week	three or more times a week
	f. Feel too cold	not during the past month	less than once a week	once or twice a week	three or more times a week
	g. Feel too hot	not during the past month	less than once a week	once or twice a week	three or more times a week
	h. Had bad dreams	not during the past month	less than once a week	once or twice a week	three or more times a week
	i. Have pain	not during the past month	less than once a week	once or twice a week	three or more times a week
	j. Other reason(s), please describe ______. How often during the past month have you had trouble sleeping because of this?	not during the past month	less than once a week	once or twice a week	three or more times a week
1.6	During the past month, how would you rate your sleep quality overall?	very good	fairly good	fairly bad	very bad
1.7	During the past month, how often have you taken medicine to	not during the past month	less than once a week	once or twice a week	three or more times a week

	help you sleep (prescribed or "over the counter")?				
1.8	During the past month, how often have you had trouble staying awake while driving, eating meals, or engaging in social activity?	not during the past month	less than once a week	once or twice a week	three or more times a week
1.9	During the past month, how much of a problem has it been for you to keep up enough enthusiasm to get things done?	no problem at all	only a very slight problem	somewhat of a problem	a very big problem

Adverse Event Log

Participants' ID number	Date Reported (mm/dd/yyyy)	Adverse Event	Start Date/Time (mm/dd/yyyy 2400 clock)	Outcome 1=Resolved 2= Resolved w/sequelae 3=Recovering 4=Unknown 5=Fatal	End Date/Time (mm/dd/yyyy 2400 clock)	Severity 1= mild 2= mod 3= severe	Action Taken as a result of Adverse Event 0= None 1= Refer to Primary Care Physician 2= Other; please explain	Withdrawal Did subject withdraw from the study as a result of this AE? Yes/No	Is Adverse Event serious? (Determined by the Physician) Yes/No	Related to Intervention (Completed by the Physician) 1=Definitely 2=Probably 3=Possibly 4=Unrelated 5=Unknown	Serious of Adverse Event (Determined by the Physician) Yes/No	Unexpected Adverse Event (Completed by the Physician) Was the AE unexpected in nature, severity, or frequency? Yes/No

Visit Checklist

Village: (1) Guilin; (2) Jianzheng; (3) Qianfeng; (4) Yixue; (5) Xinsheng; (6) Yijia; (7) Huanglian; (8) Tiane

Participant's name: ____________ Sex: Male ☐ Female ☐

Tel: ☐☐☐☐☐☐☐☐☐☐☐

ID number: ☐☐☐☐☐☐☐☐☐☐☐☐☐☐☐☐☐☐

No.	SAWA Assessments	Notes	Investigator's Signature
1	Weight	kg	
2	Percentage Body Fat	%	
3	Visceral Fat		
4	Height	cm	
5	Waist Circumference	cm	
6	Hip Circumference	cm	
7	Blood Pressure (Systolic/Diastolic Pressure)	mmHg<br>mmHg<br>mmHg	
8	Questionnaires	Yes☐ No☐	

Quality Control Staff' Signature____________ Date____________

Appendix B: Intervention Materials

- **Telephone Counseling Techniques**
- **Telephone Counseling Record**

Telephone Counseling Techniques

(1) Telephone Counseling Template for the First Time

a. Telephone counseling process (group leader)

1. First of all, please ask: Hello, is this xxx?

2. Descript the intention of this call: We are the investigators performed physical activity (PA) training and questionnaire survey for you several days ago. Now we need to ask you some questions, can you please make it convenient for us now? (if it's not convenient now, ask the nearest available time)

3. Primary contents

1) Recent exercise situation: What types of exercise were you performing recently, how many times a day, and how many minutes each time for performing PA?

2) Set exercise goals: Your current exercise intensity is (great/okay/not enough), next week you can perform (moderate-intensity PA such as Tai Chi: 2 times a day and 20 minutes for each time or 40 minutes a day; vigorous-intensity PA such as running: 2 times a day and 15 minutes for each time or 20 minutes a day). We will call you every week to see if you have reached your goals.

3) Peer group: We assisted you form a peer group to exercise together after the PA training, do you remember that? You are the leader of group x (e.g., 1) and the group has xx members, including xx, xx, and xx. What kind of exercise do you think is better for your group to perform together? How many times a week can you get together to exercise, and how many minutes for each time? Okay, then your group exercise goal is to xx (e.g., dance five times a week and 40 minutes for each time). Do you think it is okay? Please don't forget to organize your members to exercise together.

b. Telephone counseling process (all intervention subjects)

1. First of all, please ask: Hello, is this xxx?

2. Descript the intention of this call: We are the investigators performed physical activity training and questionnaire survey for you several days ago. Now we need to ask you some questions, can you please make it convenient for us now? (if it’s not convenient now, ask the nearest available time)

3. Primary contents

1) Recent exercise situation: What types of exercise were you performing recently, how many times a day, and how many minutes each time for performing PA?

2) Set exercise goals: Your current exercise intensity is (great/okay/not enough), next week you can perform (moderate-intensity PA such as Tai Chi: 2 times a day and 20 minutes for each time or 40 minutes a day; vigorous-intensity PA such as running: 2 times a day and 15 minutes for each time or 20 minutes a day). We will call you every week to see if you have reached your goals.

3) Peer group: We assisted you to form a peer group to exercise together after the PA training, do you remember that? You are one of the members of group x (e.g., 1), and xx is the leader of your group. The exercise goal of your group is to xx (e.g., dance once a week and 20 minutes for each time). Please don’t forget to exercise with your group.

(2) Weekly Telephone Counseling Template After the First Time

a. Telephone counseling process (group leaders)

1. First of all, please ask: Hello, is this xxx?

2. Descript the intention of this call: We are the investigators performed physical activity training and questionnaire survey for you. Now we need to ask about your exercise situation for the previous week, which is estimated to take 3 minutes, would that be convenient for you? (if it’s not convenient now, ask the nearest available time)

3. Primary contents

1) Personal exercise goal: The exercise goal we set in counseling was xx (e.g., play Tai

Chi twice a day and 20 minutes for each time). Did you reach that goal last week? Do you think the current intensity and amounts of PA are appropriate?

2) Current difficulties: What were the reasons for your failure to reach the goal? Did you encounter any difficulties?

3) Peer group: You are the leader of your peer group, right? Did you organize your members to exercise together regularly? Have you reached the goal of xx (e.g., dancing once a week)?

a. If the goal was not achieved, ask why and record it.

b. If the goal was achieved, know about the positivity of group members, and if they were active, adjust the goal by increasing the amount and intensity of PA appropriately.

b. Telephone counseling process (all intervention subjects)

1. First of all, please ask: Hello, is this xxx?

2. Descript the intention of this call: We are the investigators performed physical activity training and questionnaire survey for you about x (e.g., 2) weeks ago. Now we need to ask about your exercise situation for the previous week, which is estimated to take 3 minutes, would that be convenient for you? (if it's not convenient now, ask the nearest available time)

3. Primary contents

1) Personal exercise goal: The exercise goal we set in the last counseling was xx (e.g., play Tai Chi twice a day and 20 minutes for each time). Did you reach that goal last week? Do you think the current intensity and amounts of PA are appropriate?

2) Current difficulties: What were the reasons for your failure to reach the goal? Did you encounter any difficulties?

3) Self-efficacy: Which aspects do you think you are doing well in your exercise, could you be more specific? (deliver encouragement). Are there any areas where you are not

doing well? What adjustments can be made in these areas?

4) Peer group: Have you participated in regular peer group activities (e.g., dance) during last week? Did the group leader (xxx) lead you to exercise regularly?

(3) Communication Skills

1. Praising them for doing well in some aspects in exercise. For example, “You exercised x days last week and x hours for each day. You are very active! Keep it up and it will promote your cardiovascular and sleep health (better be tailored, e.g., if you know the participant has sleep problems, you could tell him/her physical activity is good for sleep health)!”, or “You have improved much than before! You spent more time and exercised more often than before!”

2. When participants mention that they exercised less last week for various reasons. We should express understanding and try to attribute the reason to external factors which are relatively easy to change. For example:

1) “It did rain almost every day last week and it was not convenient to exercise outside. But you can perform exercise at home on rainy days in the future so that keep your body moving and healthy.”

2) “It is really important to spend more time to company your granddaughter. How old is your granddaughter? You can also take her walk around these days, and exercise together.”

3) Encourage them to readjust their goals according to their situation and ask about whether they reach that goal at the next counseling.

Telephone Counseling Record

Basic Information			Telephone Counseling Record at 1 Week							
			Personal Goals		Group Goals					
Group Number	Participants' Name	Telephone Number	Types of Exercise	Exercise Times/Week	Types of Exercise	Group Exercise (Times/Week)	Time for Per Session (min)	Special Cases at 1 Week	Counseling Date	Investigator's Name

Basic Information			Telephone Counseling Record at X Weeks (After 1 Week)														
			Personal Goals							Group Goals							
						Adjust Personal Goals		Group Exercise		Group Exercise		Adjust Group Goals					
Group Number	Participants' Name	Telephone Number	Whether the Personal Goals were Reached Last Week	Reasons for Did Not Reach the Personal Goals	Measures to Assistant	Types of Exercise	Exercise Times/Week	Whether Absent from Group Exercise	Reasons for Absence	Whether the Group Goals were Reached Last Week	Reasons for Did Not Reach the Group Goals	Types of Exercise	Group Exercise (Times/Week)	Time for Per Session (min)	Special Cases at X Weeks	Counseling Date	Investigator's Name

Appendix C: SAWA Recruitment Materials

Individuals with 60 years of age or older were invited to participate.

Exclusion criteria: people screened for eligibility who meet any one of the following criteria cannot be our participants.

1) have a history of stroke, arthritis, Parkinson's disease, severe pneumonia or severe heart disease;

2) have severe cognitive or hearing disorders;

3) had a major operation in the last 3 years;

4) poor control of hypertension or diabetes;

5) being treated for cancer;

6) have fallen during the last 12 months.

Inclusion criteria: people screened for eligibility who meet the following 4 items can be our participants.

1) be able to answer the phone;

2) be able to cover 400 meters within 15 minutes;

3) be able to walk without assistance or crutches;

4) be able to complete the Timed Up & Go test (the process is shown below).

When I send out the command "Go", please

e. Get up from your chair;
f. Walk 3 meters in a straight line at your usual speed, and you can use a cane;
g. Turn around after walking 3 meters, then continue to walk 3 meters in a straight line at your usual speed and return to the chair;

Note: The timing method starts from [giving instructions] and ends at [sitting on the chair again], and compares with 12s.

Summary of Changes to the Original Protocol

Changes to Outcomes

(1) primary outcome

The final protocol has a clearer definition of the primary outcome than the original protocol. The primary outcome in the original protocol is the PA level. To gain a detailed understanding of the effects of the trial in promoting PA, we divided PA into leisure-time activity, household activity, work-related activity, and total PA. Since the 8-week intervention trial mainly focused on leisure-time activity promotion, the primary outcome is changes in leisure-time activity at 8 weeks in the final protocol. And we added the "household activity", "work-related activity", and "total physical activity" into the secondary outcomes.

(2) secondary outcomes

Previous intervention studies used changes in absolute physical activity level as outcome measures, without considering the proportions of participants meeting the WHO physical activity recommendations which could be helpful to understand the clinical relevance of changes. Therefore, we included the "proportions of meeting WHO physical activity recommendations" in the secondary outcomes.

The previous observational study revealed that the leisure-time preferences were significantly associated with actual activity and can predict the level of leisure-time activity. To examine the effects of the PA intervention on leisure-time activity preferences, we added the leisure-time activity preferences into the secondary outcomes.

We also included the self-rated health in the secondary outcomes in the final protocol. This change allowed us to examine the effects of the PA intervention on self-rated health which is considered as a sensitive measure of overall health in older adults.

Besides, the systolic and diastolic blood pressure were determined using the average of the second two readings in the final version, rather than "the average of the

three readings” in the original protocol, based on literature and expert advice.

Changes to Data Analysis Plan

To assess the sensitivity and robustness of the results, we included “multiple imputation” in the final protocol to handle the missing data. Furthermore, in line with previous community intervention studies, analyses were carried out on an intention-to-treat basis. Thus, the compliance analysis was removed from the final protocol.

Changes to Timeline

The assessment is conducted at baseline, 4-week, 8-week, 6-month, 12-month, and 24-month visits. The timeline in the original protocol missed the 24-month visit, which is expected to be conducted in May 2023, so it has been corrected in the final version.
