# Supplementary material for: Physical Activity Intervention for Leisure-Time Activity Levels Among Older Adults: A Cluster Randomized Trial
Source: JAMA Netw Open. 2023 Sep 15;6(9):e2333195. doi: 10.1001/jamanetworkopen.2023.33195 (PMC10504609; doi:10.1001/jamanetworkopen.2023.33195)
Supplement: Supplement 2. — eFigure. Intervention Based on the Social-ecologic Model eTable 1. Attendance at Each Contact Point eTable 2. Effects of the Intervention and Time on Leisure-time Activity eTable 3. Effects of the Intervention and Time on Leisure-time Activity (Intention-to-Treat Methods Using Multiple Imputed Data) eTable 4. Differences in Leisure-time Activity According to Age Group eTable 5. Differences in Leisure-time Activity According to Sex eTable 6. Differences in Leisure-time Activity According to BMI eTable 7. Effects of the Intervention and Time on Secondary Outcomes of Participants (Intention-to-Treat Method Using Multiple Imputed Data) eTable 8. Effects of the Intervention and Time on Health Outcomes of Participants eTable 9. Details of Unexpected Serious Adverse Events [file jamanetwopen-e2333195-s002.pdf]

Supplementary Online Content

Li N, Ye Q, Deng Q, et al. Effect of a physical activity intervention on leisure-time activity level among older adults: a cluster randomized trial. *JAMA Netw Open*. 2023;1(3):e180818. doi:10.1001/jamanetworkopen.2018.0818

eFigure. Intervention Based on the Social-ecologic Model

eTable 1. Attendance at Each Contact Point

eTable 2. Effects of the Intervention and Time on Leisure-time Activity

eTable 3. Effects of the Intervention and Time on Leisure-time Activity (Intention-to-Treat Methods Using Multiple Imputed Data)

eTable 4. Differences in Leisure-time Activity According to Age Group

eTable 5. Differences in Leisure-time Activity According to Sex

eTable 6. Differences in Leisure-time Activity According to BMI

eTable 7. Effects of the Intervention and Time on Secondary Outcomes of Participants (Intention-to-Treat Method Using Multiple Imputed Data)

eTable 8. Effects of the Intervention and Time on Health Outcomes of Participants

eTable 9. Details of Unexpected Serious Adverse Events

This supplementary material has been provided by the authors to give readers additional information about their work.

© 2023 Li N et al. *JAMA Network Open.*

eFigure. Interventions Based on the Social-ecological Model

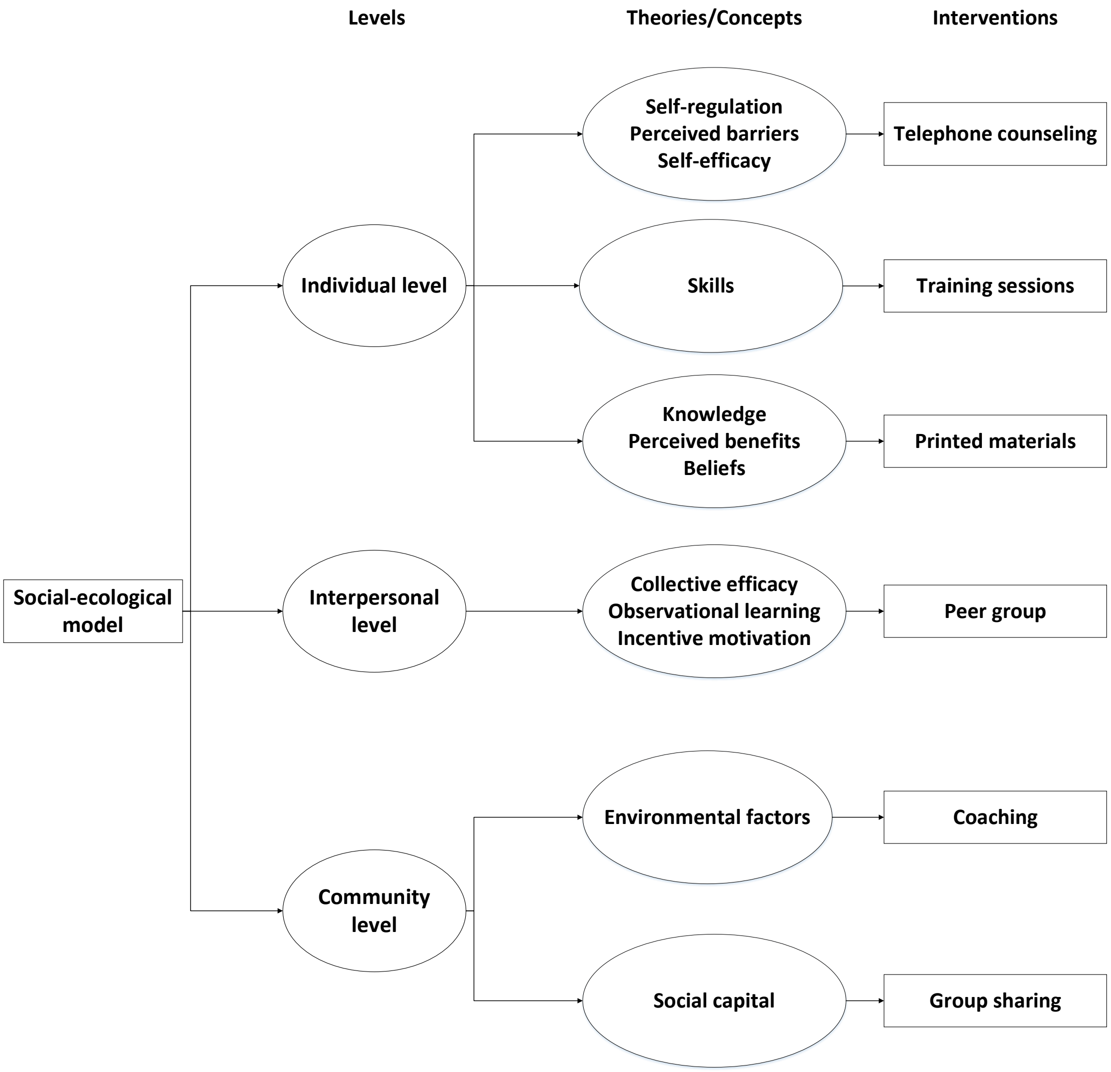

© 2023 Li N et al. *JAMA Network Open.*

eTable 1. Attendance at Each Contact Time Point

	Intervention Group (n=240)					Control Group (n=271)
Time Point	Follow-up visits attended	Training session/Coaching/Group sharing attended	Telephone counseling answered	Peer group attended	Exercise goal achieved	Follow-up visits attended
At baseline, No. (%)	-	240(100%)	-	-	-	-
At 1 week, No. (%)	-	-	219(91%)	-	-	-
At 2 weeks, No. (%)	-	-	216(90%)	163(68%)	193(80%)	-
At 3 weeks, No. (%)	-	-	191(80%)	167(70%)	198(83%)	-
At 4 weeks, No. (%)	185(77%)	185(77%)	206(86%)	182(76%)	209(87%)	237(87%)
At 5 weeks, No. (%)	-	-	199(83%)	162(68%)	204(85%)	-
At 6 weeks, No. (%)	-	-	197(82%)	179(75%)	213(89%)	-
At 7 weeks, No. (%)	-	-	183(76%)	176(73%)	212(88%)	-
At 8 weeks, No. (%)	205(85%)	205(85%)	192(80%)	167(70%)	216(90%)	243(90%)
At 6 months, No. (%)	207(86%)	-	-	-	-	238(88%)
At 12 months, No. (%)	200(83%)	-	-	-	-	222(82%)
At 24 months, No. (%)	193(80%)	-	-	-	-	229(85%)

© 2023 Li N et al. *JAMA Network Open.*

eTable 2. Effects of the Intervention and Time on Leisure-time Activity[a]

Outcomes	Increase from baseline, Mean (95%CI)		Intervention effect	*P*-Value
	Intervention group	Control group	Mean (95%CI)	
Leisure-time activity				
At 4 weeks	9.63 (5.74, 13.52)	-2.03 (-5.55, 1.49)	11.66 (6.41, 16.90)	**<0.001**[b]
At 8 weeks	11.33 (7.54, 15.12)	-2.42 (-5.92, 1.09)	13.74 (8.58, 18.91)	**<0.001**[b]
At 6 months	11.99 (8.23, 15.76)	-0.35 (-3.88, 3.18)	12.35 (7.19, 17.50)	**<0.001**[b]
At 12 months	7.26 (3.46, 11.07)	-4.29 (-7.88, -0.69)	11.55 (6.32, 16.78)	**<0.001**[b]
At 24 months	10.88 (7.04, 14.72)	-3.64 (-7.20, -0.07)	14.51 (9.28, 19.75)	**<0.001**[b]

[a] Calculated using time*group interaction term, indicating a differential increase from baseline to 4 weeks, baseline to 8 weeks, baseline to 6 months, baseline to 12 months, or baseline to 24 months; Covariates include age, sex, and baseline leisure-time activity adjusted; All the values presented in the table are model-based estimates.

[b] The difference of multiple comparison was significant, which we corrected using the Bonferroni method, $\alpha=0.05/5$.

© 2023 Li N et al. *JAMA Network Open.*

eTable 3. Effects of the Intervention and Time on Leisure-time Activity (Intention-to-Treat Method Using Multiple Imputed Data)[a]

Outcomes	Increase from baseline, Mean (95%CI)		Intervention effect	*P*-Value
	Intervention group	Control group	Mean (95%CI)	
Leisure-time activity				
At 4 weeks	9.47 (5.58, 13.37)	-2.07 (-5.59, 1.46)	11.54 (6.29, 16.79)	**<0.001**[b]
At 8 weeks	10.93 (7.15, 14.70)	-2.60 (-6.10, 0.89)	13.53 (8.39, 18.68)	**<0.001**[b]
At 6 months	11.84 (8.08, 15.61)	-0.18 (-3.70, 3.34)	12.02 (6.87, 17.18)	**<0.001**[b]
At 12 months	7.08 (3.28, 10.89)	-4.35 (-7.94, -0.76)	11.44 (6.21, 16.66)	**<0.001**[b]
At 24 months	10.76 (6.91, 14.60)	-3.70 (-7.26, -0.14)	14.46 (9.22, 19.70)	**<0.001**[b]

[a] Calculated using time*group interaction term, indicating a differential increase from baseline to 4 weeks, baseline to 8 weeks, baseline to 6 months, baseline to 12 months, or baseline to 24 months; Covariates include age, sex, and baseline leisure-time activity adjusted; All the values presented in the table are model-based estimates.

[b] The difference of multiple comparison was significant, which we corrected using the Bonferroni method, α=0.05/5.

© 2023 Li N et al. *JAMA Network Open.*

eTable 4. Differences in Leisure-time Activity According to Age Group[a]

Outcomes	Age 60-69, y Increase from baseline, Mean (95%CI) Intervention group	Control group	Intervention effect Mean (95%CI)	*P*-value	Age ≥70, y Increase from baseline, Mean (95%CI) Intervention group	Control group	Intervention effect Mean (95%CI)	*P*-value
At 4 weeks	13.03 (8.03, 18.03)	3.43 (-1.29, 8.16)	9.60 (2.72, 16.48)	**0.006**	7.56 (3.58, 11.54)	-6.32 (-9.82, -2.82)	13.88 (8.57, 19.18)	**<0.001**
At 8 weeks	12.18 (7.67, 16.69)	2.60 (-1.61, 6.82)	9.58 (3.44, 15.72)	**0.001**	10.84 (6.93, 14.75)	-6.40 (-9.97, -2.83)	17.25 (11.95, 22.54)	**<0.001**
At 6 months	15.17 (9.74, 20.60)	0.42 (-4.77, 5.60)	14.75 (7.23, 22.27)	**<0.001**	9.87 (4.98, 14.76)	-1.39 (-5.89, 3.12)	11.26 (4.63, 17.89)	**0.001**
At 12 months	8.96 (3.79, 14.13)	-1.56 (-6.54, 3.43)	10.52 (3.34, 17.69)	**0.004**	5.81 (0.90, 10.71)	-6.40 (-10.94, -1.85)	12.20 (5.51, 18.89)	**<0.001**
At 24 months	8.77 (3.79, 13.76)	-2.02 (-6.96, 2.55)	10.98 (4.09, 17.86)	**0.002**	13.64 (9.04, 18.24)	-4.67 (-8.85, -0.49)	18.31 (12.10, 24.53)	**<0.001**

[a] Calculated using time*group interaction term, indicating a differential increase from baseline to 4 weeks, baseline to 8 weeks, baseline to 6 months, baseline to 12 months, or baseline to 24 months; Covariates include sex and baseline leisure-time activity adjusted; All the values presented in the table are model-based estimates.

© 2023 Li N et al. *JAMA Network Open.*

eTable 5. Differences in Leisure-time Activity According to Sex[a]

Outcomes	Male				Female			
	Increase from baseline, Mean (95%CI)		Intervention effect Mean (95%CI)	*P*-value	Increase from baseline, Mean (95%CI)		Intervention effect Mean (95%CI)	*P*-value
	Intervention group	Control group			Intervention group	Control group		
Leisure-time activity								
At 4 weeks	12.40 (7.32, 17.47)	-5.03 (-9.24, -0.81)	17.43 (10.83, 24.02)	**<0.001**	8.68 (4.59, 12.76)	0.62 (-3.36, 4.60)	8.06 (2.36, 13.76)	**0.006**
At 8 weeks	13.26 (8.27, 18.25)	-3.00 (-7.23, 1.24)	16.25 (9.71, 22.80)	**<0.001**	10.18 (6.55, 13.81)	-1.84 (-5.47, 1.79)	12.16 (7.01, 17.31)	**<0.001**
At 6 months	14.12 (8.03, 20.22)	-0.02 (-5.24, 5.20)	14.15 (6.12, 22.18)	**<0.001**	11.11 (6.64, 15.58)	-1.09 (-5.59, 3.40)	12.21 (5.88, 18.54)	**<0.001**
At 12 months	6.84 (1.57, 12.10)	-5.78 (-10.28, -1.28)	12.61 (5.69, 19.54)	**<0.001**	7.45 (2.60, 12.29)	-2.91 (-7.84, 2.02)	10.35 (3.44, 17.26)	**0.004**
At 24 months	9.53 (4.56, 14.49)	-6.84 (-11.09, -2.59)	16.37 (9.83, 22.90)	**<0.001**	12.79 (8.20, 17.38)	-0.65 (-5.16, 3.86)	13.44 (7.03, 19.85)	**<0.001**

[a] Calculated using time*group interaction term, indicating a differential increase from baseline to 4 weeks, baseline to 8 weeks, baseline to 6 months, baseline to 12 months, or baseline to 24 months; Covariates include age and baseline leisure-time activity adjusted; All the values presented in the table are model-based estimates.

© 2023 Li N et al. *JAMA Network Open.*

eTable 6. Differences in Leisure-time Activity According to BMI[a,b]

Outcomes	Underweight/Normal weight				Overweight/Obesity			
	Increase from baseline, Mean (95%CI)		Intervention effect Mean (95%CI)	*P*-value	Increase from baseline, Mean (95%CI)		Intervention effect Mean (95%CI)	*P*-value
	Intervention group	Control group			Intervention group	Control group		
Leisure-time activity								
At 4 weeks	10.55 (5.63, 15.47)	-2.78 (-7.07, 1.52)	13.32 (6.79, 19.85)	**<0.001**	9.81 (5.69, 13.92)	-1.34 (-5.30, 2.62)	11.15 (5.41, 16.89)	**<0.001**
At 8 weeks	11.55 (7.06, 16.04)	-3.75 (-7.76, 0.25)	15.30 (9.29, 21.32)	**<0.001**	11.45 (7.53, 15.37)	-0.60 (-4.41, 3.20)	12.05 (6.58, 17.51)	**<0.001**
At 6 months	14.34 (8.59, 20.08)	-0.03 (-5.26, 5.21)	14.40 (6.64, 22.15)	**<0.001**	10.43 (5.92, 14.93)	-0.70 (-5.08, 3.68)	11.12 (4.83, 17.41)	**<0.001**
At 12 months	7.87 (2.58, 13.15)	-4.58 (-9.44, 0.29)	12.44 (5.25, 19.64)	**<0.001**	6.70 (1.93, 11.48)	-3.95 (-8.60, 0.70)	10.65 (3.98, 17.31)	**0.002**
At 24 months	12.91 (7.95, 17.86)	-2.49 (-6.92, 1.94)	15.39 (8.76, 22.02)	**<0.001**	10.30 (5.72, 14.87)	-4.09 (-8.54, 0.36)	14.42 (8.02, 20.82)	**<0.001**

Abbreviation: BMI, Body Mass Index.

[a] Calculated as weight in kilograms divided by height in square meters; Weight was determined using an electronic scale, while height was obtained by a portable stadiometer; Underweight/normal weight was defined by BMI of less than 24, and overweight/obesity was defined by BMI of at least 24 according to Chinese reference.

[b] Calculated using time*group interaction term, indicating a differential increase from baseline to 4 weeks, baseline to 8 weeks, baseline to 6 months, baseline to 12 months, or baseline to 24 months; Covariates include age, sex, and baseline leisure-time activity adjusted; All the values presented in the table are model-based estimates.

© 2023 Li N et al. *JAMA Network Open.*

eTable 7. Effects of the Intervention and Time on Secondary Outcomes of Participants[a] (Intention-to-Treat Method Using Multiple Imputed Data)

Secondary Outcomes	Increase from baseline, Mean (95% CI)		Intervention effect	*P*-Value
	Intervention group	Control group	Mean (95% CI)	
Proportions of meeting WHO recommendations, %				
At 4 weeks	31.70 (31.15, 32.25)	7.57 (7.02, 8.12)	24.01 (23.44, 24.58)	**<0.001**
At 8 weeks	23.64 (23.09, 24.19)	2.97 (2.42, 3.51)	20.64 (20.09, 21.09)	**<0.001**
At 6 months	23.67 (23.12, 24.21)	4.71 (4.17, 5.26)	18.94 (18.39, 19.49)	**<0.001**
At 12 months	8.97 (8.42, 9.52)	0.82 (0.27, 1.37)	8.12 (7.55, 8.69)	**<0.001**
At 24 months	2.73 (2.20, 3.31)	-3.17 (-3.73, -2.61)	5.85 (5.30, 6.40)	**<0.001**
Leisure-time sitting, min/day				
At 4 weeks	4.48 (-14.59, 23.54)	46.22 (28.96, 63.47)	-41.74 (-67.46, -16.03)	**0.001**
At 8 weeks	21.41 (2.92, 39.90)	58.97 (41.84, 76.11)	-37.56 (-62.77, -12.36)	**0.004**
At 6 months	35.56 (17.12, 53.99)	38.09 (20.83, 55.34)	-2.53 (-27.78, 22.72)	0.84
At 12 months	21.68 (3.06, 40.31)	29.82 (12.22, 47.41)	-8.13 (-33.75, 17.49)	0.53
At 24 months	34.58 (15.76, 53.41)	64.03 (46.58, 81.49)	-29.45 (-55.12, -3.78)	**0.02**
Household activity				
At 4 weeks	3.01 (-2.27, 8.29)	-0.40 (-5.18, 4.38)	3.41 (-3.71, 10.54)	0.35
At 8 weeks	1.16 (-3.96, 6.28)	-3.00 (-7.74, 1.75)	4.16 (-2.83, 11.14)	0.24
At 6 months	3.84 (-1.27, 8.94)	-5.81 (-10.59, -1.03)	9.65 (2.65, 16.64)	**0.007**
At 12 months	-2.26 (-7.41, 2.90)	-4.58 (-9.46, 0.29)	2.33 (-4.77, 9.43)	0.52
At 24 months	-0.0003(-5.22, 5.21)	-5.49 (-10.33, -0.66)	5.49 (-1.62, 12.61)	0.13
Work-related activity				
At 4 weeks	-9.12 (-17.26, -0.97)	-14.61 (-21.98, -7.25)	5.50 (-5.48, 16.48)	0.33
At 8 weeks	-23.06 (-30.96, -15.17)	-20.08 (-27.40, -12.77)	-2.99 (-13.75, 7.77)	0.59
At 6 months	-2.72 (-10.59, 5.15)	-1.54 (-8.91, 5.83)	-1.18 (-11.96, 9.60)	0.83
At 12 months	7.50 (-0.45, 15.46)	9.03 (1.52, 16.54)	-1.52 (-12.46, 9.41)	0.78
At 24 months	-7.94 (-15.98, 0.10)	0.38 (-7.07, 7.83)	-8.34 (-19.30, 2.63)	0.14
Total physical activity				
At 4 weeks	3.40 (-6.69, 13.49)	-17.11 (-26.24, -7.98)	20.51 (6.90, 34.12)	**0.003**
At 8 weeks	-10.90 (-20.68, -1.11)	-25.71 (-34.77, -16.65)	14.81 (1.48, 28.15)	**0.03**
At 6 months	12.98 (3.22, 22.73)	-7.47 (-16.60, 1.66)	20.45 (7.09, 33.81)	**0.003**
At 12 months	12.42 (2.57, 22.28)	0.15 (-9.16, 9.45)	12.28 (-1.28, 25.83)	0.08
At 24 months	2.83 (-7.13, 12.79)	-8.81 (-18.05, 0.42)	11.64 (-1.94, 25.23)	0.09

[a] Model-based results from general and generalized linear mixed models. Calculated using time*group interaction term, indicating a differential increase from baseline to 4 weeks, baseline to 8 weeks, baseline to 6 months, baseline to 12 months, or baseline to 24 months; Covariates include age, sex, and the respective baseline value of the outcome of interest adjusted.

© 2023 Li N et al. *JAMA Network Open.*

eTable 8. Effects of the Intervention and Time on Health Outcomes of Participants[a]

Health Outcomes	Increase from baseline, Mean (95% CI)		Intervention effect	*P*-Value
	Intervention group	Control group	Mean (95% CI)	
Cognitive function[b]				
At 4 weeks	1.13 (0.80, 1.47)	1.54 (1.24, 1.85)	-0.41 (-0.86, 0.04)	0.08
At 8 weeks	1.58 (1.26, 1.91)	1.68 (1.38, 1.99)	-0.10 (-0.54, 0.34)	0.66
At 6 months	1.71 (1.38, 2.03)	1.67 (1.36, 1.97)	0.04 (-0.40, 0.48)	0.86
At 12 months	1.91 (1.58, 2.24)	1.55 (1.24, 1.86)	0.36 (-0.09, 0.81)	0.12
At 24 months	1.90 (1.57, 2.23)	1.61 (1.30, 1.92)	0.29 (-0.16, 0.74)	0.21
Night-time sleep quality[c]				
At 4 weeks	-0.43 (-0.93, 0.06)	-0.03 (-0.48, 0.42)	-0.41 (-1.07, 0.26)	0.24
At 8 weeks	-0.81 (-1.29, -0.33)	0.24 (-0.20, 0.68)	-1.05 (-1.71, -0.40)	**0.002**
At 6 months	-0.73 (-1.21, -0.26)	-0.06 (-0.50, 0.39)	-0.68 (-1.33, -0.02)	**0.04**
At 12 months	-0.21 (-0.69, 0.28)	0.67 (0.21, 1.13)	-0.88 (-1.54, -0.21)	**0.01**
At 24 months	-0.07 (-0.57, 0.42)	0.57 (0.12, 1.03)	-0.65 (-1.32, 0.02)	0.06
Systolic blood pressure[d], mmHg				
At 4 weeks	-4.92 (-7.32, -2.52)	-6.94 (-9.17, -4.70)	2.02 (-1.26, 5.30)	0.23
At 8 weeks	-7.95 (-10.28, -5.63)	-9.75 (-11.97, -7.53)	1.80 (-1.42, 5.01)	0.27
At 6 months	-2.24 (-4.56, 0.07)	-1.08 (-3.33, 1.16)	-1.16 (-4.38, 2.06)	0.48
At 12 months	4.03 (1.70, 6.36)	2.25 (-0.03, 4.52)	1.79 (-1.47, 5.04)	0.28
At 24 months	1.80 (-0.57, 4.16)	1.10 (-1.16, 3.36)	0.70 (-2.57, 3.97)	0.68
Diastolic blood pressure[d], mmHg				
At 4 weeks	-1.00 (-2.31, 0.32)	-1.89 (-3.12, -0.67)	0.89 (-0.90, 2.69)	0.33
At 8 weeks	-1.52 (-2.79, -0.24)	-1.87 (-3.09, -0.66)	0.36 (-1.41, 2.12)	0.69
At 6 months	0.91 (-0.36, 2.18)	0.55 (-0.69, 1.78)	0.36 (-1.41, 2.13)	0.69
At 12 months	0.02 (-1.26, 1.29)	2.79 (1.54, 4.04)	-2.77 (-4.56, -0.98)	**0.002**
At 24 months	0.49 (-0.81, 1.79)	0.49 (-0.75, 1.73)	0.001 (-1.79, 1.79)	0.99
Self-rated heath[e]				
At 4 weeks	1.79 (-0.55, 4.14)	-1.80 (-3.93, 0.32)	3.60 (0.43, 6.76)	**0.03**
At 8 weeks	3.48 (1.21, 5.75)	-0.60 (-2.71, 1.51)	4.07 (0.97, 7.18)	**0.01**
At 6 months	2.90 (0.63, 5.17)	-1.41 (-3.54, 0.72)	4.31 (1.19, 7.42)	**0.007**
At 12 months	1.43 (-0.86, 3.73)	-1.90 (-4.07, 0.27)	3.33 (0.17, 6.50)	**0.04**
At 24 months	3.55 (1.24, 5.87)	-1.51 (-3.66, 0.64)	5.06 (1.90, 8.23)	**0.002**

[a] Model-based results from general and generalized linear mixed models. Calculated using time*group interaction term, indicating a differential increase from baseline to 4 weeks, baseline to 8 weeks, baseline to 6 months, baseline to 12 months, or baseline to 24 months; Covariates include age, sex, and the respective baseline value of the outcome of interest adjusted.

[b] Measured by the Telephone Interview for Cognitive Status (TICS-10). Scores range from 0 to 20, with higher scores indicating better cognition.

[c] Measured by the Pittsburgh Sleep Quality Index (PSQI). Scores range from 0 to 21, with higher scores indicating poorer sleep quality.

[d] Blood pressure was measured by the calibrated Omron U30 electronic sphygmomanometers from the right hands of participants seated down. Three measurements should be taken at least 5 minutes apart, the average of the second two readings was calculated for systolic and diastolic pressure. Exclude the participants not taking their medication regularly.

[e] Measured by the EQ-5D visual analog scale. Scores range from 0 to 100, with higher scores indicating better self-reported health status.

© 2023 Li N et al. *JAMA Network Open.*

eTable 9. Details of Unexpected Serious Adverse Events

Study number	Allocation	Type	Related to intervention
3026	Intervention	Deceased-traffic accident	Not related
2032	Intervention	Deceased-lung cancer	Not related
4026	Intervention	Deceased-unknown cause	Not related
2054	Intervention	Deceased-unknown cause	Not related
2026	Intervention	Deceased-unknown cause	Not related
5086	Control	Deceased-heart attack	Not related
5068	Control	Deceased-cerebral hemorrhage	Not related

© 2023 Li N et al. *JAMA Network Open.*
