# Supplementary material for: Physical Activity Intervention for Leisure-Time Activity Levels Among Older Adults: A Cluster Randomized Trial
Source: JAMA Netw Open. 2023 Sep 15;6(9):e2333195. doi: 10.1001/jamanetworkopen.2023.33195 (PMC10504609; doi:10.1001/jamanetworkopen.2023.33195)
Supplement: Supplement 3. — Data Sharing Statement [file jamanetwopen-e2333195-s003.pdf]

Data Sharing Statement

Li. Physical Activity Intervention for Leisure-Time Activity Levels Among Older Adults. *JAMA Netw Open*. Published September 15, 2023. doi:10.1001/jamanetworkopen.2023.33195

Data
Data available: No

Additional Information
Explanation for why data not available: Data collected for the study will be made available publicly upon reasonable request. For further detailed data access policy and procedure, please contact junmin.zhou@scu.edu.cn.
